# Metal‐Free Intermolecular C−H Borylation of *N*‐Heterocycles at B−B Multiple Bonds

**DOI:** 10.1002/anie.202213284

**Published:** 2022-12-22

**Authors:** Tobias Brückner, Benedikt Ritschel, J. Oscar C. Jiménez‐Halla, Felipe Fantuzzi, Dario Duwe, Christian Markl, Rian D. Dewhurst, Maximilian Dietz, Holger Braunschweig

**Affiliations:** ^1^ Institute for Inorganic Chemistry Julius-Maximilians-Universität Würzburg Am Hubland 97074 Würzburg Germany; ^2^ Institute for Sustainable Chemistry & Catalysis with Boron Julius-Maximilians-Universität Würzburg Am Hubland 97074 Würzburg Germany; ^3^ Department of Chemistry Division of Natural and Exact 36050 Guanajuato Mexico; ^4^ School of Chemistry and Forensic Science University of Kent Park Wood Rd Canterbury CT2 7NH UK

**Keywords:** Boron, Borylation, Carbene, Diboryne, Hydroarylation

## Abstract

Carbene‐stabilized diborynes of the form LBBL (L=*N*‐heterocyclic carbene (NHC) or cyclic alkyl(amino)carbene (CAAC)) induce rapid, high yielding, intermolecular ortho‐C−H borylation at *N*‐heterocycles at room temperature. A simple pyridyldiborene is formed when an NHC‐stabilized diboryne is combined with pyridine, while a CAAC‐stabilized diboryne leads to activation of two pyridine molecules to give a tricyclic alkylideneborane, which can be forced to undergo a further H‐shift resulting in a zwitterionic, doubly benzo‐fused 1,3,2,5‐diazadiborinine by heating. Use of the extended *N*‐heteroaromatic quinoline leads to a borylmethyleneborane under mild conditions via an unprecedented boron‐carbon exchange process.

## Graphical abstract

Carbene‐stabilized diborynes of the form LBBL (L=*N*‐heterocyclic carbene or cyclic alkyl(amino)carbene) induce rapid, high yielding, intermolecular ortho‐C−H borylation at *N*‐heterocycles at room temperature. The reaction initially provides the simple hydroarylated diborene, but in some cases proceeds further, providing unusual 1,3,2,5‐diazadiborinine and base‐stabilized alkylidene products.

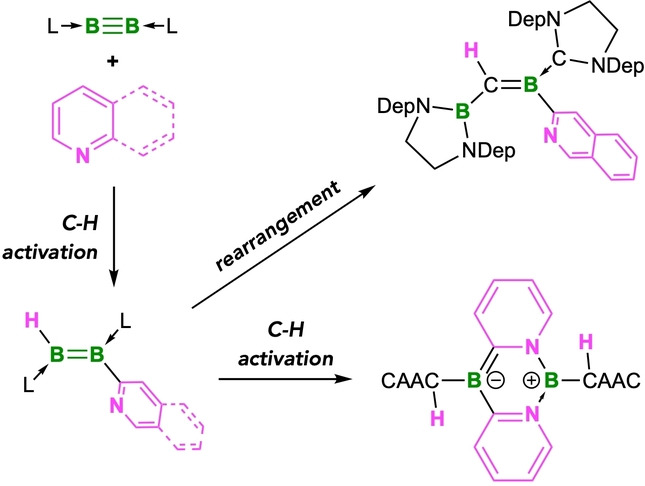

The now‐ubiquitous Suzuki–Miyaura cross‐coupling reaction,[^1^, ^2^] and the growing realization that C−B bonds can act as near‐universal placeholders for the functionalization of organic molecules,[^3^, ^4^] has spurred enormous interest in the efficient synthesis of borylated organics. Transition‐metal catalyzed C−H borylation has emerged as a promising, direct, and often selective route to borylated precursors for Suzuki–Miyaura cross‐coupling reactions.[^5^, ^6^] However, the toxicity and environmental impact of the transition metals used in catalysis, and the expense related to their removal from the products, has caused concern in the chemical industry. Consequently, the search for metal‐free C−H borylation protocols has become a hotly‐contested area of research,[^7^, ^8^, ^9^, ^10^, ^11^, ^12^, ^13^] however, this chemistry is hampered by the relative inertness of most C−H bonds and chemo‐/regioselectivity issues arising from the multiple C−H sites present in most target molecules. In particular, protocols for selective C−H borylation of heterocyclic compounds with relatively reactive auxiliary sites, such as the N atoms of pyridines, present further synthetic challenges, and are exceedingly rare even with the assistance of transition‐metal catalysts.[^14^, ^15^]

The recent development of highly reactive molecules containing B−B multiple bonding[^16^, ^17^, ^18^] provides interesting opportunities for novel bond activation reactions. Indeed, doubly Lewis‐base‐stabilized diborynes, of the form [LB≡BL] (L=Lewis base such as *N*‐heterocyclic carbenes or cyclic alkyl(amino)carbenes), have already been shown to undertake a number of interesting intermolecular bond activation reactions, leading to 1,2‐additions across their B≡B triple bonds. These include the H−H bond of dihydrogen,^[19]^ the C−O bonds of CO and CO_2_,[^20^, ^21^] B−H^[22]^ and B−B^[23]^ bonds, S−S and Se−Se bonds,^[24]^ and even the activated C−H bonds of acetone and alkynes.[^25^, ^26^] The demonstrably high reactivity of diborynes makes them good candidates for the highly challenging task of activating the C−H bonds of (hetero)arenes, prompting us to combine these two classes of reagents in this work.

Herein we report three different modes of regioselective, intermolecular C−H borylation of *N*‐heterocycles with carbene‐stabilized diborynes, compounds with varying degrees of boron‐boron multiple bonding.[^16^, ^17^] All of these reactions occur at ambient temperature and in the absence of catalysts or additives. Depending on the diboryne precursor, either one or two molecules of pyridine can be activated, leading either to a simple pyridyldiborene or a tricyclic alkylideneborane, respectively. Use of the larger heteroaromatic quinoline leads initially to the simple C−H borylation product, which spontaneously undergoes a highly unusual B/C exchange, leading to a borylmethyleneborane.

The doubly carbene‐stabilized diborynes [(SIDep)B≡B(SIDep)] (**1**, SIDep=1,3‐bis(2,6‐diethylphenyl)‐imidazolin‐2‐ylidene) and [(CAAC)B≡B(CAAC)] (**2**, Scheme 1; CAAC=1‐(2,6‐di*iso*propylphenyl)‐3,3,5,5‐tetramethyl‐pyrrolidin‐2‐ylidene) have thus far shown the most facile reactivity of all of the known species of the form LB≡BL.[^16^, ^19^] Consequently, we chose these two species in initial reactivity tests with *N*‐heteroaromatics. Previous results have indicated that strongly‐binding ligands such as NHCs and CO can form adducts with diborynes such as **2**,[^27^, ^28^, ^29^] suggesting that pyridines and their derivatives could potentially form similar adducts of the form [LBB(pyr)L] (pyr=N‐bound pyridine derivative) with **1** or **2**.

**Scheme 1:**
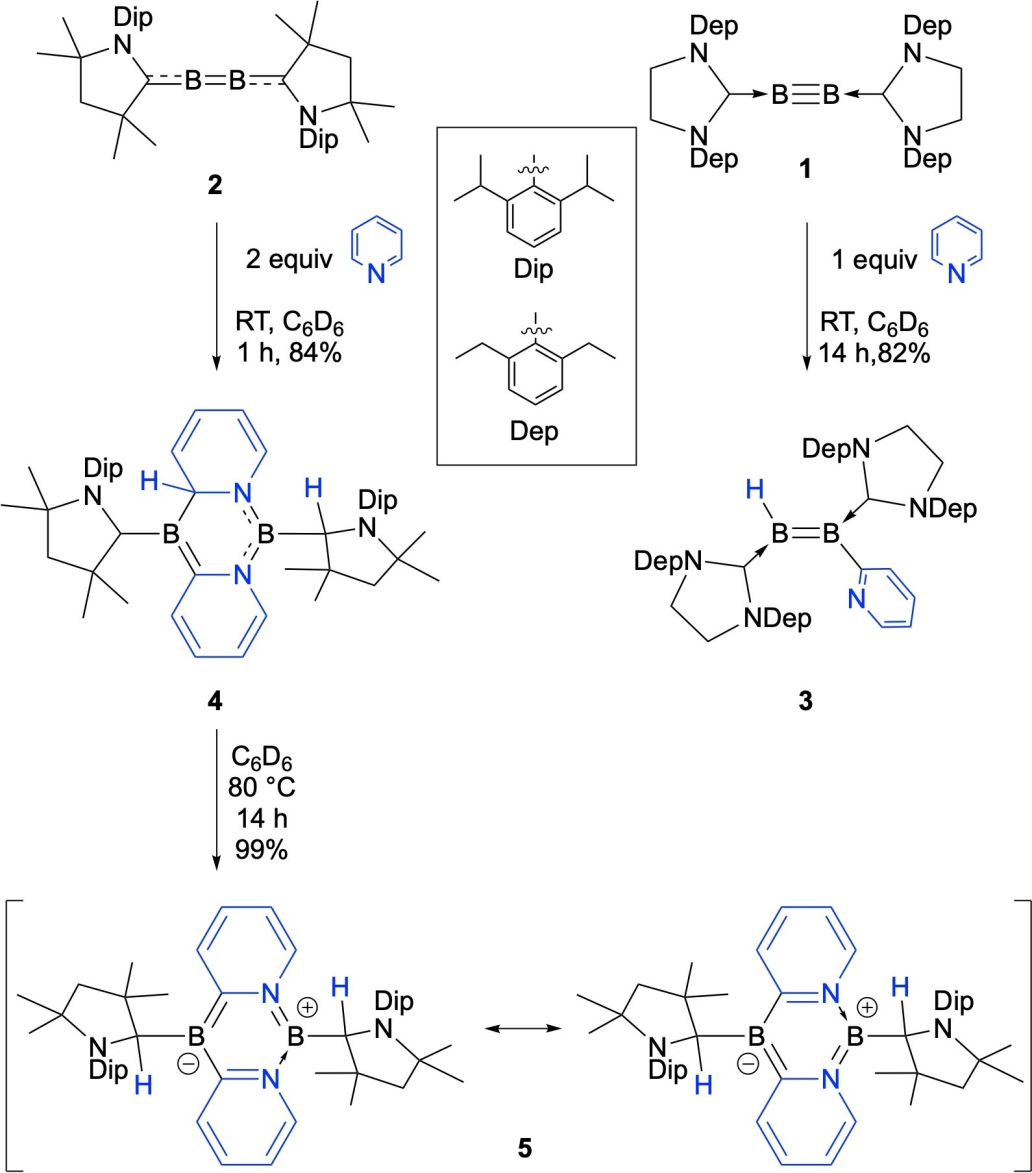
Single and double C−H borylation of diborynes.

Thereby, treatment of **1** with an equimolar amount or an excess of pyridine led to an immediate color change from red to blue and a new set of ^11^B NMR signals at 35 and 25 ppm (**1**: *δ*(^11^B)=56 ppm). After evaporation of all volatiles under high vacuum and washing with hexane, the blue solid **3** was isolated in 82 % yield (Scheme 1). A single‐crystal X‐ray diffraction (SCXRD) study unequivocally revealed **3** to be not a simple pyridine adduct of **1** but a doubly base‐stabilized 1‐hydro‐2‐pyridyldiborene, suggesting *ortho*‐C−H borylation of pyridine (Figure 1).^[30]^ A signal corresponding to the boron‐bound hydrogen atom was detected in the ^11^B‐decoupled ^1^H NMR spectrum of **3** at 3.35 ppm as a broad singlet. Apart from those corresponding to the carbene carbon nuclei, the most low‐field ^13^C NMR resonance can be assigned to the boron‐bound carbon atom of the pyridyl substituent (180.3 ppm), identified by a 2D ^13^C,^1^H heteronuclear multiple bond correlation (HMBC) NMR experiment. The solid‐state structure of diborene **3** (Figure 1, left) shows a B1−B2 distance of 1.591(5) Å, lying in the expected range for doubly NHC stabilized diborenes.[^19^, ^23^, ^31^] The nearly identical B1−C1 (1.546(5) Å) and B2−C2 (1.563(5) Å) distances, the distinct B2−C3 single bond (1.589(5) Å), as well as the ca. 50° twist of the pyridyl ring from the central diborene plane, suggest negligible π‐delocalization between the B=B and pyridyl groups. This is supported by DFT‐calculated molecular orbitals (MOs) of **3**, with both the HOMO and LUMO resembling those of conventional doubly NHC‐stabilized diborenes (Figure 2). The HOMO displays delocalization of π electron density over the C^NHC^−B=B−C^NHC^ axis, while the LUMO shows π* antibonding character at the B−B bond and π bonding character at the B−C^NHC^ bonds.

**Figure 1:**
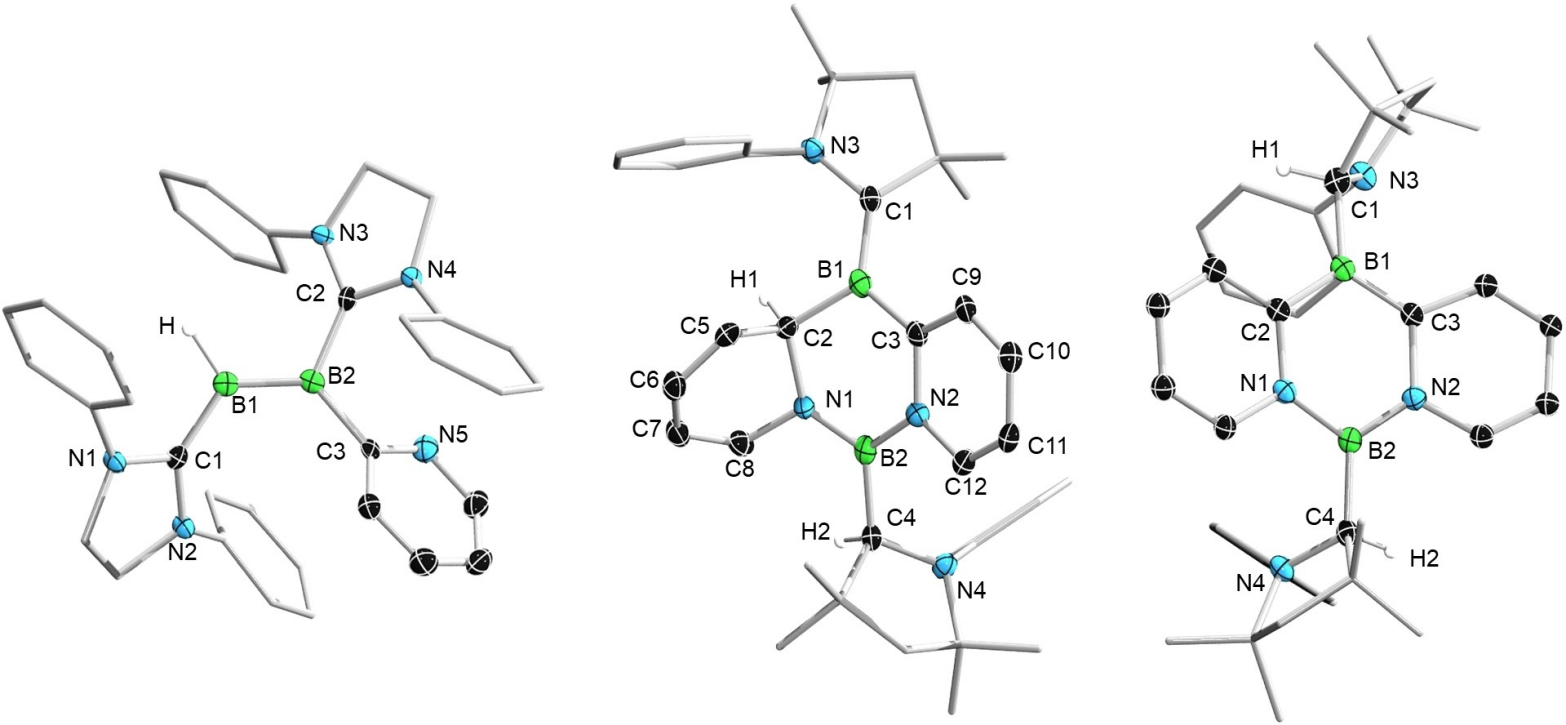
Crystallographically derived structures of **3** (left), **4** (middle) and **5** (right, crystallized from benzene). Ellipsoids shown at the 50 % probability level. Ellipsoids of peripheral groups, the di*iso*propyl groups of the CAAC ligand of **4** and **5**, the ethyl groups of the SIDep ligand of **3**, most hydrogen atoms, and all solvent molecules have been removed for clarity. Selected bond lengths [Å] for **3**: C1−B1 1.546(5), B1−B2 1.591(5), B2−C2 1.563(5), B2−C3 1.589(5), C3−N5 1.380(4). For **4**: C1−B1 1.521(4), C1−N3 1.393(4), B1−C2 1.611(4), B1−C3 1.507(5), C2−C5 1.504(4), C5−C6 1.342(4), C6−C7 1.450(4), C7−C8 1.328(4), C8−N1 1.421(4), N1−C2 1.505(4), N1−B2 1.402(4), B2−N2 1.477(4), B2−C4 1.615(4), N2−C3 1.433(3), C3−C9 1.425(4), C9−C10 1.359(4), C10−C11 1.425(4), C11−C12 1.346(4), N2−C12 1.387(4). Disorder of **5** led to low precision in its solid‐state structure, precluding detailed discussion of structural parameters. Calculated bond lengths [Å] for **5** at the ωB97X‐D/6‐31G(d,p) level: B1−C2 1.507, B1−C3 1.506, C2−N1 1.407, N1−B2 1.446, B2−N2 1.451, N2−C3 1.407.

**Figure 2:**
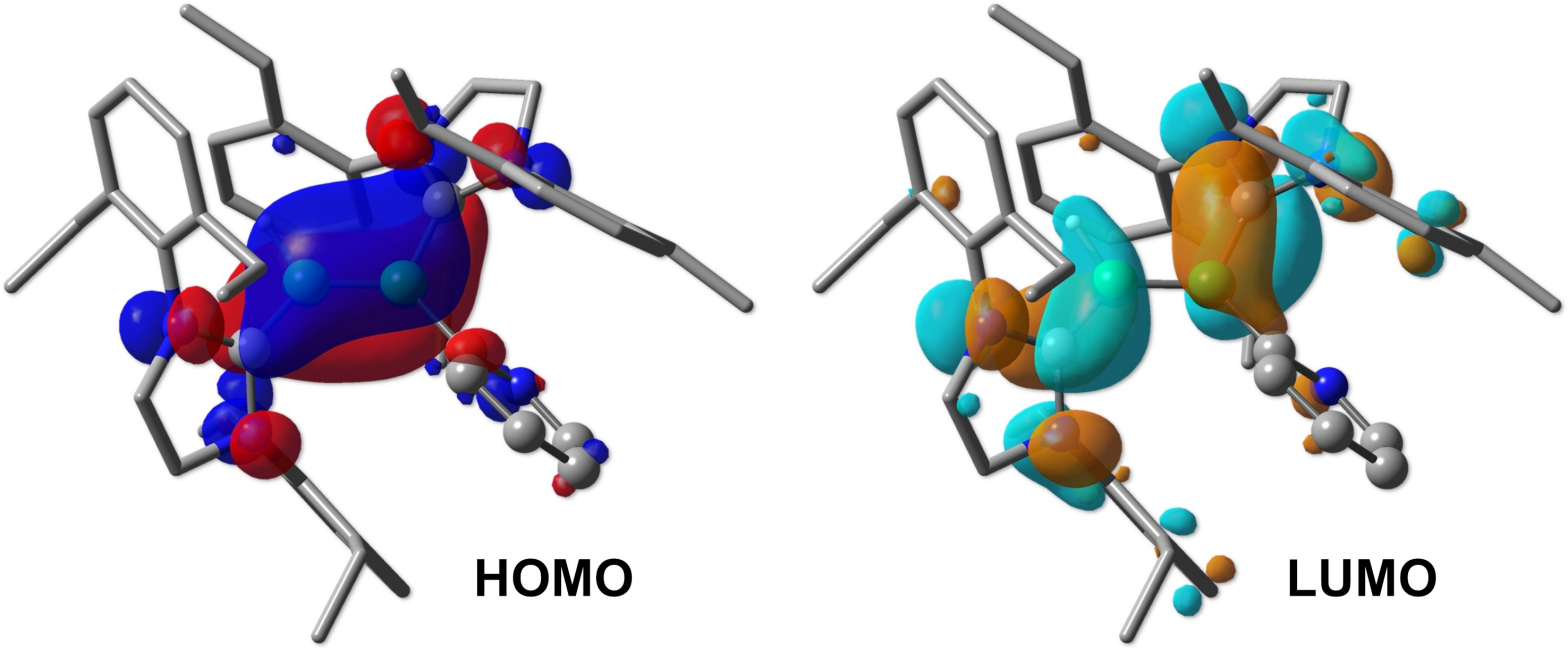
Canonical Kohn–Sham MOs of **3**. Level of theory: ωB97X‐D/6‐311++G(d,p)//ωB97X‐D/6‐31G(d,p). Selected hydrogen atoms are omitted for clarity. Isovalue: 0.03.

While combining CAAC‐stabilized diboryne **2** with one equivalent of pyridine led to roughly half of the precursor remaining unreacted, adding two equivalents of pyridine to a benzene solution of **2** (Scheme 1) at room temperature led to a color change from purple to pink within one hour. The ^11^B NMR spectrum of the reaction mixture displayed new resonances at 32 and 22 ppm, upfield of those of the starting material (**1**: *δ*(^11^B)=80 ppm). After workup, a purple solid was obtained in 82 % yield. An SCXRD study revealed the compound to be the tricyclic diazadiborinine derivative **4**, resulting from activation of two pyridine molecules. The ^1^H NMR spectrum of **4** shows, in addition to expected aromatic signals of the CAAC ligands, only four additional protons in this region, with an additional set of signals found in the alkene region (5.89–5.54 ppm), confirming the loss of aromaticity of one pyridyl group and consequent formation of a butadiene‐type structural motif. A signal at 4.26 ppm can be assigned to the hydrogen atom now bound to a former carbene carbon atom (H2 in Figure 1, middle), in line with previous observations of H‐shifts onto CAAC ligands,^[32]^ as well as results observed during element‐hydrogen bond activations induced by CAAC itself.^[33]^ A broad ^1^H NMR spectroscopic signal at 3.33 ppm (H1 in Figure 1, middle) shows a cross‐signal to a resonance in the ^13^C,^1^H heteronuclear single quantum coherence (HSQC) NMR spectrum at 65.2 ppm, corresponding to the hydropyridyl carbon atom bound to boron (C2 in Figure 1, middle). The solid‐state structure of **4** (Figure 1, middle) shows a distinct butadiene‐like structure of the hydropyridyl unit, with alternating C−C bond lengths (C5−C6: 1.342(4); C6−C7: 1.450(4); C7−C8 1.328(4) Å), while the aromatic pyridyl unit shows typical bond equilibration (1.425(4)–1.346(4) Å), comparable to those of a recently published CAAC‐stabilized diboraanthracene diradical.^[34]^ These differences are confirmed by the calculated zz components of the shielding tensor nucleus‐independent chemical shift (NICS_zz_(1)) elements (Figure 3, left). Also notable are the B−C distances: while the B1−C2 distance (1.611(4) Å) suggests a single bond, the B1−C3 distance (1.507(4) Å) indicates double bond character and the presence of an alkylideneborane unit.^[35]^ The B1−C1 distance (1.521(4) Å) is in the expected range for a dative CAAC−B interaction with significant π‐bonding character, whereas the B2−C4 (1.615(4) Å) distance suggests a single covalent bond.

**Figure 3:**
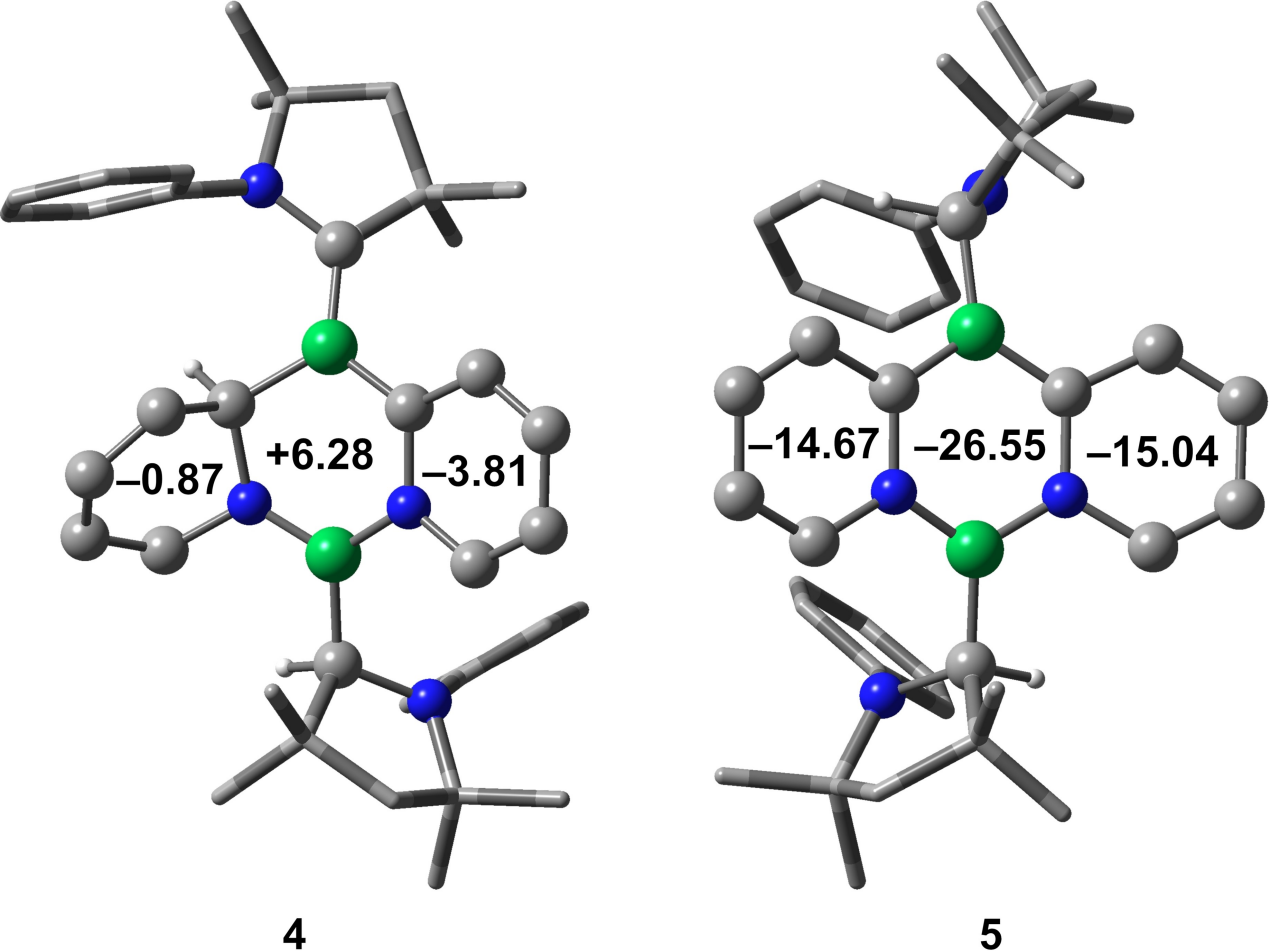
NICS_zz_(1) values (ppm) of the rings of compounds **4** and **5** at the ωB97X‐D/6‐311++G(d,p)//ωB97X‐D/6‐31G(d,p) level of theory. Selected hydrogen atoms are omitted for clarity. For reference, the NICS_zz_(1) value of benzene at the same level is −29.75 ppm, while those of anthracene are −25.37 ppm (outer rings) and −35.09 ppm (central ring).

Heating a C_6_D_6_ solution of **4** at 80 °C for 14 h led to an additional color change of the reaction mixture from light to dark pink, the ^11^B NMR spectrum of which showed only a single resonance at 28 ppm (**4**: *δ*(^11^B)=32, 22 ppm). The purple solid **5** was obtained after workup in nearly quantitative yield, the ^1^H NMR spectrum of which showed the absence of signals in the alkene region and no hydropyridyl signal comparable to that of **3** (*δ*(^1^H)=3.33 ppm). Instead, two signals corresponding to protonated CAAC substituents were observed (4.96 and 4.87 ppm). These data suggested rearomatization of the hydropyridyl unit and a concomitant H‐shift to the remaining CAAC unit (Scheme 1).

An SCXRD study of **5** using crystals obtained from a saturated pentane solution indicated the presence of an essentially planar tricyclic central unit, with the aryl substituents of both CAACH substituents oriented on opposite sides of the tricyclic core. Alternatively, a solid‐state structure derived from crystals prepared using benzene as crystallization medium shows both aryl substituents to be oriented on the same side of the tricyclic core, leading to a butterfly‐like structure with an angle of 21° (Figure 1, right).

Together, these spectroscopic and structural data indicate that **5** is a very rare example of a 1,3,2,5‐diazadiborinine. The high symmetry of **5** results in disorder in the molecular structure shown in Figure 1 (right), whereby a molecule with swapped C and N atoms is superimposed on the first. This disorder led to reduced precision in the structure, prompting us to turn to DFT calculations to gain a better idea of the structure and energetics of **5**. All distances of the central core, both experimental and calculated (1.422–1.532 Å), lie in the range of elongated double bonds, suggesting extended delocalization, similar to results reported by Kinjo et al. for their 1,3,2,5‐diazadiborinines.[^36^, ^37^] The calculated NICS_zz_(1) values (Figure 3, right) of the outer rings of **5** suggest greater aromaticity than those of **4**, with the zwitterionic inner B_2_N_2_C_2_ core being relatively aromatic. These NICS values underscore the similarity of **5** to its purely hydrocarbon analogue anthracene, which is known to exhibit a higher NICS(0) for its central ring relative to the outer rings.^[38]^ Accordingly, the transformation of **4** to **5** is exergonic by −25.9 kcal mol^−1^ based on DFT calculations at the SMD(benzene):ωB97X‐D/6‐311++G(d,p)//ωB97X‐D/6‐31G(d,p) level.

In order to test if diborene **3** also undergoes thermally‐induced reactivity, benzene solutions of **3** were heated independently to 60 °C and 80 °C. However, in both cases this led only to decomposition.

Given the intriguing reactivity of diborynes with monocyclic *N*‐heterocycle pyridine, we sought to expand our scope to bicyclic *N*‐heterocycle quinoline. The reaction of **2** with quinoline gave an inseparable mixture of products, however, treatment of **1** with quinoline resulted in an immediate color change from red to blue, similar to the above reaction of **2** with pyridine. ^11^B NMR spectroscopic resonances at 25 and 30 ppm were observed after a few minutes, suggesting the presence of diborene **6**, analogous to **3**. However, the resonance at 25 ppm had disappeared after 10 minutes, while the signal at ca. 30 ppm had broadened significantly. An additional color change to green occured within one hour, and a near‐complete decoloration took place overnight, the remaining light yellow solution suggesting the absence of diborene in the mixture. After workup by washing the dried reaction mixture with hexane and crystallization from a saturated hexane solution, the product was identified by SCXRD as the borylalkylideneborane **7** (Scheme 2), a constitutional isomer of the presumed intermediate **6** in which one of the carbene carbon atoms has exchanged with one boron atom. The unexpected and highly unusual structure of **7** is confirmed by its NMR spectra. A singlet resonance corresponding to the alkylideneborane C−H proton was found in the ^1^H NMR spectrum at 3.79 ppm, presenting a cross‐signal to a ^13^C NMR resonance at 104.3 ppm in the ^13^C,^1^H HSQC NMR spectrum. This resonance is downfield of typical alkene resonances, but is in the same range as that of a cyclic borylalkylideneborane reported by Berndt et al. (115.2 ppm).^[39]^ The broad resonance observed in the ^11^B NMR spectrum of **7** (30 ppm) can be rationalized by the superposition of two signals.

**Scheme 2:**
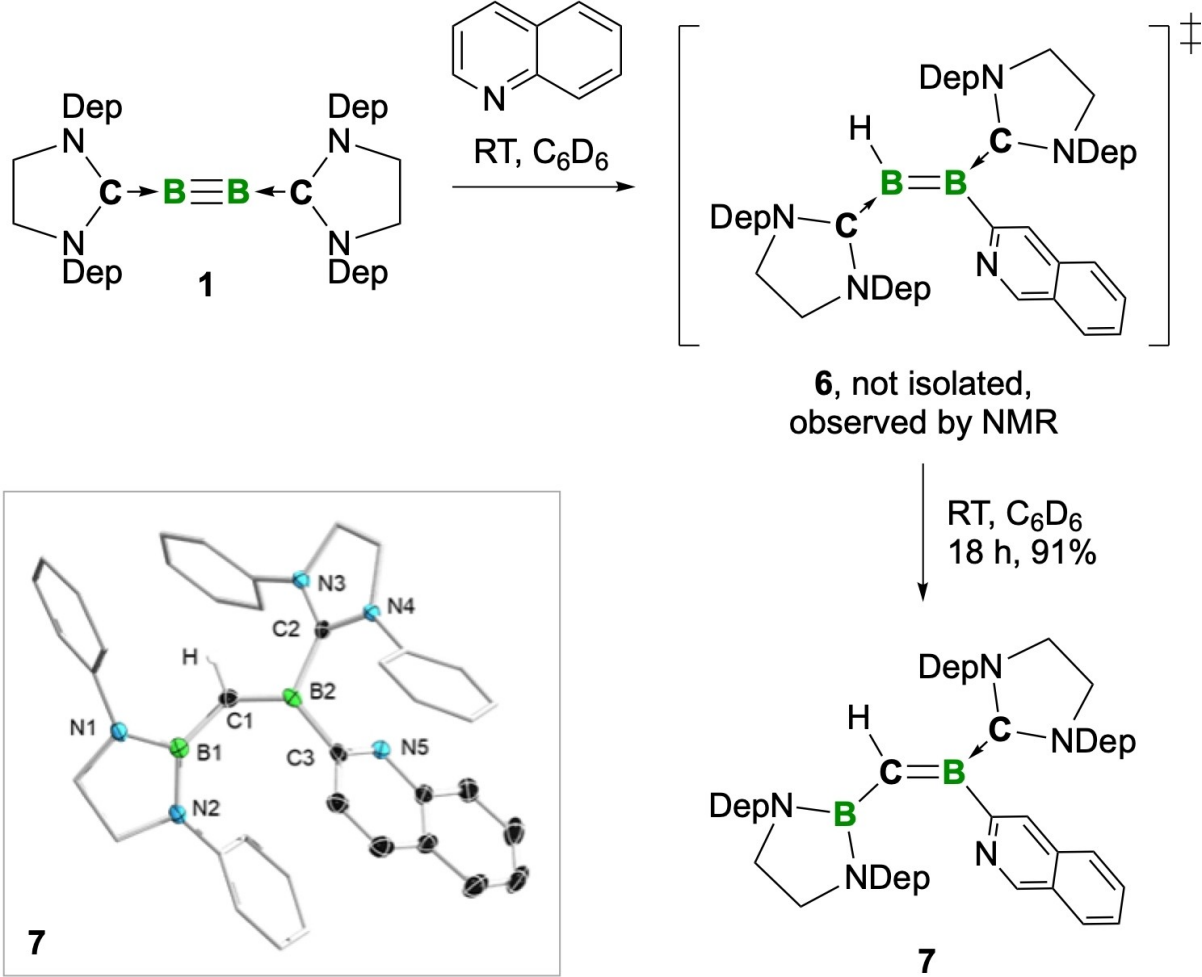
Reaction of **1** with quinoline, forming borylalkylideneborane **7** via diborene **6**. Inset: Crystallographically derived structure of **7**. Ellipsoids shown at the 50 % probability level. Ethyl groups of the SIDep ligand, all hydrogen atoms (except that bound to C1), and ellipsoids of the peripheral groups have been removed for clarity. Selected bond lengths [Å] and torsion angles [°]: N1−B1 1.439(2), N2−B1 1.445(2), B1−C1 1.544(2), C1−B2 1.452(2), B2−C2 1.604(2), B2−C3 1.583(3), C3−N5 1.338(2); N1‐B1‐C1‐B2 173.2.

The alkylideneborane C=B distance (C1−B1) of **7** is 1.452(2) Å, significantly longer than those of structurally characterized (non‐base‐stabilized) alkylideneboranes (1.361(5) Å),^[35]^ but is much more in line with the corresponding distances of base‐stabilized alkylideneboranes (1.431(8) Å)^[40]^ reported in the late 1980s, where the electron deficiency at the boron atoms is saturated by the lone electron pairs of ethers or *N*‐heterocycles. The dative B2−C2 bond (1.604(2) Å) is long relative to those of other compounds with B−SIDep motifs, and suggests negligible π‐interaction. In contrast, the B1−C1 distance (1.544(2) Å) suggests significant delocalization across the B1−C1−B2 unit, which is in accord with the relatively coplanar N1−B1−C1−B2 unit (torsion angle: 173.2°).

Because of the unexpected swapping of B and C atoms in the reaction furnishing **7**, we carried out DFT calculations (SMD(benzene):ωB97X‐D/6‐311++G(d,p) level, see ESI for further details) in order to establish a plausible reaction mechanism for the rearrangement. Our calculations suggest that the reaction starts with the coordination of quinoline at one boron atom of diboryne **2** (Figure 4). Electronically similar to CAAC, the SIDep ligand has enhanced π acidity that allows adoption of a cumulenic structure so that one of the boron atoms can accommodate the lone pair of the quinoline in **2_ad_** (calculated bond lengths [Å]: 1.466 (B−B), 1.464 and 1.461 (B−C) in **2** change to 1.574, 1.429 and 1.492, respectively, in **2_ad_**) with only a small energy difference (Δ*G*_R1_=−2.1 kcal mol^−1^). Then, via transition state **TS_2ad→A1_** (Δ*G*_1_^≠^=16.5 kcal mol^−1^), the low‐coordinate boron atom B2 spontaneously attacks at position 2 of the quinoline, leading to C−H bond activation and a more stable four‐membered ring intermediate **A1** (Δ*G*_R2_=−20.8 kcal mol^−1^). Three subsequent reaction steps then take place rapidly: i) the decoordination of the quinoline nitrogen, through **TS_A1→A2_** (Δ*G*_2_^≠^=13.2 kcal mol^−1^), leading to intermediate **A2** (Δ*G*_R3_=−0.3 kcal mol^−1^); ii) the bridging of a proton between the two boron atoms (**TS_A2→A3_**; Δ*G*_3_^≠^=1.2 kcal mol^−1^) in intermediate **A3** (Δ*G*_R4_=−4.3 kcal mol^−1^); and iii) the complete transfer of the proton to B1 (atom labelling as in the inset of Scheme 2; **TS_A3→6_**; Δ*G*_4_^≠^=4.5 kcal mol^−1^) to obtain the diborene intermediate **6** (Δ*G*_R5_=−17.7 kcal mol^−1^).

**Figure 4:**
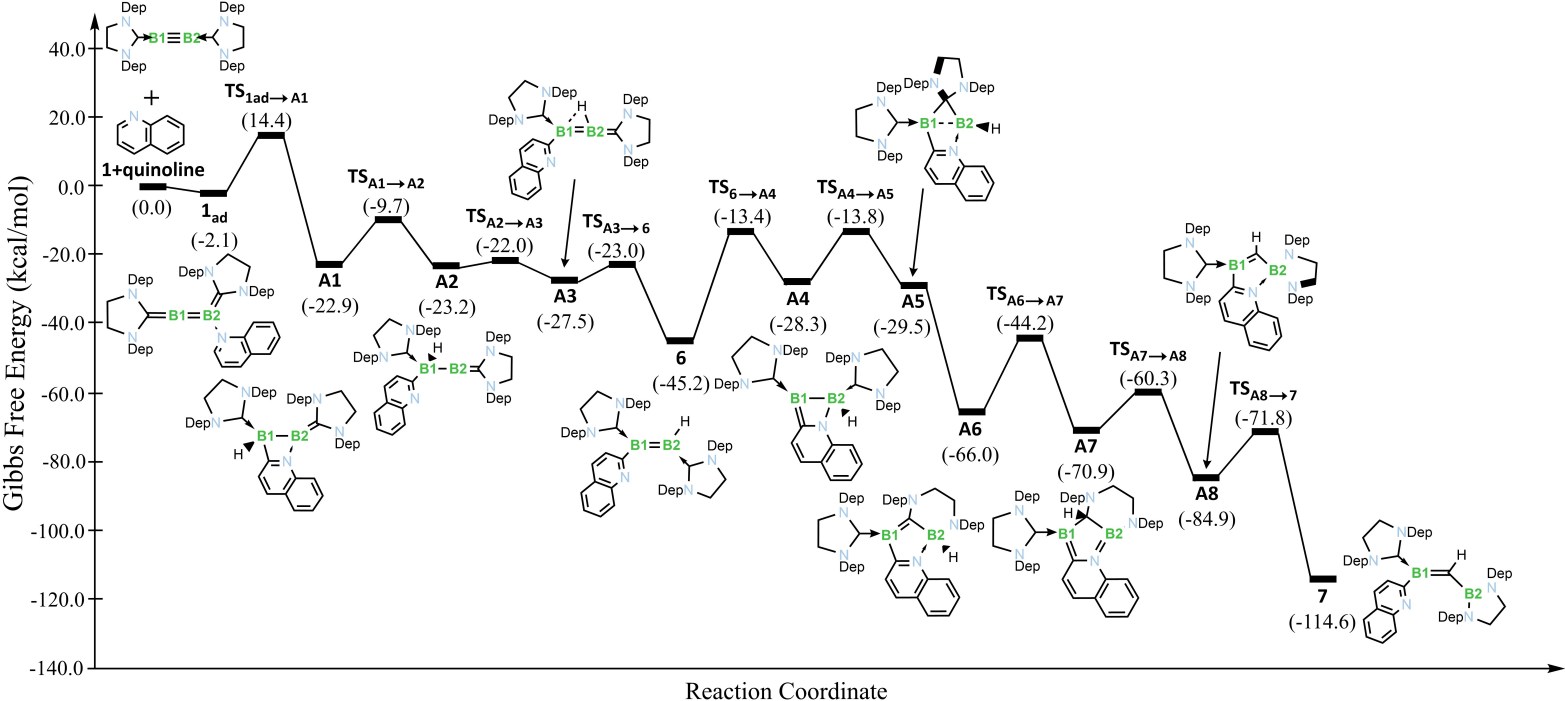
Total energy profile reaction between diboryne **1** and quinoline leading to alkylideneborane product **7**, calculated at the SMD(benzene):ωB97X‐D/6‐311++G(d,p)//ωB97X‐D/6‐31G(d,p) level.

Up to this point, all of the reaction steps are exergonic and conversion occurs by traversing mild energy barriers, suggesting a fast reaction at the beginning. However, diborene **6** lies in a potential well. Reaching intermediate **A4** requires overcoming an energy barrier of Δ*G*_5_^≠^=31.8 kcal mol^−1^ (via **TS_6→A4_**) to again coordinate the quinoline nitrogen atom to the vicinal boron atom B2 (Δ*G*_R6_=+16.9 kcal mol^−1^). However, reverting back to the related intermediate **A1** would require somewhat more energy (**6**→**TS_A1→A2_** requires 35.5 kcal mol^−1^). This deep well is the reason why we were able to detect **6** by NMR that, otherwise, would continue to react faster downhill to product **7**. Moreover, changing quinoline to pyridine increases the energy barrier in **TS_6→A4_** to 39.7 kcal mol^−1^ (higher than the reverse reaction via **TS_A1→A2_**), reflecting an increasing dearomatization effect and terminating the reaction at product **3**. The electron‐rich B1 atom of **A4** then attacks the p orbital of the SIDep ligand bound to B2, leading to spiro compound **A5** through **TS_A4→A5_** (Δ*G*_6_^≠^=14.5 kcal mol^−1^) with a small energetic reward (Δ*G*_R7_=−1.2 kcal mol^−1^). Despite extensive efforts, we were not able to locate a transition state for the next reaction step. The weakened B−B single bond is broken easily and one of the nitrogens of the bridging SIDep ligand undergoes a sigmatropic migration to boron B1 which was rendered more acidic in **A5**.

The large stabilization gained in **A6** (Δ*G*_R8_=−36.5 kcal mol^−1^) can also serve as a driving force to proceed to this side of the potential well. From here, three downhill reaction steps complete the reaction: i) a tautomerization delivers the proton bound to C1 (**TS_A6→A7_**; Δ*G*_7_^≠^=4.5 kcal mol^−1^) which stabilizes **A7** by Δ*G*_R9_=−4.9 kcal mol^−1^; ii) a second sigmatropic rearrangement (**TS_A7→A8_**; Δ*G*_8_^≠^=10.6 kcal mol^−1^) to obtain the spirocyclic boryl species **A8** (Δ*G*_R10_=−14.0 kcal mol^−1^); and finally, iii) decoordination of quinoline from B1 (via **TS_A8→7_**; Δ*G*_9_^≠^=13.1 kcal mol^−1^) to release the alkylideneborane **7** (Δ*G*_R11_=−29.7 kcal mol^−1^). Our proposed reaction mechanism shows this transformation goes through angular polycyclic structures **A6** and **A7**, which are thermodynamically relatively stable. Similar ring‐expanded NHC species resulting from boron insertion have been reported previously,[^41^, ^42^, ^43^, ^44^] but they are generally the final product of the respective reactions, i.e. carbon extrusion to form a 1,3‐diazaborole, as demonstrated here, has not been reported.

In summary, we have demonstrated the catalyst‐free borylation of *N*‐heteroaromatic compounds at carbene‐stabilized diborynes under mild conditions. In one case, treatment with pyridine leads to a simple pyridyldiborene, while increased π‐acidity of the stabilizing carbene ligand results in the activation of two pyridine molecules, leading to a 1,3,2,5‐diazadiborinine via heating of the tricyclic alkylideneborane intermediate. Using quinoline instead of pyridine gives a borylmethyleneborane, resulting from a boron carbon exchange, a plausible mechanism for which was determined by quantum chemical calculations.

## Conflict of interest

The authors declare no conflict of interest.

## Supporting information

As a service to our authors and readers, this journal provides supporting information supplied by the authors. Such materials are peer reviewed and may be re‐organized for online delivery, but are not copy‐edited or typeset. Technical support issues arising from supporting information (other than missing files) should be addressed to the authors.

Supporting Information

Supporting Information

Supporting Information

Supporting Information

Supporting Information

Supporting Information

Supporting Information

Supporting Information

Supporting Information

## Data Availability

The data that support the findings of this study are available from the corresponding author upon reasonable request.
