# Supplementary material for: Metal‐Free Intermolecular C−H Borylation of N‐Heterocycles at B−B Multiple Bonds
Source: Angew Chem Int Ed Engl. 2022 Dec 22;62(5):e202213284. doi: 10.1002/anie.202213284 (PMC10107673; doi:10.1002/anie.202213284)

checkCIF/PLATON report

Structure factors have been supplied for datablock(s) ToBr105_a

THIS REPORT IS FOR GUIDANCE ONLY. IF USED AS PART OF A REVIEW PROCEDURE FOR PUBLICATION, IT SHOULD NOT REPLACE THE EXPERTISE OF AN EXPERIENCED CRYSTALLOGRAPHIC REFEREE.

No syntax errors found. CIF dictionary Interpreting this report

Datablock: ToBr105_a

```
Bond precision:   C-C = 0.0052 A            Wavelength=0.71073

Cell:              a=12.5981(5)     b=13.4918(5)       c=26.2737(11)
                   alpha=90         beta=94.361(2)     gamma=90
Temperature:       100 K

                  Calculated                     Reported
Volume            4452.8(3)                      4452.8(3)
Space group       P 21                           P 21
Hall group        P 2yb                          P 2yb
Moiety formula    C51 H65 B2 N5                  ?
Sum formula       C51 H65 B2 N5                  C51 H65 B2 N5
Mr                769.70                         769.70
Dx,g cm-3         1.148                          1.148
Z                 4                              4
Mu (mm-1)         0.066                          0.066
F000              1664.0                         1664.0
F000'             1664.51
h,k,lmax          15,16,32                       15,16,32
Nref              17559[ 9174]                   16320
Tmin,Tmax         0.982,0.985
Tmin'             0.982

Correction method= Not given

Data completeness= 1.78/0.93        Theta(max)= 26.022

R(reflections)= 0.0485( 13168)                        wR2(reflections)=
                                                      0.1090( 16320)
S = 1.068                     Npar= 1069
```

The following ALERTS were generated. Each ALERT has the format
test-name_ALERT_alert-type_alert-level.
Click on the hyperlinks for more details of the test.

Alert level C

```
STRVA01_ALERT_2_C           Chirality of atom sites is inverted?
           From the CIF: _refine_ls_abs_structure_Flack    1.700
           From the CIF: _refine_ls_abs_structure_Flack_su    1.000
PLAT052_ALERT_1_C Info on Absorption Correction Method   Not Given     Please Do !
PLAT213_ALERT_2_C Atom C73             has ADP max/min Ratio .....        3.1 prolat
PLAT220_ALERT_2_C NonSolvent   Resd 1  C   Ueq(max)/Ueq(min) Range        3.8 Ratio
PLAT220_ALERT_2_C NonSolvent   Resd 2  C   Ueq(max)/Ueq(min) Range        4.3 Ratio
PLAT222_ALERT_3_C NonSolvent Resd 2  H   Uiso(max)/Uiso(min) Range        4.1 Ratio
PLAT340_ALERT_3_C Low Bond Precision on  C-C Bonds ..............    0.00515 Ang.
PLAT907_ALERT_2_C Flack x > 0.5, Structure Needs to be Inverted? .      1.70 Check
PLAT910_ALERT_3_C Missing # of FCF Reflection(s) Below Theta(Min).         9 Note
PLAT911_ALERT_3_C Missing FCF Refl Between Thmin & STh/L=    0.600         8 Report
PLAT913_ALERT_3_C Missing # of Very Strong Reflections in FCF ....         5 Note
```

Alert level G

```
PLAT032_ALERT_4_G Std. Uncertainty on Flack Parameter Value High .      1.000 Report
PLAT720_ALERT_4_G Number of Unusual/Non-Standard Labels .........          2 Note
PLAT883_ALERT_1_G No Info/Value for _atom_sites_solution_primary .     Please Do !
PLAT916_ALERT_2_G Hooft y and Flack x Parameter Values Differ by .       0.10 Check
PLAT978_ALERT_2_G Number C-C Bonds with Positive Residual Density.          0 Info
```

0 **ALERT level A** = Most likely a serious problem - resolve or explain
0 **ALERT level B** = A potentially serious problem, consider carefully
11 **ALERT level C** = Check. Ensure it is not caused by an omission or oversight
5 **ALERT level G** = General information/check it is not something unexpected

2 ALERT type 1 CIF construction/syntax error, inconsistent or missing data
7 ALERT type 2 Indicator that the structure model may be wrong or deficient
5 ALERT type 3 Indicator that the structure quality may be low
2 ALERT type 4 Improvement, methodology, query or suggestion
0 ALERT type 5 Informative message, check

Validation response form

Please find below a validation response form (VRF) that can be filled in and pasted into your CIF.

```
# start Validation Reply Form
_vrf_STRVA01_ToBr105_a
;
PROBLEM: Chirality of atom sites is inverted?
RESPONSE: ...
;
_vrf_PLAT052_ToBr105_a
;
PROBLEM: Info on Absorption Correction Method   Not Given     Please Do !
RESPONSE: ...
```

```
;
_vrf_PLAT213_ToBr105_a
;
PROBLEM: Atom C73              has ADP max/min Ratio .....        3.1 prolat
RESPONSE: ...
;
_vrf_PLAT220_ToBr105_a
;
PROBLEM: NonSolvent   Resd 1  C   Ueq(max)/Ueq(min) Range        3.8 Ratio
RESPONSE: ...
;
_vrf_PLAT222_ToBr105_a
;
PROBLEM: NonSolvent Resd 2  H   Uiso(max)/Uiso(min) Range        4.1 Ratio
RESPONSE: ...
;
_vrf_PLAT340_ToBr105_a
;
PROBLEM: Low Bond Precision on  C-C Bonds ..............    0.00515 Ang.
RESPONSE: ...
;
_vrf_PLAT907_ToBr105_a
;
PROBLEM: Flack x > 0.5, Structure Needs to be Inverted? .       1.70 Check
RESPONSE: ...
;
_vrf_PLAT910_ToBr105_a
;
PROBLEM: Missing # of FCF Reflection(s) Below Theta(Min).          9 Note
RESPONSE: ...
;
_vrf_PLAT911_ToBr105_a
;
PROBLEM: Missing FCF Refl Between Thmin & STh/L=    0.600          8 Report
RESPONSE: ...
;
_vrf_PLAT913_ToBr105_a
;
PROBLEM: Missing # of Very Strong Reflections in FCF ....          5 Note
RESPONSE: ...
;
# end Validation Reply Form
```

It is advisable to attempt to resolve as many as possible of the alerts in all categories. Often the minor alerts point to easily fixed oversights, errors and omissions in your CIF or refinement strategy, so attention to these fine details can be worthwhile. In order to resolve some of the more serious problems it may be necessary to carry out additional measurements or structure refinements. However, the purpose of your study may justify the reported deviations and the more serious of these should normally be commented upon in the discussion or experimental section of a paper or in the "special_details" fields of the CIF. checkCIF was carefully designed to identify outliers and unusual parameters, but every test has its limitations and alerts that are not important in a particular case may appear. Conversely, the absence of alerts does not guarantee there are no aspects of the results needing attention. It is up to the individual to critically assess their own results and, if necessary, seek expert advice.

Publication of your CIF in IUCr journals

A basic structural check has been run on your CIF. These basic checks will be run on all CIFs submitted for publication in IUCr journals (*Acta Crystallographica*, *Journal of Applied Crystallography*, *Journal of Synchrotron Radiation*); however, if you intend to submit to *Acta Crystallographica Section C* or *E* or *IUCrData*, you should make sure that full publication checks are run on the final version of your CIF prior to submission.

Publication of your CIF in other journals

Please refer to the *Notes for Authors* of the relevant journal for any special instructions relating to CIF submission.

PLATON version of 18/05/2022; check.def file version of 17/05/2022

Datablock ToBr105_a - ellipsoid plot

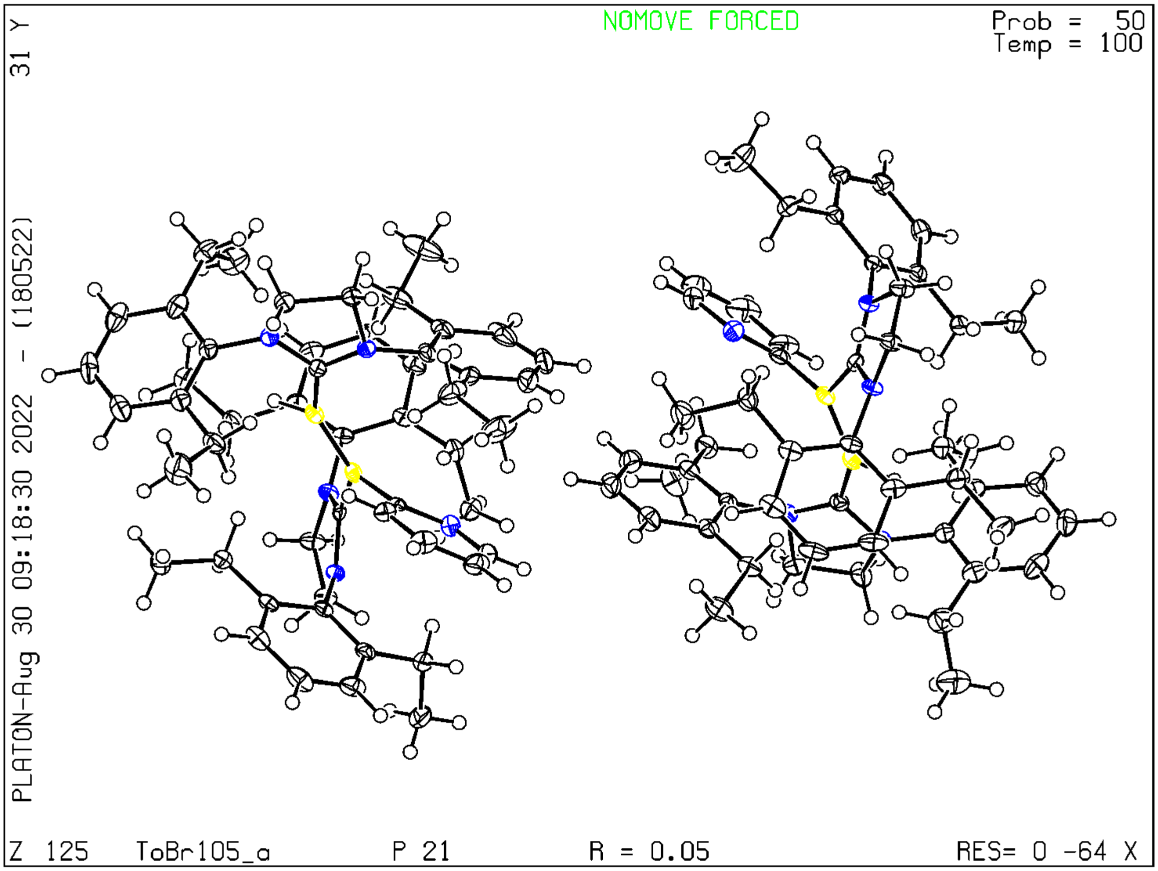

Supplement: Supplementary file 1 — Supporting Information [file ANIE-62-0-s003.pdf]
