# Supplementary material for: Metal‐Free Intermolecular C−H Borylation of N‐Heterocycles at B−B Multiple Bonds
Source: Angew Chem Int Ed Engl. 2022 Dec 22;62(5):e202213284. doi: 10.1002/anie.202213284 (PMC10107673; doi:10.1002/anie.202213284)

checkCIF/PLATON report

Structure factors have been supplied for datablock(s) BTR291_a

THIS REPORT IS FOR GUIDANCE ONLY. IF USED AS PART OF A REVIEW PROCEDURE FOR PUBLICATION, IT SHOULD NOT REPLACE THE EXPERTISE OF AN EXPERIENCED CRYSTALLOGRAPHIC REFEREE.

No syntax errors found.   CIF dictionary   Interpreting this report

Datablock: BTR291_a

```
Bond precision:   C-C = 0.0041 A          Wavelength=0.71073

Cell:             a=10.349(6)     b=16.275(7)          c=28.859(16)
                  alpha=90        beta=91.223(11)      gamma=90
Temperature:      100 K
```

	Calculated	Reported
Volume	4860(4)	4860(4)
Space group	P 21/c	P 21/c
Hall group	-P 2ybc	-P 2ybc
Moiety formula	C50 H72 B2 N4, C6 H6	C56 H78 B2 N4
Sum formula	C56 H78 B2 N4	C56 H78 B2 N4
Mr	828.84	828.84
Dx,g cm-3	1.133	1.133
Z	4	4
Mu (mm-1)	0.065	0.065
F000	1808.0	1808.0
F000'	1808.56	
h,k,lmax	12,20,35	12,20,35
Nref	9574	9525
Tmin,Tmax	0.987,0.994	0.621,0.746
Tmin'	0.978	

```
Correction method= # Reported T Limits: Tmin=0.621 Tmax=0.746
AbsCorr = MULTI-SCAN

Data completeness= 0.995            Theta(max)= 26.021

R(reflections)= 0.0635( 5163)                   wR2(reflections)= 0.1643( 9525)

S = 1.008                    Npar= 576
```

The following ALERTS were generated. Each ALERT has the format
test-name_ALERT_alert-type_alert-level.
Click on the hyperlinks for more details of the test.

Alert level C

```
RINTA01_ALERT_3_C  The value of Rint is greater than 0.12
            Rint given   0.123
PLAT042_ALERT_1_C Calc. and Reported MoietyFormula Strings  Differ     Please Check
PLAT340_ALERT_3_C Low Bond Precision on  C-C Bonds ..............    0.00412 Ang.
PLAT410_ALERT_2_C Short Intra H...H Contact  H1_3     ..H7_4     .       1.96 Ang.
                                                       x,y,z  =      1_555 Check
PLAT906_ALERT_3_C Large K Value in the Analysis of Variance ......      3.837 Check
PLAT910_ALERT_3_C Missing # of FCF Reflection(s) Below Theta(Min).          5 Note
PLAT911_ALERT_3_C Missing FCF Refl Between Thmin & STh/L=    0.600         37 Report
```

Alert level G

```
PLAT020_ALERT_3_G The Value of Rint is Greater Than 0.12 .........      0.123 Report
PLAT720_ALERT_4_G Number of Unusual/Non-Standard Labels ..........        117 Note
PLAT793_ALERT_4_G Model has Chirality at C6          (Centro SPGR)          R Verify
PLAT793_ALERT_4_G Model has Chirality at C1_3        (Centro SPGR)          R Verify
PLAT912_ALERT_4_G Missing # of FCF Reflections Above STh/L=  0.600          7 Note
PLAT913_ALERT_3_G Missing # of Very Strong Reflections in FCF ....          1 Note
PLAT941_ALERT_3_G Average HKL Measurement Multiplicity ..........         4.3 Low
PLAT965_ALERT_2_G The SHELXL WEIGHT Optimisation has not Converged     Please Check
PLAT978_ALERT_2_G Number C-C Bonds with Positive Residual Density.          2 Info
```

```
   0 ALERT level A = Most likely a serious problem - resolve or explain
   0 ALERT level B = A potentially serious problem, consider carefully
   7 ALERT level C = Check. Ensure it is not caused by an omission or oversight
   9 ALERT level G = General information/check it is not something unexpected

   1 ALERT type 1 CIF construction/syntax error, inconsistent or missing data
   3 ALERT type 2 Indicator that the structure model may be wrong or deficient
   8 ALERT type 3 Indicator that the structure quality may be low
   4 ALERT type 4 Improvement, methodology, query or suggestion
   0 ALERT type 5 Informative message, check
```

Validation response form

Please find below a validation response form (VRF) that can be filled in and pasted into your CIF.

```
# start Validation Reply Form
_vrf_RINTA01_BTR291_a
;
PROBLEM: The value of Rint is greater than 0.12
RESPONSE: ...
;
_vrf_PLAT042_BTR291_a
;
PROBLEM: Calc. and Reported MoietyFormula Strings  Differ     Please Check
RESPONSE: ...
```

```
;
_vrf_PLAT340_BTR291_a
;
PROBLEM: Low Bond Precision on  C-C Bonds ..............    0.00412 Ang.
RESPONSE: ...
;
_vrf_PLAT410_BTR291_a
;
PROBLEM: Short Intra H...H Contact  H1_3     ..H7_4     .       1.96 Ang.
RESPONSE: ...
;
_vrf_PLAT906_BTR291_a
;
PROBLEM: Large K Value in the Analysis of Variance ......      3.837 Check
RESPONSE: ...
;
_vrf_PLAT910_BTR291_a
;
PROBLEM: Missing # of FCF Reflection(s) Below Theta(Min).          5 Note
RESPONSE: ...
;
_vrf_PLAT911_BTR291_a
;
PROBLEM: Missing FCF Refl Between Thmin & STh/L=    0.600         37 Report
RESPONSE: ...
;
# end Validation Reply Form
```

It is advisable to attempt to resolve as many as possible of the alerts in all categories. Often the minor alerts point to easily fixed oversights, errors and omissions in your CIF or refinement strategy, so attention to these fine details can be worthwhile. In order to resolve some of the more serious problems it may be necessary to carry out additional measurements or structure refinements. However, the purpose of your study may justify the reported deviations and the more serious of these should normally be commented upon in the discussion or experimental section of a paper or in the "special_details" fields of the CIF. checkCIF was carefully designed to identify outliers and unusual parameters, but every test has its limitations and alerts that are not important in a particular case may appear. Conversely, the absence of alerts does not guarantee there are no aspects of the results needing attention. It is up to the individual to critically assess their own results and, if necessary, seek expert advice.

Publication of your CIF in IUCr journals

A basic structural check has been run on your CIF. These basic checks will be run on all CIFs submitted for publication in IUCr journals (*Acta Crystallographica*, *Journal of Applied Crystallography*, *Journal of Synchrotron Radiation*); however, if you intend to submit to *Acta Crystallographica Section C* or *E* or *IUCrData*, you should make sure that full publication checks are run on the final version of your CIF prior to submission.

Publication of your CIF in other journals

Please refer to the *Notes for Authors* of the relevant journal for any special instructions relating to CIF submission.

PLATON version of 18/05/2022; check.def file version of 17/05/2022

Datablock BTR291_a - ellipsoid plot

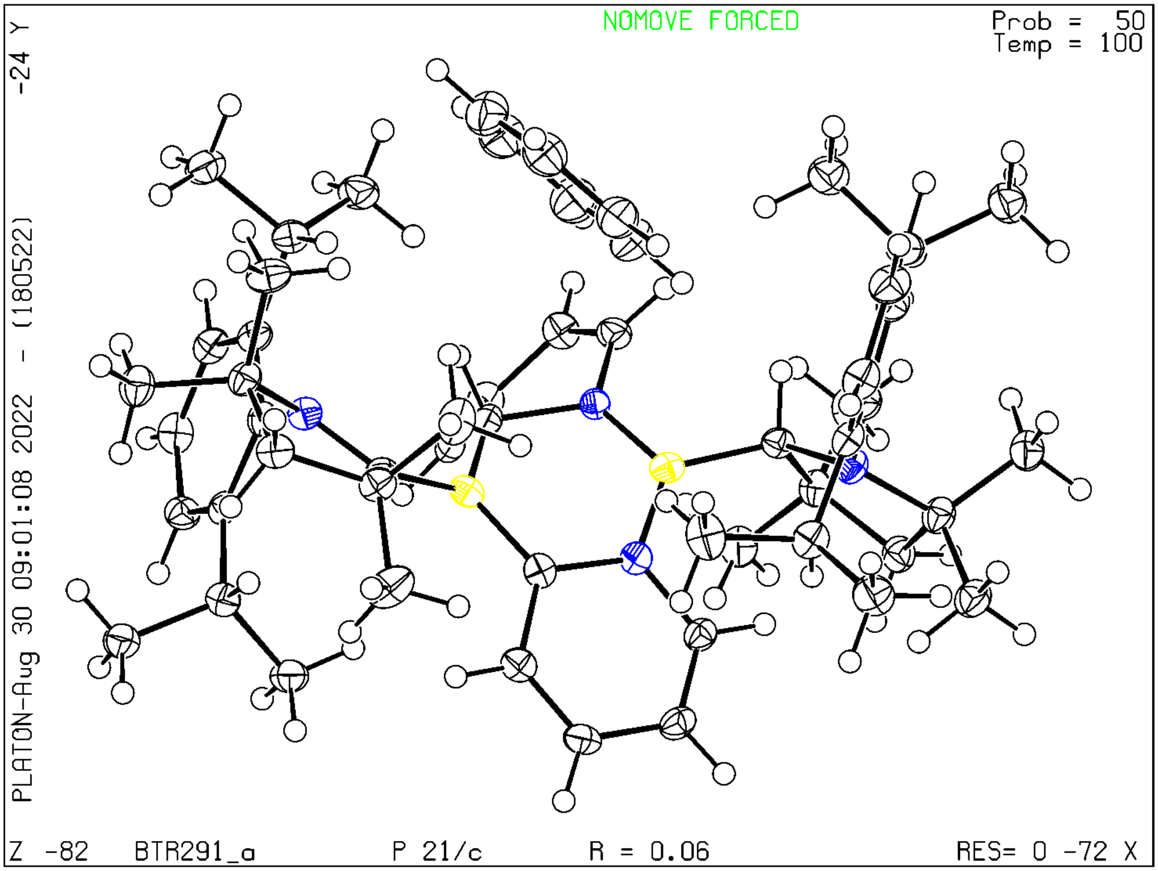

Supplement: Supplementary file 2 — Supporting Information [file ANIE-62-0-s001.pdf]
