# Supplementary material for: Metal‐Free Intermolecular C−H Borylation of N‐Heterocycles at B−B Multiple Bonds
Source: Angew Chem Int Ed Engl. 2022 Dec 22;62(5):e202213284. doi: 10.1002/anie.202213284 (PMC10107673; doi:10.1002/anie.202213284)

checkCIF/PLATON report

Structure factors have been supplied for datablock(s) BTR348Benz_a

THIS REPORT IS FOR GUIDANCE ONLY. IF USED AS PART OF A REVIEW PROCEDURE FOR PUBLICATION, IT SHOULD NOT REPLACE THE EXPERTISE OF AN EXPERIENCED CRYSTALLOGRAPHIC REFEREE.

No syntax errors found. CIF dictionary Interpreting this report

Datablock: BTR348Benz_a

```
Bond precision:   C-C = 0.0045 A           Wavelength=0.71073

Cell:               a=18.70(3)      b=10.573(16)        c=47.37(8)
                    alpha=90        beta=90.33(3)       gamma=90
Temperature:        100 K
```

	Calculated	Reported
Volume	9366(26)	9367(26)
Space group	I 2/a	I 2/a
Hall group	-I 2ya	-I 2ya
Moiety formula	C50 H72 B2 N4, 0.5(C6 H6)	C53 H75 B2 N4
Sum formula	C53 H75 B2 N4	C53 H75 B2 N4
Mr	789.79	789.79
Dx,g cm-3	1.120	1.120
Z	8	8
Mu (mm-1)	0.064	0.064
F000	3448.0	3448.0
F000'	3449.05	
h,k,lmax	22,12,56	22,12,56
Nref	8279	8282
Tmin,Tmax	0.982,0.983	0.496,0.726
Tmin'	0.982	

```
Correction method= # Reported T Limits: Tmin=0.496 Tmax=0.726
AbsCorr = MULTI-SCAN

Data completeness= 1.000            Theta(max)= 25.023

R(reflections)= 0.0631( 5050)             wR2(reflections)= 0.1769( 8282)

S = 1.033                   Npar= 676
```

The following ALERTS were generated. Each ALERT has the format
test-name_ALERT_alert-type_alert-level.
Click on the hyperlinks for more details of the test.

Alert level C

```
RINTA01_ALERT_3_C  The value of Rint is greater than 0.12
            Rint given   0.157
PLAT042_ALERT_1_C Calc. and Reported MoietyFormula Strings  Differ     Please Check
PLAT148_ALERT_3_C s.u. on the      a    - Axis is (Too) Large ....      0.030 Ang.
PLAT148_ALERT_3_C s.u. on the      b    - Axis is (Too) Large ....     0.0160 Ang.
PLAT148_ALERT_3_C s.u. on the      c    - Axis is (Too) Large ....      0.080 Ang.
PLAT340_ALERT_3_C Low Bond Precision on  C-C Bonds ..............    0.00454 Ang.
PLAT410_ALERT_2_C Short Intra H...H Contact  H1_3     ..H8_4     .       1.93 Ang.
                                                      x,y,z  =      1_555 Check
```

Alert level G

```
PLAT003_ALERT_2_G Number of Uiso or Uij Restrained non-H Atoms ...         28 Report
PLAT020_ALERT_3_G The Value of Rint is Greater Than 0.12 .........      0.157 Report
PLAT178_ALERT_4_G The CIF-Embedded .res File Contains SIMU Records          1 Report
PLAT187_ALERT_4_G The CIF-Embedded .res File Contains RIGU Records          1 Report
PLAT301_ALERT_3_G Main Residue  Disorder .............(Resd  1  )        25% Note
PLAT367_ALERT_2_G Long?  C(sp?)-C(sp?) Bond  C1_1     - C4_1     .       1.57 Ang.
PLAT367_ALERT_2_G Long?  C(sp?)-C(sp?) Bond  C1_3     - C4_3     .       1.58 Ang.
PLAT410_ALERT_2_G Short Intra H...H Contact  H1_11    ..H3AB_3   .       2.11 Ang.
                                                      x,y,z  =      1_555 Check
PLAT410_ALERT_2_G Short Intra H...H Contact  H4_11    ..H1_1     .       2.13 Ang.
                                                      x,y,z  =      1_555 Check
PLAT410_ALERT_2_G Short Intra H...H Contact  H10_11   ..H1_3     .       2.12 Ang.
                                                      x,y,z  =      1_555 Check
PLAT410_ALERT_2_G Short Intra H...H Contact  H1_1     ..H4_111   .       1.99 Ang.
                                                      x,y,z  =      1_555 Check
PLAT410_ALERT_2_G Short Intra H...H Contact  H3AB_1   ..H7_111   .       2.03 Ang.
                                                      x,y,z  =      1_555 Check
PLAT720_ALERT_4_G Number of Unusual/Non-Standard Labels ..........        156 Note
PLAT802_ALERT_4_G CIF Input Record(s) with more than 80 Characters          2 Info
PLAT860_ALERT_3_G Number of Least-Squares Restraints ............         696 Note
PLAT909_ALERT_3_G Percentage of I>2sig(I) Data at Theta(Max) Still        31% Note
PLAT978_ALERT_2_G Number C-C Bonds with Positive Residual Density.          2 Info
```

0 **ALERT level A** = Most likely a serious problem - resolve or explain
0 **ALERT level B** = A potentially serious problem, consider carefully
7 **ALERT level C** = Check. Ensure it is not caused by an omission or oversight
17 **ALERT level G** = General information/check it is not something unexpected

1 ALERT type 1 CIF construction/syntax error, inconsistent or missing data
10 ALERT type 2 Indicator that the structure model may be wrong or deficient
9 ALERT type 3 Indicator that the structure quality may be low
4 ALERT type 4 Improvement, methodology, query or suggestion
0 ALERT type 5 Informative message, check

Validation response form

Please find below a validation response form (VRF) that can be filled in and pasted into your CIF.

```
# start Validation Reply Form
_vrf_RINTA01_BTR348Benz_a
;
PROBLEM: The value of Rint is greater than 0.12
RESPONSE: ...
;
_vrf_PLAT042_BTR348Benz_a
;
PROBLEM: Calc. and Reported MoietyFormula Strings  Differ     Please Check
RESPONSE: ...
;
_vrf_PLAT148_BTR348Benz_a
;
PROBLEM: s.u. on the      a    - Axis is (Too) Large ....      0.030 Ang.
RESPONSE: ...
;
_vrf_PLAT340_BTR348Benz_a
;
PROBLEM: Low Bond Precision on  C-C Bonds ..............    0.00454 Ang.
RESPONSE: ...
;
_vrf_PLAT410_BTR348Benz_a
;
PROBLEM: Short Intra H...H Contact  H1_3     ..H8_4     .       1.93 Ang.
RESPONSE: ...
;
# end Validation Reply Form
```

It is advisable to attempt to resolve as many as possible of the alerts in all categories. Often the minor alerts point to easily fixed oversights, errors and omissions in your CIF or refinement strategy, so attention to these fine details can be worthwhile. In order to resolve some of the more serious problems it may be necessary to carry out additional measurements or structure refinements. However, the purpose of your study may justify the reported deviations and the more serious of these should normally be commented upon in the discussion or experimental section of a paper or in the "special_details" fields of the CIF. checkCIF was carefully designed to identify outliers and unusual parameters, but every test has its limitations and alerts that are not important in a particular case may appear. Conversely, the absence of alerts does not guarantee there are no aspects of the results needing attention. It is up to the individual to critically assess their own results and, if necessary, seek expert advice.

Publication of your CIF in IUCr journals

A basic structural check has been run on your CIF. These basic checks will be run on all CIFs submitted for publication in IUCr journals (*Acta Crystallographica*, *Journal of Applied Crystallography*, *Journal of Synchrotron Radiation*); however, if you intend to submit to *Acta Crystallographica Section C* or *E* or *IUCrData*, you should make sure that full publication checks are run on the final version of your CIF prior to submission.

Publication of your CIF in other journals

Please refer to the *Notes for Authors* of the relevant journal for any special instructions relating to CIF submission.

PLATON version of 18/05/2022; check.def file version of 17/05/2022

Datablock BTR348Benz_a - ellipsoid plot

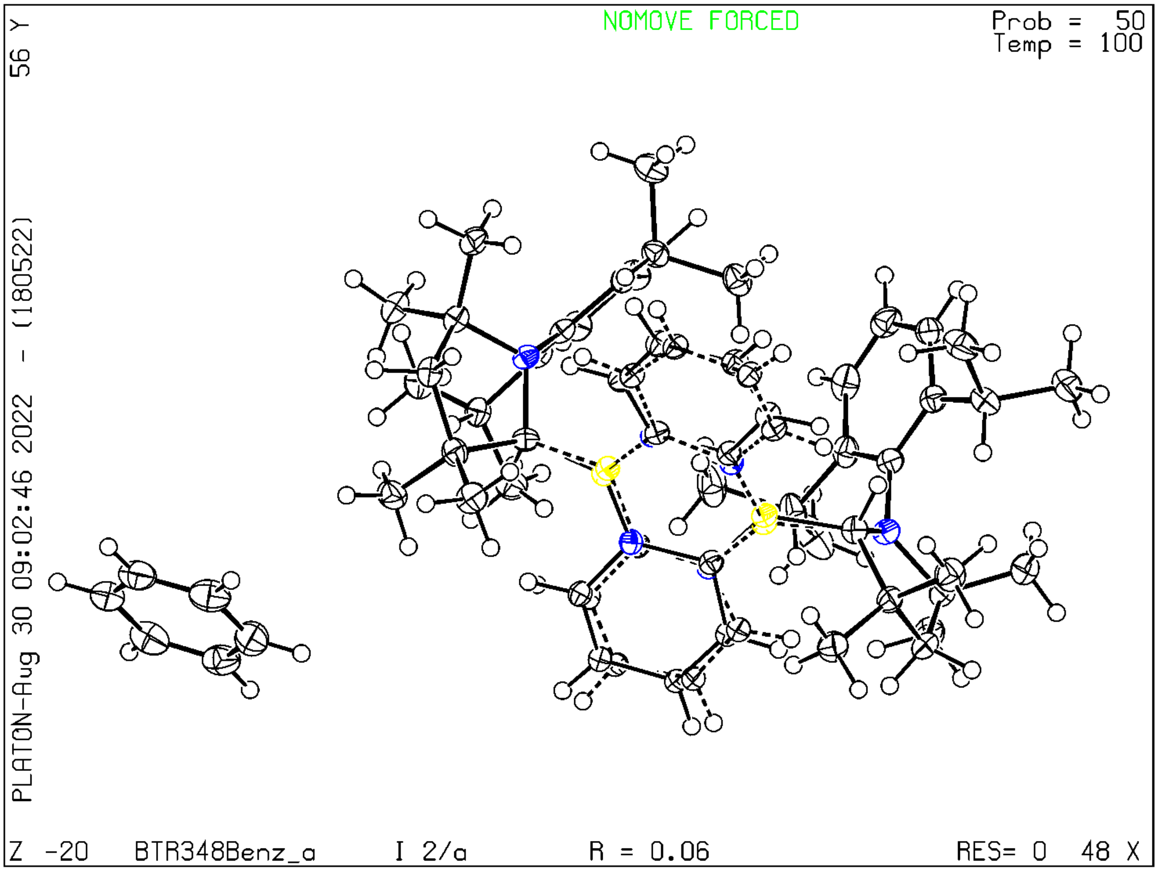

Supplement: Supplementary file 3 — Supporting Information [file ANIE-62-0-s005.pdf]
