# Supplementary material for: Metal‐Free Intermolecular C−H Borylation of N‐Heterocycles at B−B Multiple Bonds
Source: Angew Chem Int Ed Engl. 2022 Dec 22;62(5):e202213284. doi: 10.1002/anie.202213284 (PMC10107673; doi:10.1002/anie.202213284)

# checkCIF/PLATON report

Structure factors have been supplied for datablock(s) ToBr112_a

THIS REPORT IS FOR GUIDANCE ONLY. IF USED AS PART OF A REVIEW PROCEDURE FOR PUBLICATION, IT SHOULD NOT REPLACE THE EXPERTISE OF AN EXPERIENCED CRYSTALLOGRAPHIC REFEREE.

No syntax errors found. CIF dictionary Interpreting this report

## Datablock: ToBr112_a

---

```
Bond precision:   C-C = 0.0027 A          Wavelength=0.71073

Cell:              a=22.6082(5)     b=10.9231(2)        c=20.1980(5)
                   alpha=90         beta=110.968(1)     gamma=90
Temperature:       100 K

                  Calculated                         Reported
Volume            4657.63(18)                        4657.63(18)
Space group       P 21/c                             P 21/c
Hall group        -P 2ybc                            -P 2ybc
Moiety formula    C55 H67 B2 N5                      C55 H67 B2 N5
Sum formula       C55 H67 B2 N5                      C55 H67 B2 N5
Mr                819.76                             819.75
Dx,g cm-3         1.169                              1.169
Z                 4                                  4
Mu (mm-1)         0.067                              0.067
F000              1768.0                             1768.0
F000'             1768.55
h,k,lmax          27,13,24                           27,13,24
Nref              9181                               9172
Tmin,Tmax         0.980,0.985                        0.701,0.745
Tmin'             0.979

Correction method= # Reported T Limits: Tmin=0.701 Tmax=0.745
AbsCorr = MULTI-SCAN

Data completeness= 0.999           Theta(max)= 26.022

R(reflections)= 0.0471( 7160)                             wR2(reflections)=
                                                          0.1197( 9172)
S = 1.028                    Npar= 671
```

The following ALERTS were generated. Each ALERT has the format
**test-name_ALERT_alert-type_alert-level.**
Click on the hyperlinks for more details of the test.

### Alert level C

```
PLAT220_ALERT_2_C NonSolvent   Resd 1  C   Ueq(max)/Ueq(min) Range        4.1 Ratio
PLAT222_ALERT_3_C NonSolvent Resd 1  H   Uiso(max)/Uiso(min) Range        5.4 Ratio
PLAT230_ALERT_2_C Hirshfeld Test Diff for    C16      --C17      .        6.6 s.u.
PLAT410_ALERT_2_C Short Intra H...H Contact  H11B     ..H21A     .       1.97 Ang.
                                                      x,y,z  =      1_555 Check
PLAT906_ALERT_3_C Large K Value in the Analysis of Variance ......      2.359 Check
PLAT911_ALERT_3_C Missing FCF Refl Between Thmin & STh/L=    0.600          3 Report
```

### Alert level G

```
PLAT002_ALERT_2_G Number of Distance or Angle Restraints on AtSite          3 Note
PLAT003_ALERT_2_G Number of Uiso or Uij Restrained non-H Atoms ...         22 Report
PLAT093_ALERT_1_G No s.u.'s on H-positions, Refinement Reported as      mixed Check
PLAT176_ALERT_4_G The CIF-Embedded .res File Contains SADI Records          1 Report
PLAT178_ALERT_4_G The CIF-Embedded .res File Contains SIMU Records          2 Report
PLAT187_ALERT_4_G The CIF-Embedded .res File Contains RIGU Records          2 Report
PLAT301_ALERT_3_G Main Residue  Disorder .............(Resd  1  )        18% Note
PLAT410_ALERT_2_G Short Intra H...H Contact  H11A     ..H1A_21   .       1.95 Ang.
                                                      x,y,z  =      1_555 Check
PLAT410_ALERT_2_G Short Intra H...H Contact  H11A     ..H1AB_2   .       2.13 Ang.
                                                      x,y,z  =      1_555 Check
PLAT410_ALERT_2_G Short Intra H...H Contact  H11B     ..H1A_2    .       2.13 Ang.
                                                      x,y,z  =      1_555 Check
PLAT413_ALERT_2_G Short Inter XH3 .. XHn     H45A     ..H4_11    .       2.10 Ang.
                                                 2-x,1-y,1-z  =     3_766 Check
PLAT720_ALERT_4_G Number of Unusual/Non-Standard Labels .........          52 Note
PLAT860_ALERT_3_G Number of Least-Squares Restraints ............         439 Note
PLAT910_ALERT_3_G Missing # of FCF Reflection(s) Below Theta(Min).          4 Note
PLAT912_ALERT_4_G Missing # of FCF Reflections Above STh/L=  0.600          4 Note
PLAT933_ALERT_2_G Number of HKL-OMIT Records in Embedded .res File          1 Note
PLAT978_ALERT_2_G Number C-C Bonds with Positive Residual Density.          7 Info
```

0 **ALERT level A** = Most likely a serious problem - resolve or explain
0 **ALERT level B** = A potentially serious problem, consider carefully
6 **ALERT level C** = Check. Ensure it is not caused by an omission or oversight
17 **ALERT level G** = General information/check it is not something unexpected

1 ALERT type 1 CIF construction/syntax error, inconsistent or missing data
11 ALERT type 2 Indicator that the structure model may be wrong or deficient
6 ALERT type 3 Indicator that the structure quality may be low
5 ALERT type 4 Improvement, methodology, query or suggestion
0 ALERT type 5 Informative message, check

## Validation response form

Please find below a validation response form (VRF) that can be filled in and pasted into your CIF.

```
# start Validation Reply Form
_vrf_PLAT220_ToBr112_a
;
PROBLEM: NonSolvent   Resd 1  C   Ueq(max)/Ueq(min) Range        4.1 Ratio
RESPONSE: ...
;
_vrf_PLAT222_ToBr112_a
;
PROBLEM: NonSolvent Resd 1  H   Uiso(max)/Uiso(min) Range        5.4 Ratio
RESPONSE: ...
;
_vrf_PLAT230_ToBr112_a
;
PROBLEM: Hirshfeld Test Diff for    C16      --C17      .        6.6 s.u.
RESPONSE: ...
;
_vrf_PLAT410_ToBr112_a
;
PROBLEM: Short Intra H...H Contact  H11B     ..H21A     .       1.97 Ang.
RESPONSE: ...
;
_vrf_PLAT906_ToBr112_a
;
PROBLEM: Large K Value in the Analysis of Variance ......      2.359 Check
RESPONSE: ...
;
_vrf_PLAT911_ToBr112_a
;
PROBLEM: Missing FCF Refl Between Thmin & STh/L=    0.600          3 Report
RESPONSE: ...
;
# end Validation Reply Form
```

It is advisable to attempt to resolve as many as possible of the alerts in all categories. Often the minor alerts point to easily fixed oversights, errors and omissions in your CIF or refinement strategy, so attention to these fine details can be worthwhile. In order to resolve some of the more serious problems it may be necessary to carry out additional measurements or structure refinements. However, the purpose of your study may justify the reported deviations and the more serious of these should normally be commented upon in the discussion or experimental section of a paper or in the "special_details" fields of the CIF. checkCIF was carefully designed to identify outliers and unusual parameters, but every test has its limitations and alerts that are not important in a particular case may appear. Conversely, the absence of alerts does not guarantee there are no aspects of the results needing attention. It is up to the individual to critically assess their own results and, if necessary, seek expert advice.

**Publication of your CIF in IUCr journals**

A basic structural check has been run on your CIF. These basic checks will be run on all CIFs submitted for publication in IUCr journals (*Acta Crystallographica*, *Journal of Applied Crystallography*, *Journal of Synchrotron Radiation*); however, if you intend to submit to *Acta Crystallographica Section C* or *E* or *IUCrData*, you should make sure that full publication checks are run on the final version of your CIF prior to submission.

**Publication of your CIF in other journals**

Please refer to the *Notes for Authors* of the relevant journal for any special instructions relating to CIF submission.

---

**PLATON version of 18/05/2022; check.def file version of 17/05/2022**

**Datablock ToBr112_a** - ellipsoid plot

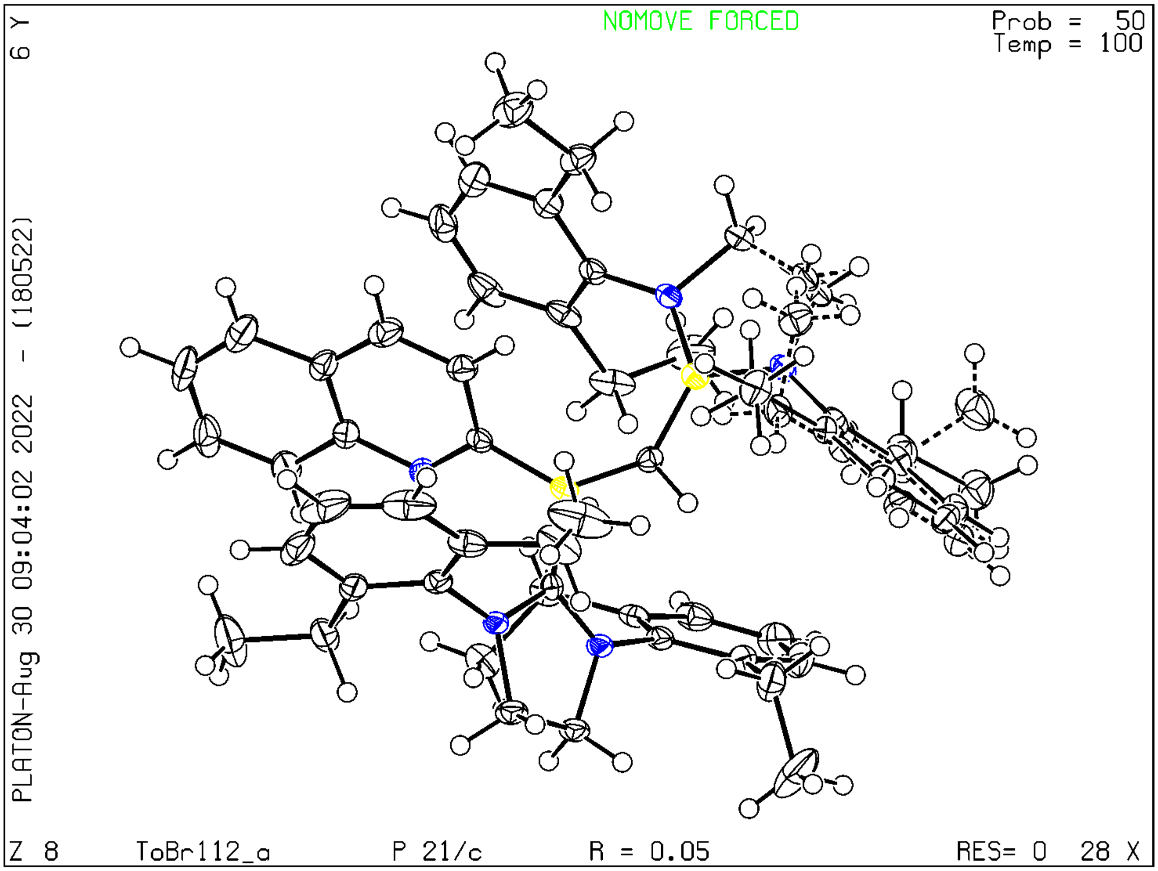

Supplement: Supplementary file 4 — Supporting Information [file ANIE-62-0-s008.pdf]
