# Supplementary material for: Metal‐Free Intermolecular C−H Borylation of N‐Heterocycles at B−B Multiple Bonds
Source: Angew Chem Int Ed Engl. 2022 Dec 22;62(5):e202213284. doi: 10.1002/anie.202213284 (PMC10107673; doi:10.1002/anie.202213284)
Supplement: Supplementary file 5 — Supporting Information [file ANIE-62-0-s002.pdf]

Angewandte Chemie
Eine Zeitschrift der Gesellschaft Deutscher Chemiker

Supporting Information

Metal-Free Intermolecular C–H Borylation of *N*-Heterocycles at B–B Multiple Bonds

*T. Brückner, B. Ritschel, J. O. C. Jiménez-Halla, F. Fantuzzi, D. Duwe, C. Markl, R. D. Dewhurst, M. Dietz, H. Braunschweig**

Experimental Details

General experimental considerations: All syntheses were carried out in argon-filled gloveboxes or with standard Schlenk techniques.[1] All solvents were dried by distillation over appropriate drying agents[2] and stored over molecular sieves. All solution NMR spectra were acquired on a Bruker Avance 400 NMR spectrometer (^{1}H: 400.1 MHz, ^{13}C: 101 MHz, ^{11}B: 128.5 MHz) or a Bruker Avance I 500 spectrometer (^{1}H: 500.1 MHz, ^{13}C: 125.8 MHz, ^{11}B: 160.5 MHz). ^{1}H and ^{13}C NMR spectra were referenced to external TMS via residual protons of the solvent (^{1}H) or the solvent itself (^{13}C). ^{11}B NMR spectra were referenced to external $BF_3(OEt_2)$. High-resolution mass spectrometry data was obtained using a Thermo Scientific Exactive Plus spectrometer in ASAP or LIFDI mode. Pyridine and quinoline were purchased from Sigma-Aldrich or abcr and used without further purification. $B_2(SIDep)_2$[3] and $B_2(CAAC)_2$[4] were synthesized following literature procedures.

Preparation of 3: A solution of 20 mg (30 µmol) B_2SIDep_2 (**1**) in 0.6 mL C_6D_6 was treated with 2.3 mg (1.0 equiv) pyridine, resulting in an immediate color change from red to blue. ^{11}B NMR spectroscopic monitoring after 10 min showed complete consumption of the diboryne and new signals at 35 and 25 ppm. After removal of all volatiles the blue residue was washed with hexane (3 x 0.6 mL), dried in vacuo and **3** was isolated in 82% yield as a blue solid. Single crystals suitable for X-ray diffraction were obtained by slow evaporation of a saturated hexane solution of **3**.

^{1}H{^{11}B} NMR (500.1 MHz, C_6D_6): δ = 8.14 – 8.13 (m, 1H, C$H_{Ar,2\text{-}pyr}$), 7.15 – 7.06 (m, 4H, C$H_{Ar,p\text{-}Dep}$), 6.99 – 6.95 (m, 8H, C$H_{Ar,m\text{-}Dep}$), 6.61 – 6.58 (m, 1*H*, CH$_{Ar,4\text{-}pyr}$), 6.39 – 6.38 (m, 1H, C$H_{Ar,3\text{-}pyr}$), 6.32 – 6.29 (m 1H, C$H_{Ar,5\text{-}pyr}$), 3.35 (br. s, 1H, B*H*), 3.23 – 3.22 (two overlapping s, 8H, NCH_2), 2.79 – 2.71 (m, 4H, CH_{2Et}), 2.68 – 2.60 (m, 4H, CH_{2Et}), 2.47 – 2.38 (m, 8H, CH_{2Et}), 1.22 – 1.18 (m, 24H, CH_{3Et}) ppm.

^{13}C{^{1}H} NMR (125.8 MHz, C_6D_6): δ = 188.7 ($C_{Carbene}$, detected by HMBC), 187.4 ($C_{Carbene}$, detected by HMBC), 180.3 (B*C*=N), 147.3 (CH$_{Ar,2\text{-}pyr}$), 142.0 (C_q), 141.9 (C_q), 140.1 (C_q), 139.5 (C_q), 130.0 (CH$_{Ar,4\text{-}pyr}$), 129.5 (CH$_{Ar,3\text{-}pyr}$), 127.2 (CH$_{Ar}$), 126.7 (CH$_{Ar}$), 125.8 (CH$_{Ar}$), 125.6 (CH$_{Ar}$), 115.5 (CH$_{Ar,5\text{-}pyr}$), 51.0 (NCH$_2$), 50.9 (NCH$_2$), 24.6 (CH$_{2Et}$), 24.5 (CH$_{2Et}$), 14.6 (CH$_{2Et}$), 14.1 (CH$_{3Et}$) ppm.

^{11}B{^{1}H} NMR (160.5 MHz, C_6D_6): δ = 34.2 (NC*B*), 25.1 (*B*H) ppm.

^{15}N NMR (HMBC, 50.7 MHz, C_6D_6): δ = −52.8 (N_{pyr}), −265.8 (N_{NHC}), −266.7 ppm.

HRMS (LIFDI): m/z ($C_{51}H_{65}B_2N_5$) = calc.: 769.5421, found: 769.5407.

Preparation of 4: 40 mg (67.5 µmol) B_2CAAC_2 (**2**) were dissolved in 0.6 mL benzene and the mixture was treated with 11.8 mg (149 µmol) pyridine. After one hour the ^{11}B NMR spectrum showed complete consumption of the starting material and new signals at 32 and 22 ppm, while the solution turned from purple to pink. After evaporation of all volatiles, the pink residue was recrystallized from pentane. The product was isolated in 82% yield as a purple solid. Single crystals suitable for X-ray diffraction were obtained by slow evaporation of a saturated hexane solution of **4**.

^{1}H NMR (278K, 400.6 MHz, C_6D_6) δ = 7.86 (br d, 1H, $^3J_{HH}$ = 6.9 Hz, C_6H_4N-N–C*H*), 7.33 (br d, 1H, $^3J_{HH}$ = 8.5 Hz, C_6H_4N-Cq–C*H*), 7.27 (dd, 1H, $^3J_{HH}$ = 7.6 Hz, $^4J_{HH}$ = 1.9 Hz, *m*-CH_{Ar}), 7.19 (dd, 1H, $^3J_{HH}$ = 7.6 Hz, $^3J_{HH}$ = 7.6 Hz, *p*-CH_{Ar}), 7.16–7.14 (m, 2H, C_6H_5N-N–C*H*, C_6H_5N-N–CH–C*H*), 7.11 (dd, 1H, $^3J_{HH}$ = 7.5 Hz, $^3J_{HH}$ = 7.5 Hz, *p*-CH_{Ar}), 7.06 (dd, 1H, $^3J_{HH}$ = 7.5 Hz, $^4J_{HH}$ = 2.1 Hz, *m*-CH_{Ar}), 7.02 (dd, 1H, $^3J_{HH}$ = 7.6 Hz, $^4J_{HH}$ = 1.9 Hz, *m*-CH_{Ar}), 6.99 (dd, 1H, $^3J_{HH}$ = 7.5 Hz, $^4J_{HH}$ = 2.1 Hz, *m*-CH_{Ar}), 5.89 (br dd, 1H, $^3J_{HH}$ = 8.5 Hz, $^3J_{HH}$ = 6.5 Hz, C_6H_4N-Cq–CH–C*H*), 5.66–5.59 (m, 2H, C_6H_5N-N–C*H*, C_6H_5N-NCH–C*H*), 5.54 (m, 1H, C_6H_4N-N–CH–C*H*), 4.26 (s, 1H, cAAC-C*H*), 4.16 (sept, 1H, $^3J_{HH}$ = 6.8 Hz, C*H*(CH_3)$_2$), 3.62 (sept, 1H, $^3J_{HH}$ = 6.7 Hz, C*H*(CH_3)$_2$), 3.48 (sept, 1H, $^3J_{HH}$ = 6.7 Hz, C*H*(CH_3)$_2$), 3.33 (br s, 1H, C_6H_4N-B–C*H*), 3.06 (sept, 1H, $^3J_{HH}$ = 6.6 Hz, -C*H*(CH_3)$_2$), 1.98 (d, 1H, $^2J_{HH}$ = 13.4 Hz, Cq–CH_2–Cq), 1.76 (d, 1H, $^2J_{HH}$ = 12.2 Hz, Cq–CH_2–Cq), 1.75 (d, 1H, $^2J_{HH}$ = 13.4 Hz, Cq–CH_2–Cq), 1.69 (s, 3H, Cq(CH_3)$_2$–CH_2), 1.55 (s, 3H, N–Cq(CH_3)$_2$), 1.54 (d, 1H, $^2J_{HH}$ = 12.2 Hz, Cq–CH_2–Cq), 1.39 (d, 3H, $^3J_{HH}$ = 6.7 Hz, CH(CH_3)$_2$), 1.37 (d, 6H, $^3J_{HH}$ = 6.8 Hz, CH(CH_3)$_2$), 1.34 (s, 3H, Cq(CH_3)$_2$–CH_2), 1.26 (s, 3H, N–Cq(CH_3)$_2$), 1.25 (d, 3H, $^3J_{HH}$ = 6.6 Hz, CH(CH_3)$_2$, 1.24 (d, 3H, $^3J_{HH}$ = 6.7 Hz, CH(CH_3)$_2$, 1.23 (d, 3H, $^3J_{HH}$ = 6.7 Hz, CH(CH_3)$_2$, 1.19 (s, 3H, Cq(CH_3)$_2$–CH_2), 1.16 (d, 3H, $^3J_{HH}$ = 6.7 Hz, CH(CH_3)$_2$), 1.15 (d, 3H, $^3J_{HH}$ = 6.6 Hz, CH(CH_3)$_2$),1.11 (s, 3H, Cq(CH_3)$_2$–CH_2), 0.96 (s, 3H, N–Cq(CH_3)$_2$), 0.77 (s, 3H, N–Cq(CH_3)$_2$) ppm.

^{13}C{^{1}H} NMR (125.8 MHz, 298 K, C_6D_6): δ = 184.2 (carbene-C_q), 152.3 (*o*-Dipp-C_q), 150.6 (*o*-Dipp-C_q), 147.8 (*o*-Dipp-C_q), 146.2 (*o*-Dipp-C_q), 143.1 (*i*-Dipp-C_q), 141.0 (C_5H_4N-N–*C*H), 140.0 (*i*-Dipp-C_q), 138.8 (C_5H_4N-C_q–*C*H), 132.5 (C_5H_5N-N–*C*H, C_5H_5N-NCH–*C*H), 128.5 (C_5H_4N-C_q), 127.0 (*p*-Dipp-*C*H), 126.8 (*p*-Dipp-*C*H), 125.9 (*m*-Dipp-*C*H), 125.7 (*m*-Dipp-C_q), 125.3 (*m*-Dipp-*C*H), 125.1 (*m*-Dipp-*C*H), 121.7 (C_5H_4N-C_qCH–*C*H), 119.8 (C_5H_5N-(CH)$_2$–*C*H) 115.1 (C_5H_5N-CH–*C*H), 109.0 (C_5H_4N-NCH–*C*H), 65.7 (N–C_q(CH_3)$_2$), 65.2 (C_5H_5N-B–*C*H, N–C_q(CH_3)$_2$), 59.2 (C_q(CH_3)$_2$–*C*H_2–C_q(CH)$_3$), 57.5 (C_q(CH_3)$_2$–*C*H_2–C_q(CH)$_3$), 48.2 (cAAC-*C*H, C_q(CH_3)$_2$–CH_2), 41.0 (C_q(CH_3)$_2$–CH_2), 38.1 (C_q(*C*H_3)$_2$–CH_2), 35.6 (C_q(*C*H_3)$_2$–CH_2), 33.4 (C_q(*C*H_3)$_2$–CH_2), 32.0 (N–C_q(*C*H_3)$_2$), 30.8 (N–C_q(*C*H_3)$_2$), 29.1 (N–C_q(*C*H_3)$_2$), 29.0 (C_q(*C*H_3)$_2$–CH_2, Dipp-*C*H(CH_3)$_2$), 28.89 (Dipp-*C*H(CH_3)$_2$), 28.85 (Dipp-*C*H(CH_3)$_2$), 28.8 (Dipp-*C*H(CH_3)$_2$), 28.1 (N–C_q(*C*H_3)$_2$), 28.0 (Dipp-CH(*C*H_3)$_2$), 26.3 (Dipp-CH(*C*H_3)$_2$), 25.1 (Dipp-CH(*C*H_3)$_2$), 24.9 (Dipp-CH(*C*H_3)$_2$), 24.4 (Dipp-CH(*C*H_3)$_2$), 24.3 (Dipp-CH(*C*H_3)$_2$) ppm.

^{11}B NMR (298 K, 128.5 MHz, C_6D_6): δ = 32.1 (br s), 22.5 (s) ppm.

HRMS (LIFDI): calcd. [$C_{50}H_{72}B_2N_4$ + H^+]$^+$, m/z = 751.6016; found: m/z = 751.6002.

Preparation of 5: 40 mg (67.5 µmol) B_2CAAC_2 (**2**) were dissolved in 0.6 mL benzene and the solution was treated with 11.8 mg (149 µmol) pyridine. This mixture was heated at 80 °C for five minutes, leading to full conversion to **4** and after and additional 14 hours at 80 ºC the ^{11}B NMR spectrum showed a new, broad signal at 28 ppm, while the solution turned dark pink. After evaporation of all volatiles, the residue was recrystallized from pentane. The product was isolated in 99% yield as a purple solid. Single crystals suitable for X-ray diffraction were obtained by slow evaporation of a saturated hexane or benzene solution of **5**.

^{1}H NMR (298 K, 500.1 MHz, C_6D_6) δ = 9.02 (d, 1H, $^3J_{HH}$ = 8.7 Hz, C_6H_4N-Cq–C*H*), 9.00 (d, 1H, $^3J_{HH}$ = 7.4 Hz, C_6H_4N-N–C*H*), 8.29 (d, 1H, $^3J_{HH}$ = 8.8 Hz, C_6H_4N-Cq–C*H*), 8.11 (d, 1H, $^3J_{HH}$ = 7.4 Hz, C_6H_4N-N–C*H*),

7.06–7.03 (m, 2H, *m*-C*H*$_{Ar}$, *p*-C*H*$_{Ar}$), 7.02 (dd, 1H, $^3J_{HH}$ = 7.6 Hz, $^3J_{HH}$ = 7.6 Hz, *p*-C*H*$_{Ar}$), 6.95 (dd, 1H, $^3J_{HH}$ = 7.6 Hz, $^4J_{HH}$ = 1.1 Hz, *m*-C*H*$_{Ar}$), 6.77–6.74 (m, 2H, *m*-C*H*$_{Ar}$), 6.55 (dd, 1H, $^3J_{HH}$ = 8.7 Hz, $^3J_{HH}$ = 6.7 Hz, C_6H_4N-Cq–CH–C*H*), 6.50 (dd, 1H, $^3J_{HH}$ = 8.8 Hz, $^3J_{HH}$ = 6.9 Hz, C_6H_4N-Cq–CH–C*H*), 6.43 (dd, 1H, $^3J_{HH}$ = 7.4 Hz, $^3J_{HH}$ = 6.9 Hz, C_6H_4N-N–CH–C*H*), 6.40 (dd, 1H, $^3J_{HH}$ = 7.4 Hz, $^3J_{HH}$ = 6.7 Hz, C_6H_4N-N–CH–C*H*), 4.96 (s, 1H, cAAC-C*H*), 4.87 (s, 1H, cAAC-C*H*), 3.96 (sept, 1H, $^3J_{HH}$ = 6.7 Hz, C*H*(CH_3)$_2$), 3.75 (sept, 1H, $^3J_{HH}$ = 6.7 Hz, C*H*(CH_3)$_2$), 3.69 (sept, 1H, $^3J_{HH}$ = 6.7 Hz, C*H*(CH_3)$_2$), 3.48 (sept, 1H, $^3J_{HH}$ = 6.7 Hz, C*H*(CH_3)$_2$), 2.56 (d, 1H, $^2J_{HH}$ = 13.2 Hz, Cq–C*H*$_2$–Cq), 2.30 (d, 1H, $^2J_{HH}$ = 13.8 Hz, Cq–C*H*$_2$–Cq), 2.21 (d, 1H, $^2J_{HH}$ = 13.2 Hz, Cq–C*H*$_2$–Cq), 1.97 (d, 1H, $^2J_{HH}$ = 13.8 Hz, Cq–C*H*$_2$–Cq), 1.82 (s, 3H, Cq(C*H*$_3$)$_2$–CH_2), 1.64 (s, 3H, Cq(C*H*$_3$)$_2$–CH_2), 1.50 (s, 3H, N–Cq(C*H*$_3$)$_2$), 1.49 (d, 3H, $^3J_{HH}$ = 6.7 Hz, CH(C*H*$_3$)$_2$), 1.43 (d, 3H, $^3J_{HH}$ = 6.7 Hz, CH(C*H*$_3$)$_2$), 1.35 (s, 3H, N–Cq(C*H*$_3$)$_2$), 1.33 (s, 3H, Cq(C*H*$_3$)$_2$–CH_2), 1.33 (d, 3H, $^3J_{HH}$ = 6.7 Hz, CH(C*H*$_3$)$_2$), 1.31 (s, 3H, N–Cq(C*H*$_3$)$_2$), 1.31 (d, 3H, $^3J_{HH}$ = 6.7 Hz, CH(C*H*$_3$)$_2$), 1.19 (s, 3H, N–Cq(C*H*$_3$)$_2$), 1.14 (d, 3H, $^3J_{HH}$ = 6.7 Hz, CH(C*H*$_3$)$_2$), 1.07 (d, 3H, $^3J_{HH}$ = 6.7 Hz, CH(C*H*$_3$)$_2$), 0.85 (s, 3H, Cq(C*H*$_3$)$_2$–CH_2), –0.60 (d, 3H, $^3J_{HH}$ = 6.6 Hz, CH(C*H*$_3$)$_2$), –0.70 (d, 3H, $^3J_{HH}$ = 6.6 Hz, CH(C*H*$_3$)$_2$) ppm.

^{13}C{^{1}H} NMR (125.8 MHz, 298 K, C_6D_6): δ = 152.1 (*o*-Dipp-*C*$_q$), 152.0 (*o*-Dipp-*C*$_q$), 151.0 (*o*-Dipp-*C*$_q$), 150.6 (C_5H_4N-*C*$_q$), 149.8 (*o*-Dipp-*C*$_q$), 149.6 (C_5H_4N-*C*$_q$), 141.8 (*i*-Dipp-*C*$_q$), 139.7 (*i*-Dipp-*C*$_q$), 136.4 (C_5H_4N-N–*C*H), 136.2 (C_5H_4N-C$_q$–*C*H), 132.0 (C_5H_4N-N–*C*H), 131.9 (C_5H_4N-C$_q$–*C*H), 126.5 (*p*-Dipp-*C*H), 125.7 (*p*-Dipp-*C*H), 125.24 (*m*-Dipp-*C*H), 125.21 (*m*-Dipp-*C*q), 124.8 (*m*-Dipp-*C*H), 124.5 (*m*-Dipp-*C*H), 118.7 (C_5H_4N-C$_q$CH–*C*H), 116.8 (C_5H_4N-C$_q$CH–*C*H), 115.7 (C_5H_4N-NCH–*C*H), 113.0 (C_5H_4N-NCH–*C*H), 70.0 (cAAC-*C*H), 68.6 (cAAC-*C*H), 65.6 (N–*C*$_q$(CH_3)$_2$), 64.6 (N–*C*$_q$(CH_3)$_2$), 58.5 (*C*$_q$(CH_3)$_2$–*C*H$_2$–C$_q$(CH)$_3$), 58.1 (C$_q$(CH_3)$_2$–*C*H$_2$–C$_q$(CH)$_3$), 41.2 (*C*$_q$(CH_3)$_2$–CH_2), 41.0 (*C*$_q$(CH_3)$_2$–CH_2), 35.1 (C$_q$(*C*H$_3$)$_2$–CH_2), 34.0 (C$_q$(*C*H$_3$)$_2$–CH_2), 32.9 (N–C$_q$(*C*H$_3$)$_2$), 32.1 (N–C$_q$(*C*H$_3$)$_2$), 31.5 (C$_q$(*C*H$_3$)$_2$–CH_2), 29.5 (N–C$_q$(*C*H$_3$)$_2$), 29.4 (C$_q$(*C*H$_3$)$_2$–CH_2), 29.3 (N–C$_q$(*C*H$_3$)$_2$, 29.0 (Dipp-*C*H(CH_3)$_2$), 28.8 (Dipp-*C*H(CH_3)$_2$), 28.0 (Dipp-*C*H(CH_3)$_2$), 27.4 (Dipp-*C*H(CH_3)$_2$), 26.7 (Dipp-CH(*C*H$_3$)$_2$), 26.3 (Dipp-CH(*C*H$_3$)$_2$), 24.9 (Dipp-CH(*C*H$_3$)$_2$), 24.87 (Dipp-CH(*C*H$_3$)$_2$), 24.83 (Dipp-CH(*C*H$_3$)$_2$), 24.6 (Dipp-CH(*C*H$_3$)$_2$), 24.3 (Dipp-CH(*C*H$_3$)$_2$), 24.0 (Dipp-CH(*C*H$_3$)$_2$) ppm.

^{11}B NMR (298 K, 160.5 MHz, C_6D_6): δ = 29.9 (br s) ppm.

HRMS (LIFDI): calcd. [$C_{50}H_{72}B_2N_4$ + H^+]$^+$, m/z = 751.6016; found: m/z = 751.6002.

Preparation of 7: 20 mg (30 µmol) B_2(SIDep)$_2$ (**1**) was dissolved in 0.6 mL C_6D_6 and this solution was treated with 2.8 mg (1.0 equiv) quinoline, resulting in an immediate color change from red to blue. ^{11}B NMR spectroscopic monitoring after 2 min showed complete consumption of the diboryne and one new, broad signal at 30 ppm and a small signal at 25 ppm. After one hour the signal at 25 ppm disappeared and the solution turned green. After evaporation of all volatiles the green residue was washed with hexane (3 x 0.6 mL) and dried in vacuo to obtain **7** in 61% yield as a colorless solid. Reduction of the volume of the washes provided further **7**, increasing the yield to 91%. Single crystals suitable for X-ray diffraction were obtained by slow evaporation of a saturated hexane solution of **7**.

^{1}H{^{11}B} NMR (500.1 MHz, C_6D_6): δ = 7.91 (d, 1H, $^3J_{H,H}$ = 8.0 Hz, C*H*$_{Ar,6\text{-}quin}$), 7.50 – 7.44 (m, 2H, C*H*$_{Ar,5+8\text{-}quin}$),

7.25 – 7.21 (m, 1H, C$H_{Ar,7\text{-}quin}$), 7.15 – 7.13 (m, 1H, C$H_{Ar,4\text{-}quin}$), 7.08 – 7.02 (m, 2H, C$H_{Ar,Dep}$), 6.93 – 6.83 (m, 10H, C$H_{Ar,p\text{-}Dep}$), 6.32 – 6.29 (d, 1H, $^3J_{H,H}$ = 8.0 Hz, C$H_{Ar,3\text{-}quin}$), 3.79 (s, 1H, B=C*H*), 3.44 (s, 4H, NC$H_{2,diazaborole}$), 3.11 (s, 4H, NC$H_{2,NHC}$), 2.84 – 2.75 (m, 4H, CH_{2Et}), 2.70 – 2.60 (m, 4H, CH_{2Et}), 2.41 (m, 8H, CH_{2Et}), 1.21 (t, 24H, $^3J_{H,H}$ = 7.6 Hz, CH_{3Et}), 1.03 (t, 24H, $^3J_{H,H}$ = 7.6 Hz, CH_{3Et}) ppm.

^{13}C{^{1}H} NMR (125.8 MHz, C_6D_6): δ = 189.3 ($C_{Carbene}$, detected by HMBC), 175.6 (N*C*B=C, detected by HMBC), 148.0 (C_q), 144.9 (C_q), 142.6 (C_q), 142.0 (C_q), 141.6 (C_q), 136.5 (C_q), 130.1 ($CH_{Ar,6\text{-}quin}$), 129.9 ($CH_{Ar,4\text{-}quin}$), 128.6 (CH_{Ar}), 127.5 ($CH_{Ar,3\text{-}quin}$), 127.4 ($CH_{Ar,8\text{-}quin}$), 126.8 ($CH_{Ar,5\text{-}quin}$), 126.5 (CH_{Ar}), 125.9 (CH_{Ar}), 124.5 (CH_{Ar}), 123.7 ($CH_{Ar7\text{-}quin}$), 104.3 (B=*C*H, detected by HSQC), 52.1 (N$CH_{2,diazaborole}$), 50.8 (N$CH_{2,NHC}$), 24.9 (CH_{2Et}), 23.9 (CH_{2Et}), 15.4 (CH_{2Et}), 14.0 (CH_{3Et}) ppm.

^{11}B{^{1}H} NMR (160.5 MHz, C_6D_6): δ = 29.7 (br., both boron nuclei) ppm.

^{15}N NMR (HMBC, 50.7 MHz, C_6D_6): δ = −60.1 (N_{quin}), −254.7 (N_{NHC}), −300.1 (*N*B*N*) ppm.

HRMS (LIFDI): m/z ($C_{55}H_{67}B_2N_5$) = calc.: 819.5577, found: 819.5564.

NMR Spectra

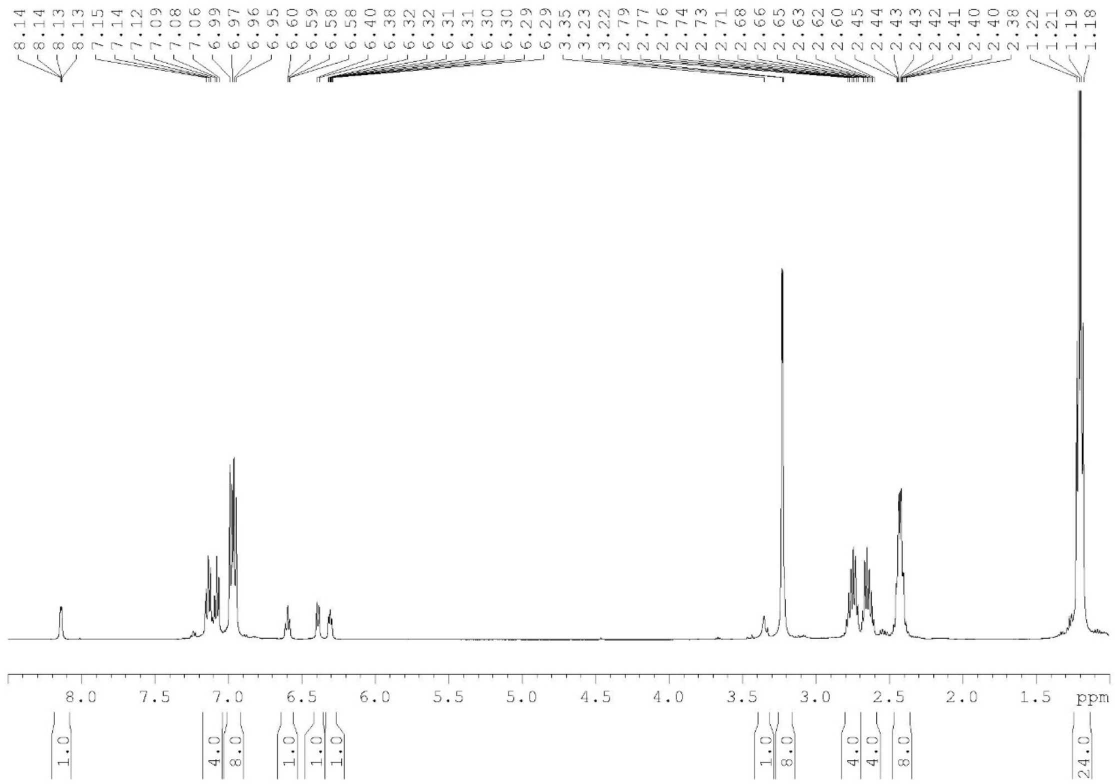

Figure S1. $^{1}H\{^{11}B\}$ spectrum of **3**.

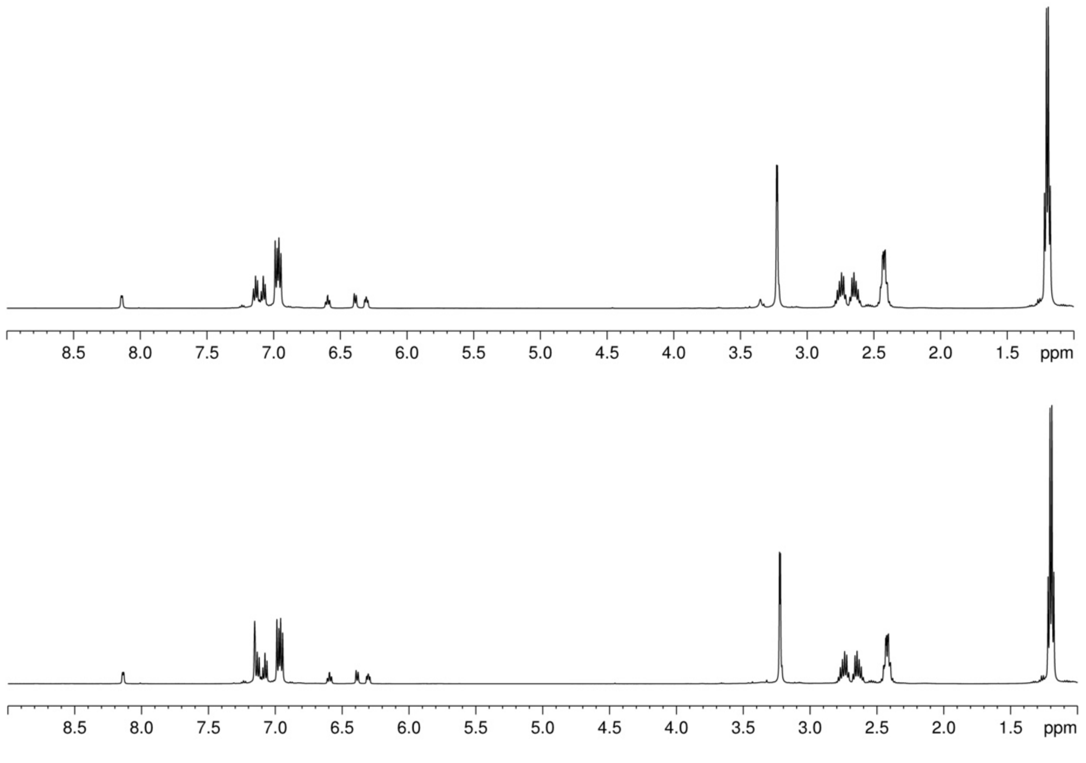

Figure S2. Stacked $^{1}H\{^{11}B\}$ (top) and ^{1}H (bottom) NMR spectra of **3**.

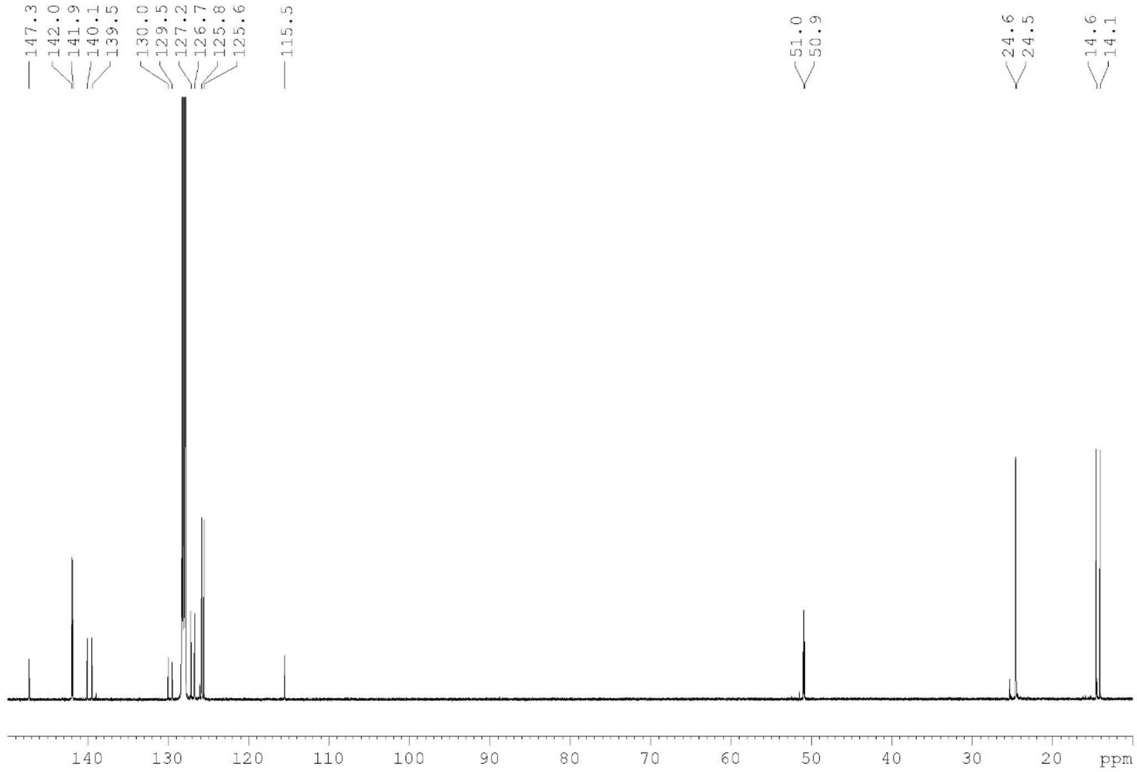

Figure S3. $^{13}C\{^{1}H\}$ NMR spectrum of **3**.

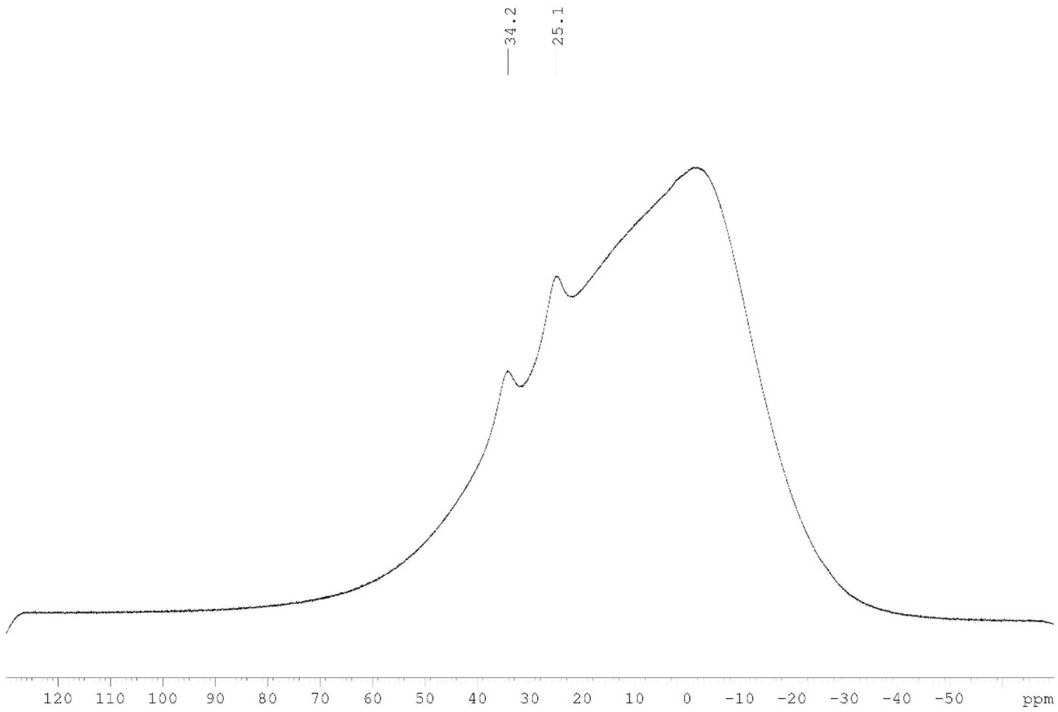

Figure S4. $^{11}B\{^{1}H\}$ NMR spectrum of **3**.

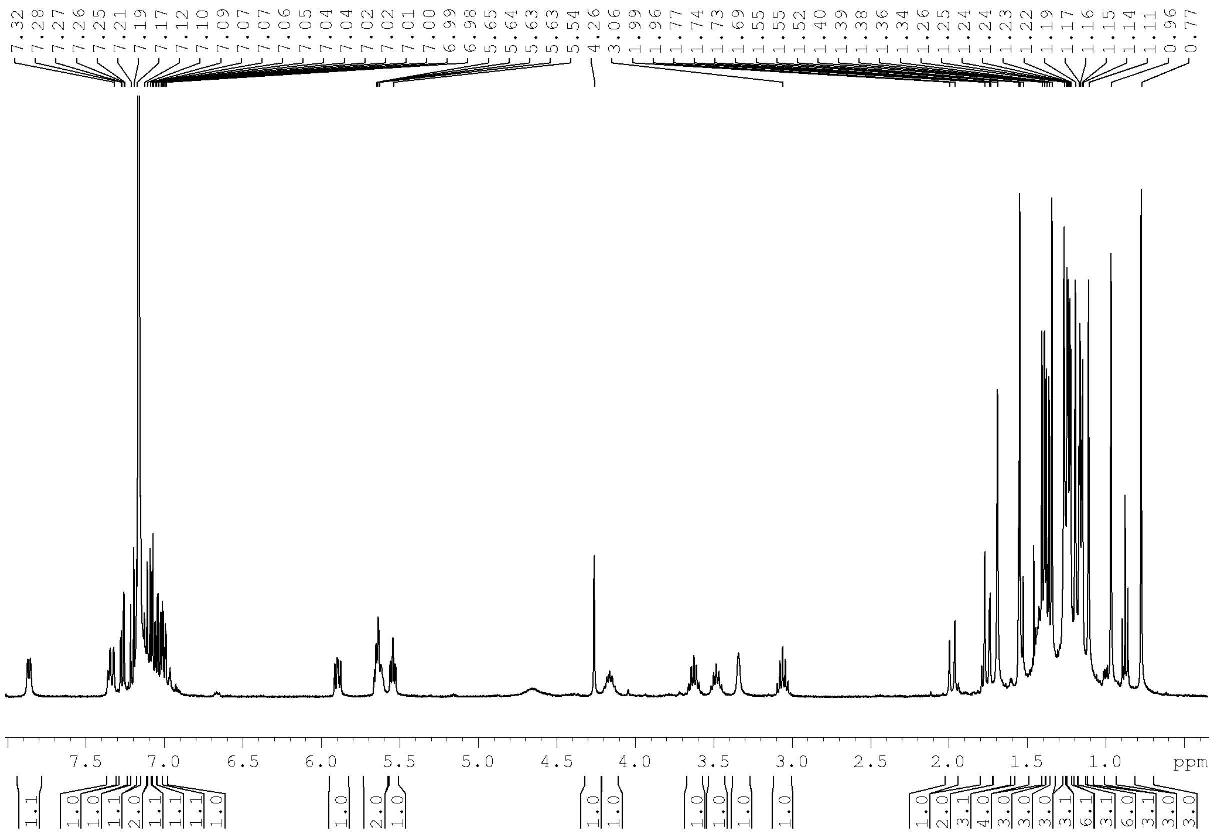

Figure S5. ^{1}H NMR spectrum of **4**.

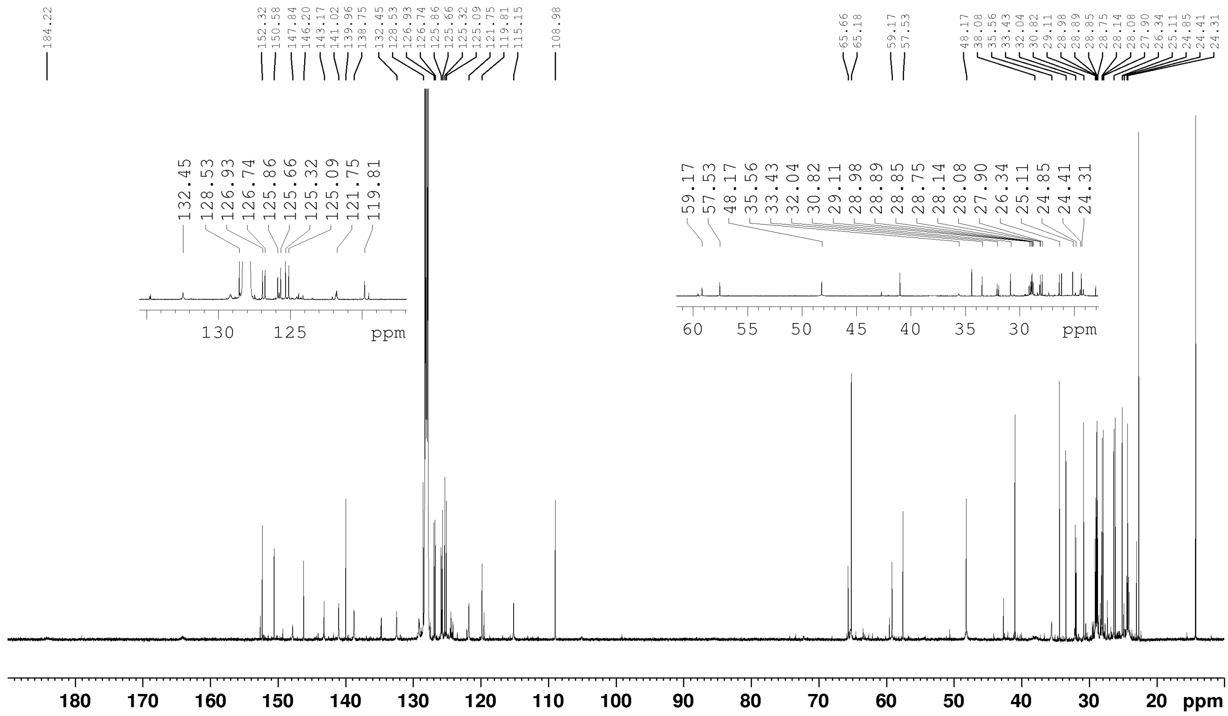

Figure S6. $^{13}C\{^{1}H\}$ NMR spectrum of **4**.

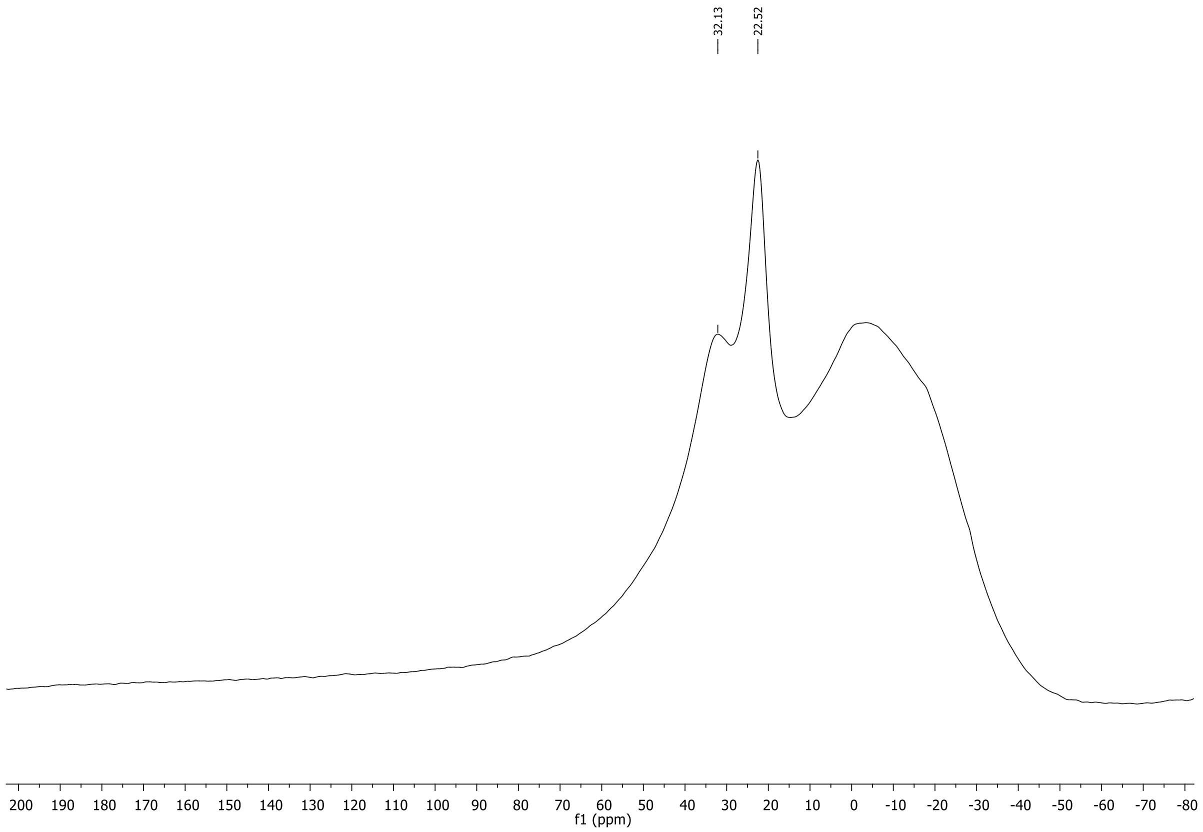

Figure S7. ^{11}B NMR spectrum of **4**.

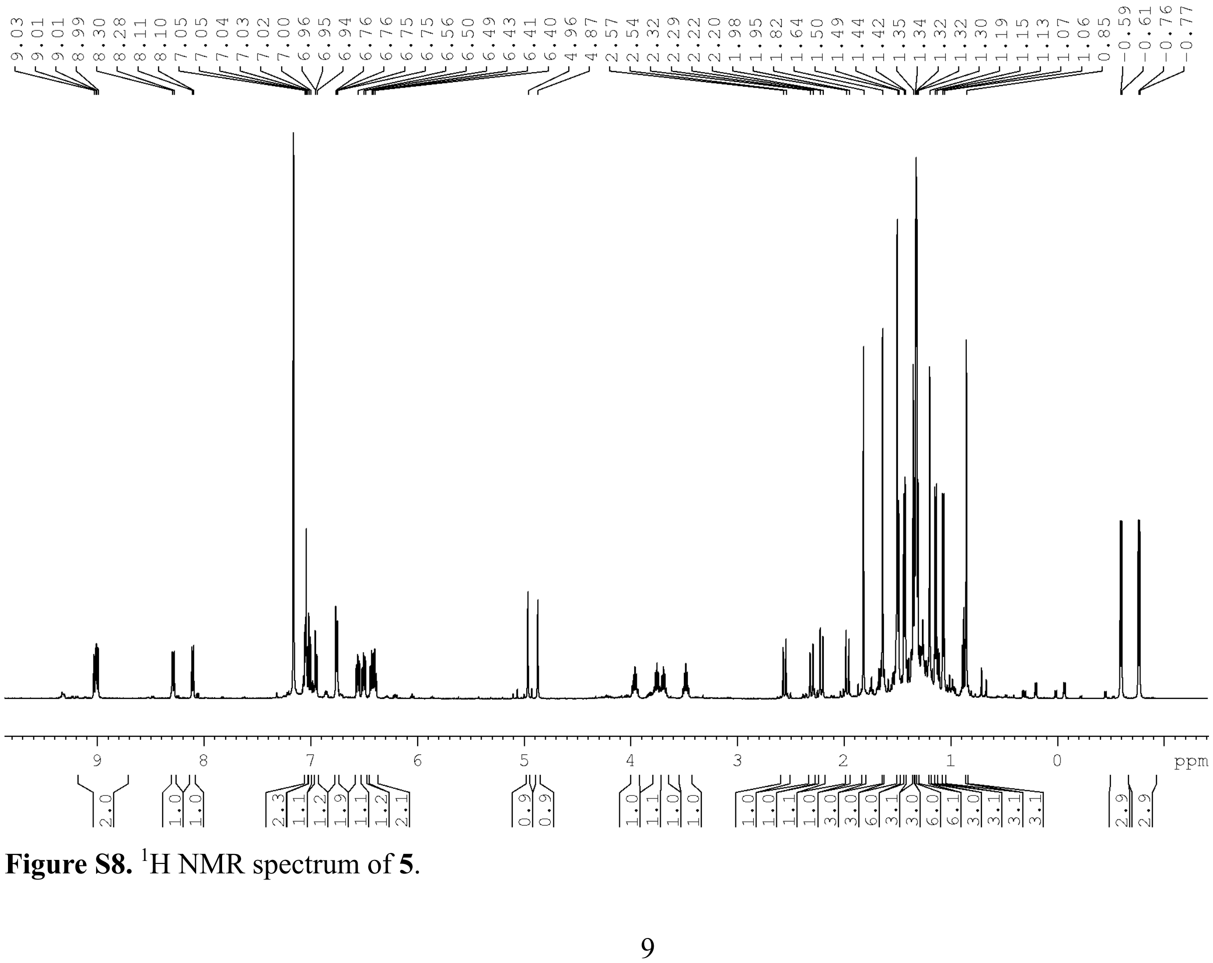

Figure S8. ^{1}H NMR spectrum of **5**.

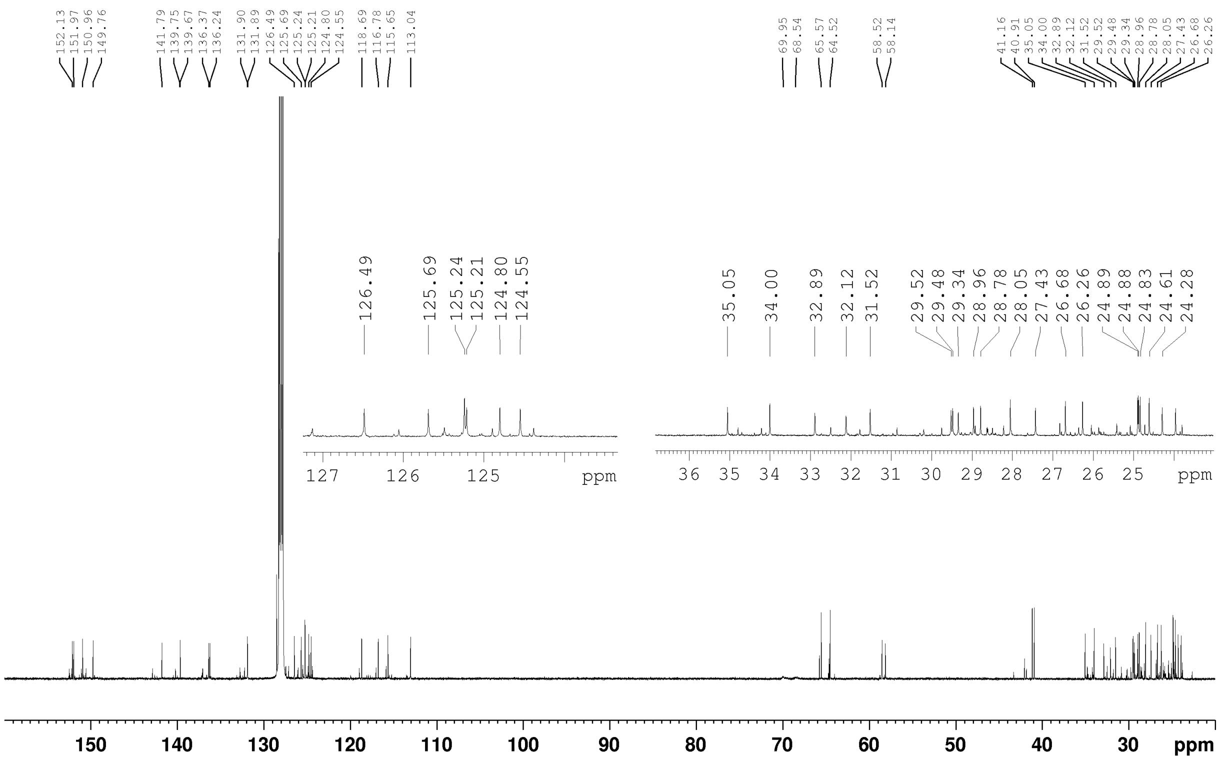

Figure S9. $^{13}C\{^{1}H\}$ NMR spectrum of **5**.

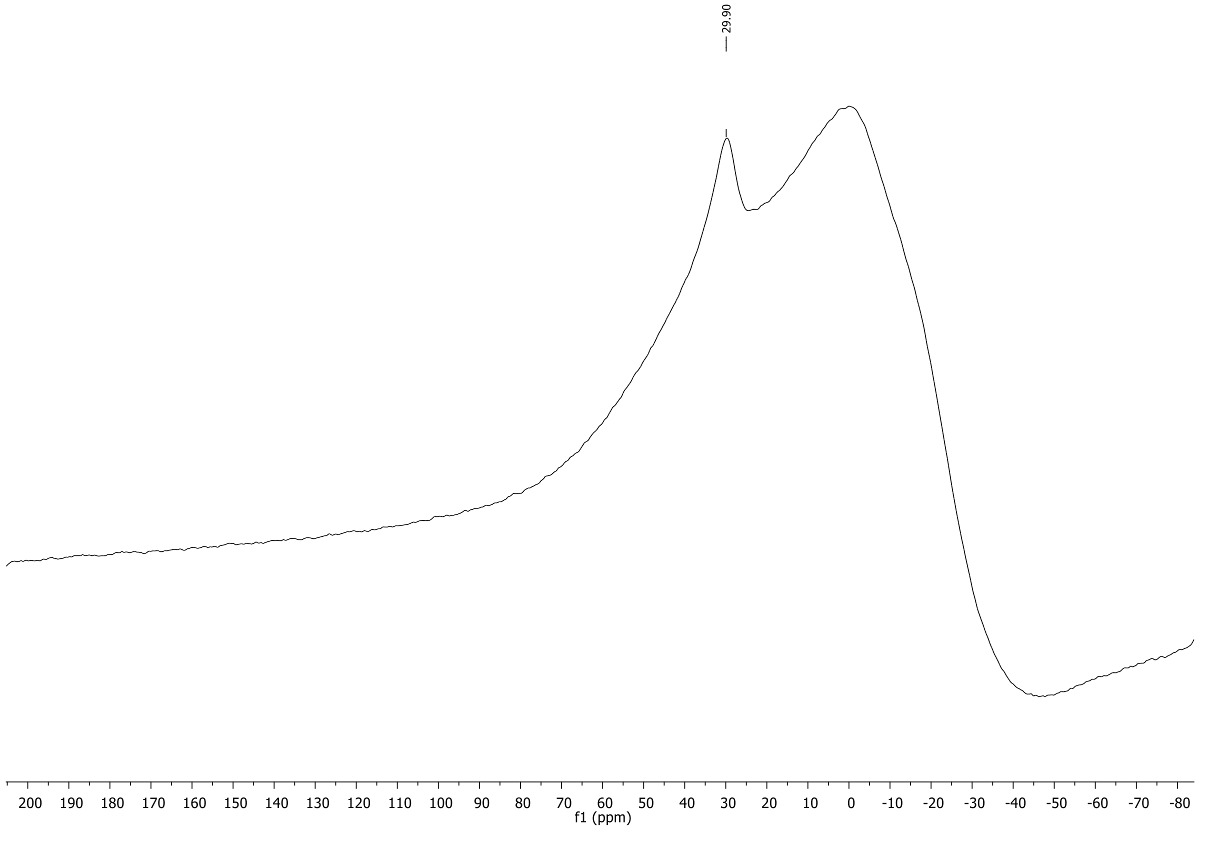

Figure S10. ^{11}B NMR spectrum of **5**.

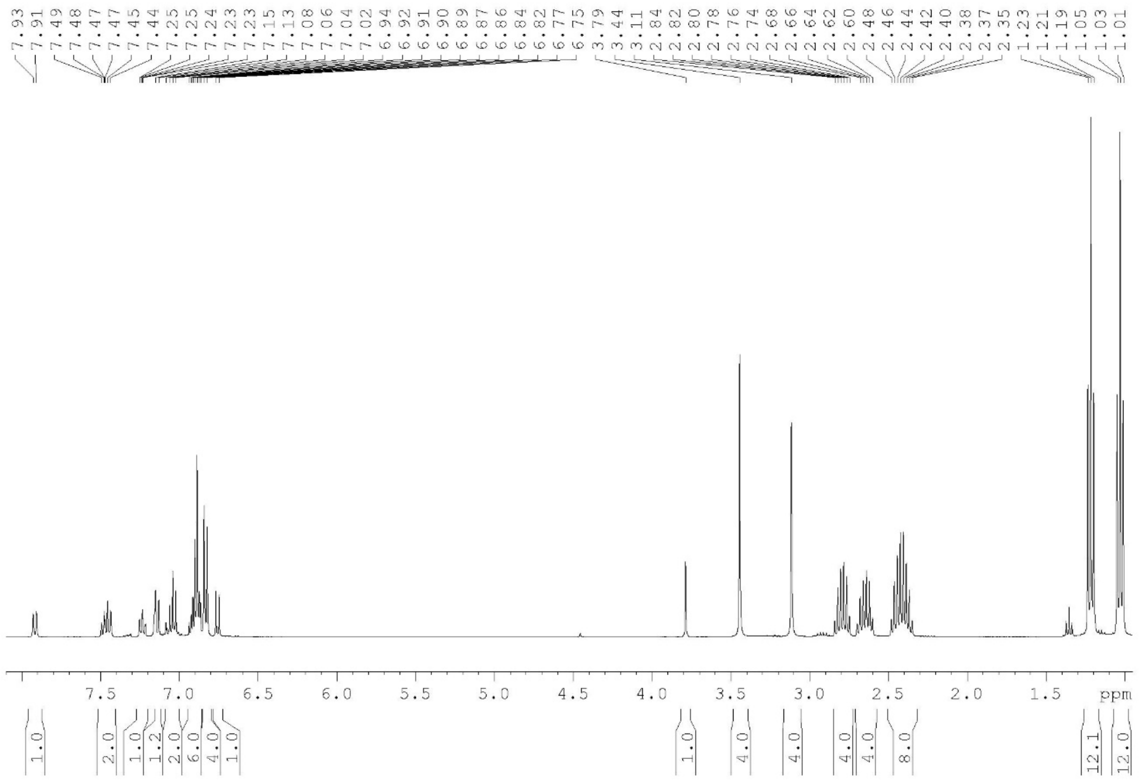

Figure S11. ^{1}H NMR spectrum of **7**.

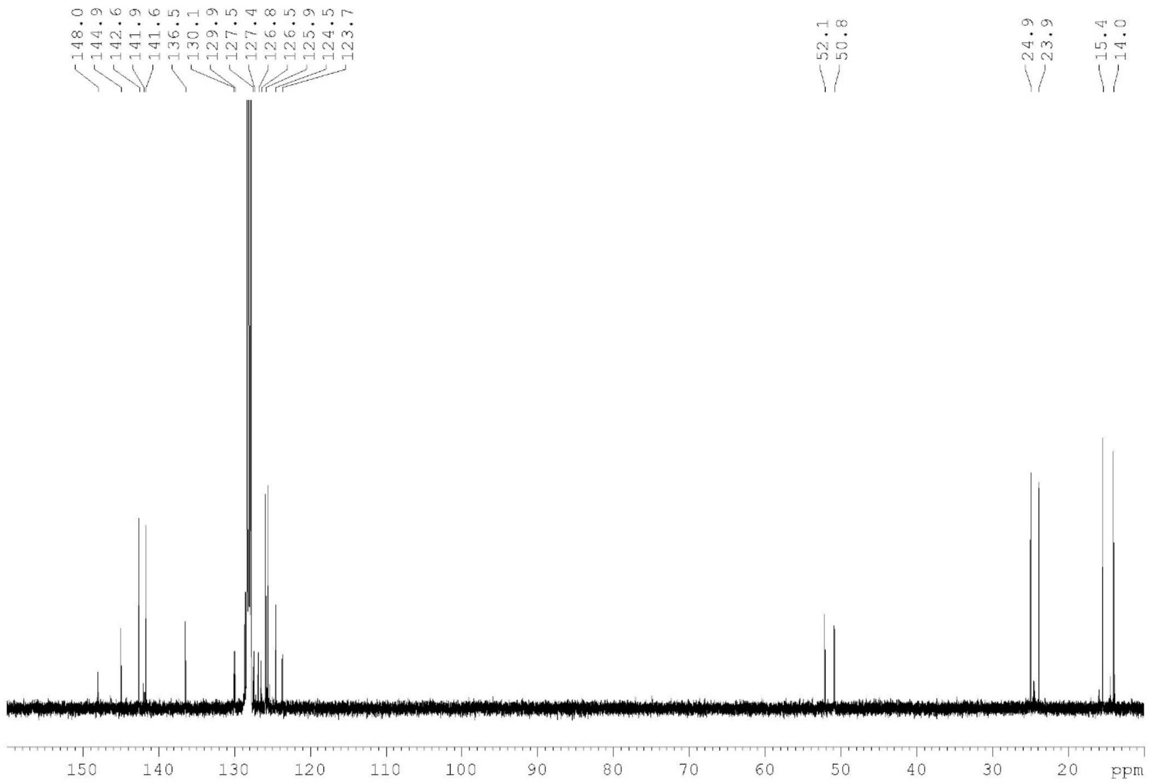

Figure S12. $^{13}C\{^{1}H\}$ NMR spectrum of **7**.

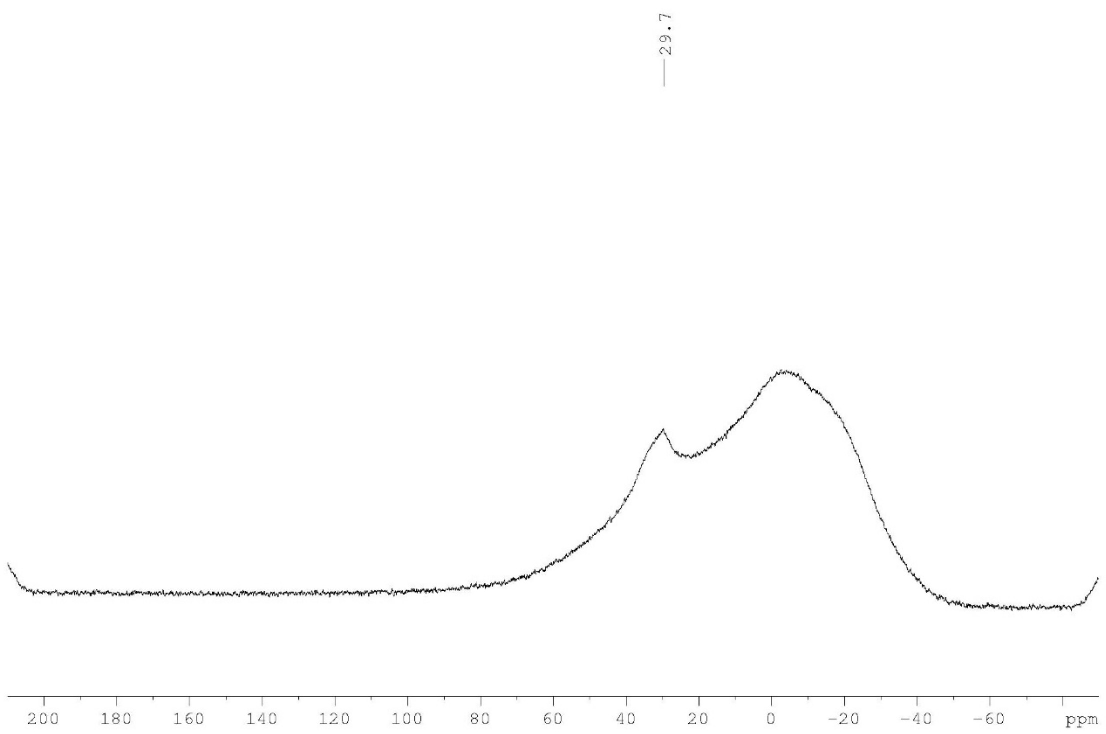

Figure S13. $^{11}B\{^{1}H\}$ NMR spectrum of **7**.

Crystallographic Details

The crystal data of **3**, **4**, **5**, and **7** were collected on a Bruker D8 Quest diffractometer with a CMOS area detector and multi-layer mirror monochromated MoK_{α} radiation. The structures were solved using the intrinsic phasing method,[5] refined with the SHELXL program[6] and expanded using Fourier techniques. All non-hydrogen atoms were refined anisotropically. Hydrogen atoms were included in structure factor calculations. Crystallographic data have been deposited with the Cambridge Crystallographic Data Center (CCDC numbers: 2204262 (**3**), 2204261 (**4**), 2204260 (**5**), 2204259 (**7**)). These data can be obtained free of charge from The Cambridge Crystallographic Data Centre via www.ccdc.cam.ac.uk/data_request/cif

Crystal data for **3**: $C_{51}H_{65}B_2N_5$, M_r = 769.70, green block, 0.28×0.275×0.232 mm^3, monoclinic space group $P2_1$, a = 12.5981(5) Å, b = 13.4918(5) Å, c = 26.2737(11) Å, β = 94.361(2)°, V = 4452.8(3) $Å^3$, Z = 4, ρ_{calcd} = 1.148 $g{\cdot}cm^{-3}$, μ = 0.066 mm^{-1}, F(000) = 1664, T = 100(2) K, R_1 = 0.0707, wR^2 = 0.1091, 16320 independent reflections [2θ≤52.044°] and 1069 parameters. The coordinates of H1 and H6 were refined freely.

Crystal data for **4**: $C_{56}H_{78}B_2N_4$, M_r = 828.84, violet block, 0.338×0.163×0.09 mm^3, monoclinic space group $P2_1/c$, a = 10.349(6) Å, b = 16.275(7) Å, c = 28.859(16) Å, β = 91.223(11)°, V = 4860(4) $Å^3$, Z = 4, ρ_{calcd} = 1.133 $g{\cdot}cm^{-3}$, μ = 0.065 mm^{-1}, F(000) = 1808, T = 100(2) K, R_1 = 0.1442, wR^2 = 0.1643, 9525 independent reflections [2θ≤52.042°] and 576 parameters.

Crystal data for **5**: $C_{53}H_{75}B_2N_4$, M_r = 789.79, red block, 0.288×0.274×0.273 mm^3, monoclinic space group $I2/a$, a = 18.70(3) Å, b = 10.573(16) Å, c = 47.37(8) Å, β = 90.33(3)°, V = 9367(26) $Å^3$, Z = 8, ρ_{calcd} = 1.120 $g{\cdot}cm^{-3}$, μ = 0.064 mm^{-1}, F(000) = 3448, T = 100(2) K, R_1 = 0.1137, wR^2 = 0.1769, 8282 independent reflections [2θ≤50.046°] and 676 parameters. The displacement parameters of atoms B1, B2, N1, N2 and C1-C10 of the residues 11 and 111 (Main) were restrained to the same value with similarity restraint SIMU and RIGU.

Crystal data for **7**: $C_{55}H_{67}B_2N_5$, M_r = 819.75, yellow block, 0.312×0.245×0.223 mm^3, monoclinic space group $P2_1/c$, a = 22.6082(5) Å, b = 10.9231(2) Å, c = 20.1980(5) Å, β = 110.9680(10)°, V = 4657.63(18) $Å^3$, Z = 4, ρ_{calcd} = 1.169 $g{\cdot}cm^{-3}$, μ = 0.067 mm^{-1}, F(000) = 1768, T = 100(2) K, R_1 = 0.0653, wR^2 = 0.1197, 9172 independent reflections [2θ≤52.044°] and 671 parameters. The displacement parameters of atoms of the disordered groups were restrained to the same value with similarity restraint SIMU and RIGU. The distances between atoms of the disordered NHC backbone were restrained during refinement to the same value with the SADI restraint.

Computational Details

All electronic structure calculations were performed using the Gaussian16 rev. B.01 package.[7] Gas-phase geometry optimizations were carried out (no constraints or symmetry restrictions were used) with the long-range hybrid functional ωB97X-D[8] in conjunction with Pople's double-ζ 6-31G(d,p) basis set for all the atoms. Subsequent harmonic frequency calculations were performed to corroborate the character of each optimized species. Depending on the number of negative eigenvalues of the Hessian matrix, we can classify each optimized structure as minimum (zero) or transition state (only one). Thermal and entropy corrections to the total energy were taken from the thermochemistry analyses in the output file at 298 K and 1 atm. For systems **4** and **5**, nucleus-independent chemical shift (NICS)[9] calculations were performed at their corresponding optimized geometries using the gauge-independent atomic orbital (GIAO)[10] method. These calculations were done at the ωB97X-D/6-311++G(d,p) level. For the mechanistic calculations, the solvation effects added to the electronic Hamiltonian were taken into consideration by performing single-point calculations over the optimized geometries with a larger basis set (6-311++G(d,p) for all the atoms) though the PCM model[11] using the SMD[12] parameters according to Truhlar's model with benzene (ε = 2.2706), as the solvent used experimentally. These energies were added to the gas-phase calculations. Therefore, the final reported energy values are in solution phase, calculated at the SMD(benzene):ωB97X-D/6-311++G(d,p)//ωB97X-D/6-31G(d,p) level.

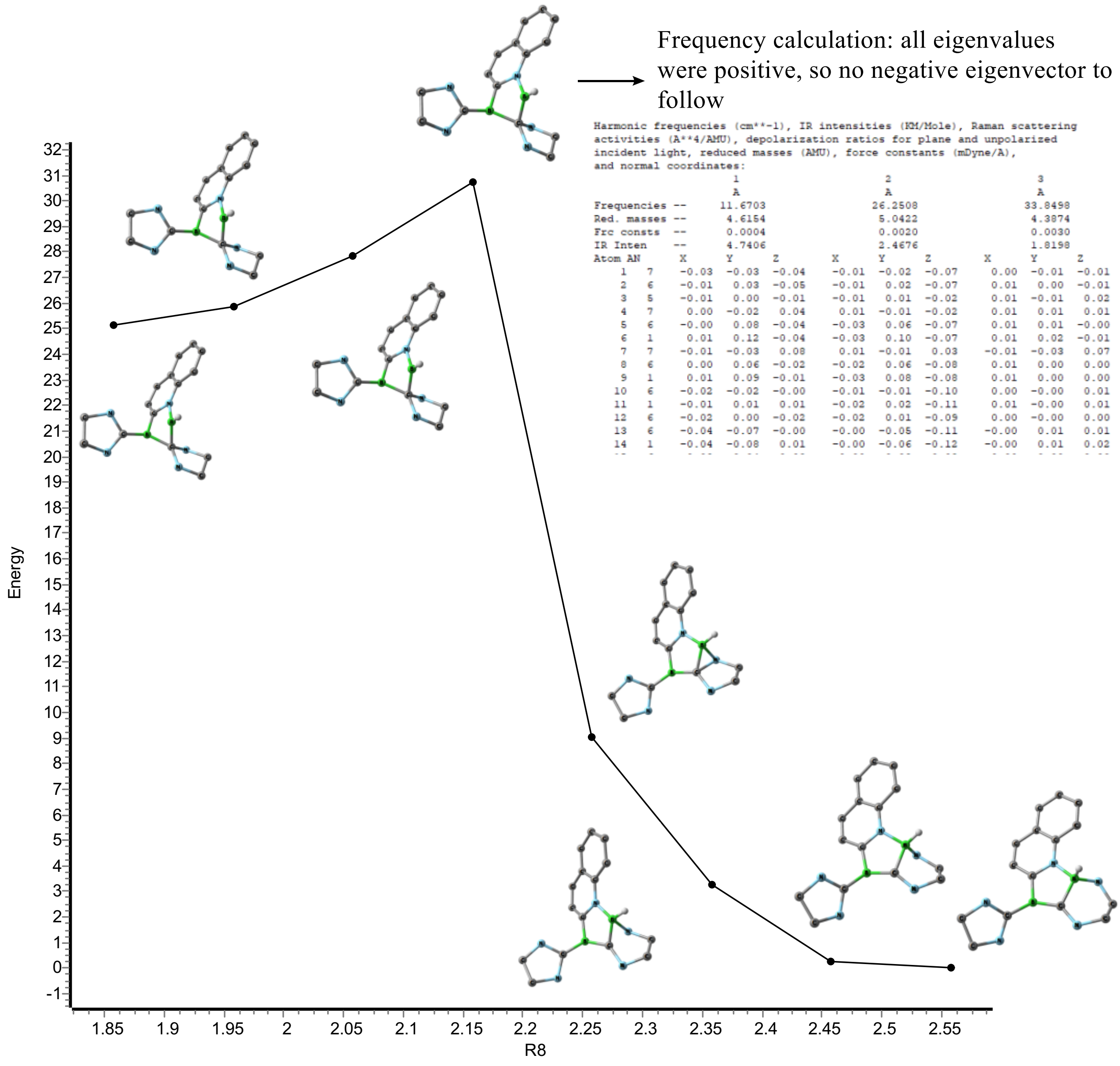

Figure S14. Relaxed scan performed on the geometry of **A5** to confirm that it converts into intermediate **A6**. Hydrogen atoms and some groups were deleted for the sake of clarity in our figures. Although there is a maximum point, when computing harmonic frequencies, it did not show any negative value that could indicate a maximum in this direction.

Table S1. Calculated multi-center indices (MCIs) the rings of compounds **4** and **5**. The larger the MCI value, the more aromatic the ring is.

Compound	Ring	MCI
4	A	0.5570
	B	0.3215
	C	0.3581
5	A	0.5452
	B	0.4960
	C	0.5459

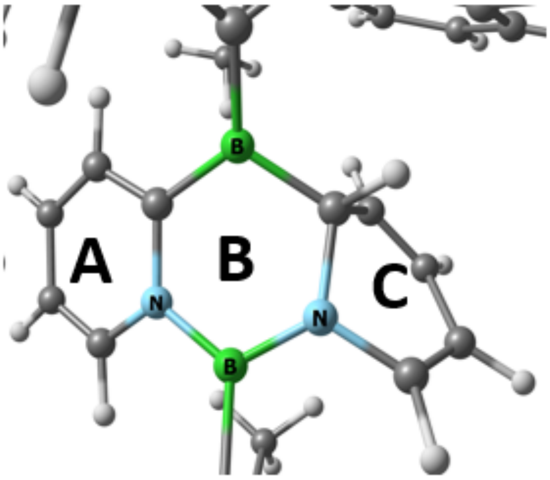

4

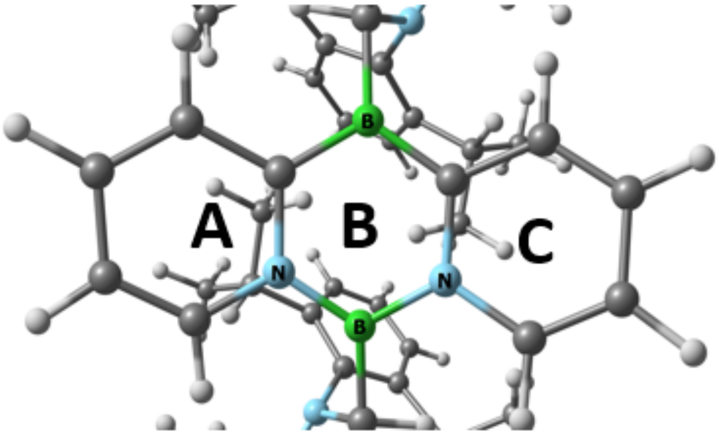

5

To assess aromaticity in fused ring systems, the multi-center index (MCI) has been widely used as an electronic aromaticity index that does not suffer of contamination of the adjacent rings. In contrast, magnetic aromaticity indices do because of the ring currents and lines of magnetic fields such as NICS. MCI is a particular extension of the I_{ring} index.

$$I_{ring}(A) = \sum_{i_1, i_2, \ldots, i_N} n_{i_1} n_{i_2} \ldots n_{i_N} S_{i_1 i_2}(A_1) S_{i_2 i_3}(A_2) \ldots S_{i_N i_1}(A_N)$$

n_i being the occupancy of MO i and $S_{ij}(A)$ the overlap between MOs i and j within the molecular space assigned to atom A. Summing up all the the I_{ring} values resulting from the permutations of indices $A_1, A_2, \ldots, A_N$ the mentioned MCI index is defined as:

$$MCI(A) = \frac{1}{2N} \sum_{P(A)} I_{ring}(A)$$

where *P(A)* stands for a permutation operator which interchanges the atomic labels $A_1, A_2, \ldots, A_N$ to generate up to the *N!* permutations of the elements in the string *A*. MCI gives an idea of the electron sharing between all the atoms in the ring. The more positive the MCI values, the more aromatic the rings. In this case, as the trend pointed out by our calculated MCI values is the same as for the NICS(1)$_{ZZ}$ reported values, we can say those measurements are reliable to assess the aromaticity in compounds **4** and **5**.

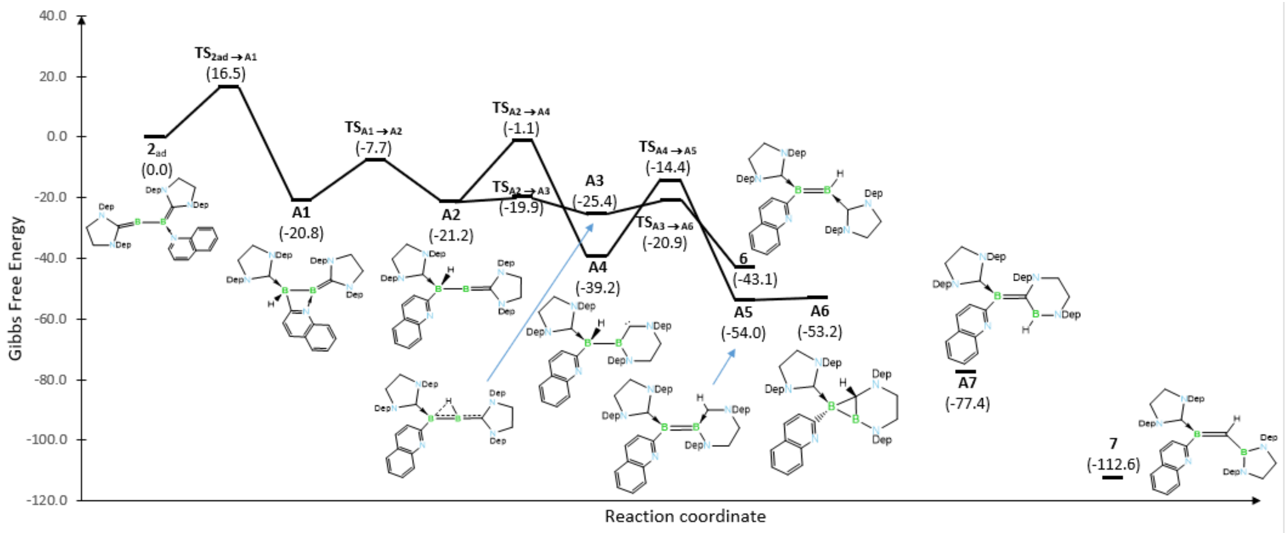

Figure S15. Other mechanistic routes explored initially.

Cartesian coordinates

Table S1. Cartesian coordinates (x-y-z format) for species **3-5**, calculated at the ωB97X-D/6-31G(d,p) level.

**3**				**4**			
E(scf) = –2305.48094795 a.u.				E(scf) = –2216.92151539 a.u.			
N	2.878117	1.049234	0.756902	N	-0.914358	0.817613	-1.393438
B	-0.806130	0.101188	0.048966	B	1.385687	0.571978	-0.370858
H	-1.696894	-0.644791	0.358832	C	-1.810573	1.445673	-2.222296
C	2.003041	0.531794	-0.138471	H	-2.765218	0.955292	-2.319602
N	2.005320	-2.355459	1.018687	N	-0.251118	-1.371649	-0.432982
C	2.270087	0.631131	-1.517114	B	-1.288417	-0.512687	-0.844131
H	1.566645	0.188871	-2.215259	C	-1.541942	2.612067	-2.860088
B	0.701831	-0.187402	0.418330	H	-2.286775	3.054716	-3.509246
N	0.202800	-2.032904	2.216150	C	1.807098	-1.784435	-1.627400
C	3.435943	1.224985	-1.969419	H	2.600534	-1.325945	-2.209108
H	3.660159	1.265600	-3.032165	C	1.192098	-1.028729	-0.467041
N	-2.457025	0.940894	-1.697240	H	1.616405	-1.457284	0.454264
C	4.311665	1.776046	-1.036905	C	0.350751	1.369713	-1.146263
H	5.232260	2.266300	-1.336715	C	0.620984	2.598073	-1.821577
N	-0.998777	2.467279	-1.104248	H	1.616479	3.001177	-1.714095
C	3.971459	1.667632	0.306017	C	-0.270579	3.208245	-2.652062
H	4.629262	2.084313	1.068360	H	-0.001368	4.122084	-3.171925
C	1.917159	-3.562817	1.837131	C	0.345487	-3.601674	-1.030023
H	2.878967	-3.800189	2.298831	H	0.188833	-4.673220	-0.971699
H	1.615651	-4.417386	1.217173	C	1.399358	-3.041117	-1.865307
C	0.844341	-3.180885	2.854160	H	1.839562	-3.645355	-2.652401
H	0.126566	-3.983230	3.045575	C	-0.456469	-2.769528	-0.342810
H	1.277569	-2.875108	3.815544	H	-1.295788	-3.127720	0.241069
C	0.960044	-1.505900	1.219223	C	2.434623	1.158961	0.518254
C	2.772334	-2.394667	-0.186903	C	4.281130	1.067706	2.089011
C	2.157027	-2.806751	-1.379972	C	3.756056	2.506093	2.014567
C	2.939333	-2.819705	-2.535237	H	3.744683	2.999370	2.991139
H	2.500246	-3.122737	-3.479842	H	4.417087	3.089090	1.361472
C	4.277778	-2.452551	-2.494078	C	2.359370	2.438245	1.376166
H	4.867417	-2.463372	-3.405810	C	3.936630	0.425849	3.447244
C	4.129612	-2.050488	-0.115212	H	4.475426	-0.508938	3.601514

C	4.870739	-2.079473	-1.294357	H	2.869466	0.220302	3.539460
H	5.916914	-1.795312	-1.282264	H	4.224223	1.104838	4.256271
C	4.725384	-1.640046	1.214435	C	5.798086	1.030887	1.904866
H	4.738183	-2.512127	1.882593	H	6.181078	0.008274	1.865721
H	4.046147	-0.912732	1.670802	H	6.271979	1.541143	2.748864
C	6.129152	-1.047626	1.150003	H	6.098137	1.539287	0.986783
H	6.866320	-1.769569	0.783664	C	1.246985	2.275828	2.434302
H	6.446185	-0.728785	2.146599	H	1.338607	1.345829	2.996685
H	6.150329	-0.171818	0.494548	H	0.271447	2.258816	1.944849
C	0.688741	-3.178287	-1.412377	H	1.267601	3.111981	3.143757
H	0.098988	-2.251095	-1.426243	C	2.095766	3.768812	0.657669
H	0.413184	-3.654832	-0.465774	H	2.246647	4.585811	1.373056
C	0.264927	-4.098339	-2.554362	H	1.071722	3.842447	0.288801
H	0.861029	-5.016372	-2.575325	H	2.789313	3.928408	-0.174398
H	0.362097	-3.618641	-3.533973	N	3.553847	0.438523	0.947508
H	-0.786627	-4.371482	-2.434077	C	4.228145	-0.468505	0.061672
C	-0.842644	-1.362838	2.920308	C	4.388662	-1.829466	0.398860
C	-2.158760	-1.811090	2.717949	C	5.065527	-2.671951	-0.485319
C	-2.893246	-0.134050	4.301743	H	5.186570	-3.719589	-0.227353
H	-3.700714	0.354944	4.838405	C	5.572438	-2.206447	-1.685188
C	-3.178062	-1.171609	3.417493	H	6.087483	-2.879561	-2.363272
H	-4.208028	-1.480099	3.275589	C	5.420351	-0.866376	-2.007735
C	-0.527378	-0.350316	3.831497	H	5.827650	-0.496053	-2.944134
C	-1.582275	0.262618	4.513229	C	3.878200	-2.434642	1.697053
H	-1.363262	1.058254	5.220190	H	3.263047	-1.679707	2.190971
C	0.891419	0.101885	4.097910	C	6.064578	2.098074	-1.825723
H	1.606348	-0.547195	3.586556	H	6.708310	2.000294	-0.947394
H	1.086594	0.005995	5.173579	H	5.964025	3.163573	-2.057065
C	1.148577	1.539898	3.647566	H	6.583682	1.627064	-2.666759
H	2.179148	1.834772	3.861067	C	4.679945	1.475373	-1.597963
H	0.477855	2.238569	4.158785	H	4.184615	2.044969	-0.810154
H	1.001582	1.626038	2.570098	C	2.997357	-3.672728	1.482671
C	-2.428063	-2.927618	1.729809	H	3.562815	-4.498075	1.037781
H	-1.746813	-3.761691	1.935216	H	2.146697	-3.462988	0.833487
H	-2.156568	-2.563500	0.731602	H	2.613812	-4.022730	2.446779
C	-1.365495	1.171915	-0.906203	C	5.051612	-2.806442	2.617747

C	-3.855634	-3.462494	1.707986
H	-4.156091	-3.859327	2.683258
H	-3.941534	-4.266038	0.971674
H	-4.570996	-2.685810	1.423844
C	-2.697760	2.037497	-2.627767
H	-3.766065	2.211930	-2.774727
H	-2.244430	1.829220	-3.606204
C	-1.984481	3.181067	-1.920020
H	-1.504844	3.875759	-2.610252
H	-2.662566	3.749552	-1.267380
C	0.975913	3.851025	-0.822270
C	-0.145430	3.233880	-0.251151
C	1.799574	4.621118	-0.001176
H	2.690673	5.074610	-0.426632
C	1.491434	4.808746	1.336114
H	2.139720	5.408882	1.966810
C	0.343135	4.236841	1.869966
H	0.104063	4.408829	2.913197
C	-0.497548	3.438026	1.096914
C	-1.726948	2.786975	1.692726
H	-1.486782	1.732240	1.884539
H	-2.524301	2.764302	0.939896
C	-2.271712	3.427488	2.965553
H	-1.576033	3.321735	3.803171
H	-3.198638	2.927012	3.258629
H	-2.482036	4.493618	2.830357
C	1.295279	3.767228	-2.296322
H	0.747960	2.937893	-2.752799
H	2.356279	3.528570	-2.406435
C	1.005749	5.080830	-3.034162
H	1.222873	4.984996	-4.102361
H	1.621932	5.892755	-2.636300
H	-0.038518	5.390906	-2.925473
C	-2.951631	-0.370847	-1.964807
C	-2.338713	-1.159247	-2.947186
C	-2.874425	-2.422654	-3.208048
H	5.622734	-3.638326	2.191661
H	4.686710	-3.121354	3.601145
H	5.748228	-1.976309	2.760868
C	3.826659	1.604692	-2.866550
H	4.307050	1.108184	-3.716518
H	3.690048	2.658523	-3.133649
H	2.841985	1.155825	-2.716804
C	4.768790	0.021752	-1.153189
C	-2.799371	-1.056891	-0.792604
H	-2.754302	-1.847661	-0.041025
N	-3.852150	-0.084563	-0.411748
C	-5.081895	-0.235458	-1.233129
C	-4.542997	-0.919337	-2.501163
H	-5.311836	-1.535076	-2.978941
H	-4.260280	-0.155614	-3.235513
C	-3.303808	-1.752103	-2.113322
C	-5.689684	1.129379	-1.579026
H	-4.961130	1.786106	-2.061505
H	-6.059775	1.626222	-0.678312
H	-6.535671	1.000861	-2.261579
C	-6.206963	-1.059900	-0.579228
H	-5.935857	-2.104384	-0.424766
H	-7.094518	-1.043267	-1.219340
H	-6.483132	-0.628812	0.387364
C	-3.677339	-3.219182	-1.838652
H	-2.785730	-3.792277	-1.567319
H	-4.109438	-3.677025	-2.735050
H	-4.404757	-3.324926	-1.030292
C	-2.275402	-1.764564	-3.256071
H	-1.975212	-0.758657	-3.561422
H	-2.706170	-2.255788	-4.135353
H	-1.373056	-2.316017	-2.973759
C	-3.964621	0.321952	0.967615
C	-4.200053	-0.598348	2.025868
C	-4.313989	-0.129475	3.335473
H	-4.480945	-0.842567	4.137246

H	-2.407881	-3.047408	-3.965194
C	-3.974785	-2.889879	-2.504524
H	-4.379354	-3.874381	-2.718038
C	-4.546629	-2.103381	-1.509920
H	-5.389378	-2.483252	-0.939514
C	-4.040651	-0.839252	-1.213345
C	-4.607495	-0.042530	-0.059805
H	-3.781983	0.253097	0.597510
H	-5.246648	-0.710306	0.528063
C	-5.425786	1.190991	-0.452270
H	-4.792950	1.963704	-0.894420
H	-5.903174	1.625721	0.430761
H	-6.210136	0.936040	-1.171641
C	-1.119798	-0.699831	-3.716667
H	-0.683471	0.184331	-3.246083
H	-0.352443	-1.478077	-3.641819
C	-1.411042	-0.426282	-5.196184
H	-0.507688	-0.093317	-5.715199
H	-2.177539	0.346001	-5.315768
H	-1.777039	-1.326590	-5.698854
C	-4.224393	1.217536	3.640242
H	-4.326777	1.559669	4.665280
C	-3.988388	2.115892	2.616542
H	-3.893572	3.172891	2.847296
C	-3.843136	1.697050	1.292169
C	-4.287866	-2.110272	1.855165
H	-4.315548	-2.336267	0.793164
C	-3.487119	2.771360	0.278832
H	-3.461701	2.298131	-0.698201
C	-5.560446	-2.716947	2.460202
H	-5.613064	-3.784643	2.222621
H	-6.459812	-2.236325	2.067538
H	-5.579856	-2.624906	3.550302
C	-3.039961	-2.781857	2.449680
H	-2.121760	-2.310670	2.087145
H	-3.012082	-3.847555	2.197386
H	-3.036229	-2.696159	3.541280
C	-4.503860	3.918521	0.227522
H	-4.252958	4.601895	-0.590566
H	-4.499731	4.503351	1.153425
H	-5.523146	3.558734	0.067861
C	-2.085129	3.327463	0.558923
H	-1.345369	2.524960	0.580060
H	-2.046503	3.846188	1.523028
H	-1.789760	4.036597	-0.220806

5

E(scf) = –2216.97152186 a.u.

N	0.688316	-2.083627	0.722519
N	0.733840	-1.340252	-1.614539
B	1.461841	-1.551187	-0.383157
B	-1.494154	-1.525195	-0.431905
C	1.460379	-1.127647	-2.779440
H	2.521240	-1.023126	-2.638687
C	0.894548	-1.046504	-4.003146

H	1.529745	-0.888572	-4.866740
C	-0.518113	-1.169103	-4.127568
H	-0.992865	-1.133030	-5.101663
C	-1.249025	-1.322213	-2.990863
H	-2.327063	-1.411501	-3.054270
C	-0.670516	-1.389032	-1.686716
C	-0.716223	-2.007889	0.763489
C	-1.347035	-2.452655	1.962719
H	-2.420747	-2.334164	2.004779
C	-0.673870	-2.989760	3.014414
H	-1.192013	-3.315802	3.909123
C	0.737709	-3.129419	2.898335
H	1.327344	-3.594227	3.679582
C	1.359854	-2.686126	1.783825
H	2.424635	-2.810313	1.660466
C	-3.073486	-1.176440	-0.458517
H	-3.242405	-0.690744	-1.426703
N	-3.478747	-0.228734	0.615345
C	-4.737762	-0.607666	1.291505
C	-4.858139	-2.103088	0.941417
H	-5.906538	-2.416009	0.889166
H	-4.398160	-2.702792	1.735104
C	-4.117809	-2.345326	-0.389856
C	-4.630634	-0.415404	2.810786
H	-4.538601	0.646651	3.055899
H	-3.764130	-0.938839	3.222274
H	-5.529643	-0.796380	3.306304
C	-5.988528	0.177163	0.847516
H	-6.845440	-0.114201	1.463452
H	-6.258602	-0.002627	-0.193440
H	-5.830099	1.251111	0.982400
C	-3.494158	-3.746344	-0.427559
H	-2.814741	-3.922027	0.408866
H	-2.923851	-3.894712	-1.350809
H	-4.279264	-4.510391	-0.390229
C	-5.085821	-2.261155	-1.584138

H	-5.858166	-3.035402	-1.514496
H	-4.546593	-2.415103	-2.525968
H	-5.590963	-1.294948	-1.649340
C	-3.079960	1.149044	0.525819
C	-2.182942	1.677106	1.488706
C	-1.817500	3.024123	1.440261
H	-1.119021	3.407223	2.178779
C	-2.302806	3.869317	0.458644
H	-2.012101	4.915436	0.435356
C	-3.146938	3.352350	-0.510543
H	-3.506563	4.003879	-1.301861
C	-3.541582	2.013427	-0.503362
C	-1.520307	0.821216	2.550703
H	-1.928963	-0.179779	2.444032
C	-4.420382	1.560649	-1.661272
H	-4.740661	0.541954	-1.460109
C	-0.006786	0.743479	2.313904
H	0.488250	1.688384	2.555964
H	0.226330	0.516715	1.271123
H	0.432860	-0.038705	2.942392
C	-1.792693	1.303457	3.980417
H	-2.861529	1.374167	4.195076
H	-1.349942	2.288885	4.162029
H	-1.347703	0.606959	4.699428
C	-5.688595	2.407961	-1.826972
H	-6.261057	2.464547	-0.898115
H	-6.332307	1.970807	-2.597724
H	-5.455844	3.430736	-2.139774
C	-3.618617	1.546006	-2.970801
H	-4.218778	1.128350	-3.786828
H	-2.706683	0.952341	-2.872226
H	-3.319804	2.560343	-3.256753
C	3.025531	-1.207447	-0.161618
H	3.151844	-1.214417	0.924552
N	3.417762	0.133416	-0.656132
C	4.690487	0.122946	-1.419323

C	4.845438	-1.365851	-1.790654
H	5.901323	-1.648563	-1.850826
H	4.426206	-1.547465	-2.786842
C	4.091345	-2.207287	-0.738589
C	4.585217	0.987941	-2.683011
H	3.724448	0.710606	-3.296407
H	5.488959	0.879825	-3.291248
H	4.487317	2.043113	-2.414816
C	5.920435	0.637658	-0.647355
H	5.733622	1.643018	-0.259578
H	6.779765	0.693682	-1.322750
H	6.202564	-0.005754	0.186400
C	5.051709	-2.682234	0.366845
H	5.557076	-1.855851	0.870021
H	5.824073	-3.334594	-0.054256
H	4.519675	-3.259170	1.131701
C	3.476828	-3.466822	-1.365987
H	2.911211	-4.041245	-0.623734
H	4.266400	-4.118977	-1.754538
H	2.802668	-3.236324	-2.194818
C	3.024109	1.293945	0.104398
C	2.140752	2.236448	-0.477868
C	1.770554	3.378055	0.236996
H	1.079442	4.081575	-0.217326
C	2.240182	3.614858	1.515460
H	1.942468	4.507066	2.057792
C	3.078595	2.681960	2.103291
H	3.428413	2.849965	3.117634
C	3.478358	1.526270	1.431458
C	1.503566	2.049012	-1.841498
H	1.903453	1.128465	-2.257301
C	4.363827	0.566276	2.215120
H	4.695557	-0.214745	1.537106
C	-0.017936	1.894588	-1.719585
H	-0.299785	1.156960	-0.965187
H	-0.439646	1.575214	-2.678206

H	-0.497436	2.837075	-1.439443
C	1.826210	3.188731	-2.816379
H	1.380791	4.133301	-2.486432
H	1.414023	2.960386	-3.804988
H	2.901369	3.349690	-2.926088
C	3.577108	-0.106480	3.349536
H	3.250230	0.631792	4.089159
H	4.200981	-0.845815	3.863795
H	2.681492	-0.610790	2.977685
C	5.626643	1.235250	2.774602
H	5.387943	1.958921	3.559958
H	6.184855	1.759790	1.995577
H	6.285178	0.480394	3.217247

Table S2. Cartesian coordinates (x-y-z format) for all the proposed species in the calculated reaction mechanism, calculated at the ωB97X-D/6-31G(d,p) level.

1

E(scf) = –2459.00595300 a.u.

N	-2.992786	0.416767	0.085625
C	-2.552708	0.215383	-1.134404
B	0.757698	0.690089	0.054774
N	0.238837	3.172159	-0.148483
C	-3.388407	-0.128763	-2.227711
H	-2.947644	-0.250627	-3.211202
N	1.060656	2.495197	1.790408
C	-4.728451	-0.294743	-2.012610
H	-5.404021	-0.564010	-2.820636
C	-6.611252	-0.289276	-0.374331
H	-7.308189	-0.577813	-1.156704
C	-5.243835	-0.112194	-0.704233
C	-7.046399	-0.101898	0.912291
H	-8.094150	-0.242344	1.157939

1_{ad}

E(scf) = –2459.02522271 a.u.

N	1.725028	0.198766	-0.890622
C	1.829474	-0.688480	-1.872742
B	0.589728	0.030541	0.191351
N	2.361531	-0.813658	1.861806
C	2.804904	-0.617191	-2.872452
H	2.836112	-1.395466	-3.624674
N	0.377857	-0.320768	2.736925
C	3.734192	0.389256	-2.829869
H	4.536980	0.444587	-3.558505
C	4.520583	2.483166	-1.752878
H	5.336964	2.540332	-2.466705
C	3.631354	1.380437	-1.827926
C	4.344400	3.460101	-0.808399
H	5.029461	4.300100	-0.760573

C	-4.326068	0.258720	0.314560	C	2.578918	1.285432	-0.881186
C	-4.806026	0.455139	1.634331	C	2.372032	2.338722	0.036117
H	-4.081533	0.731922	2.392846	H	1.490447	2.293237	0.661105
C	-6.133178	0.276915	1.925923	C	3.242943	3.397613	0.074412
H	-6.492573	0.423389	2.939795	H	3.063656	4.208423	0.772724
C	1.092019	-2.107671	-0.674632	C	-1.865977	0.211392	-1.401037
H	-1.479536	0.322899	-1.279007	H	1.116572	-1.499140	-1.829452
C	1.334162	1.566485	2.836468	C	-0.892190	0.296304	2.939201
C	2.679211	1.258662	3.097330	C	-2.020036	-0.537472	2.985442
C	2.969133	0.389817	4.147689	C	-3.244942	0.023418	3.339886
H	4.006179	0.143248	4.359802	H	-4.123661	-0.614124	3.392190
C	1.950215	-0.173969	4.908487	C	-3.357839	1.381997	3.610732
H	2.190784	-0.848825	5.724255	H	-4.321118	1.807203	3.875071
C	0.626252	0.103349	4.602134	C	-2.242833	2.199603	3.506623
H	-0.172053	-0.369095	5.169290	H	-2.340283	3.268829	3.676142
C	0.290989	0.957779	3.548319	C	-0.993544	1.674993	3.169958
C	-1.165479	1.162346	3.196972	C	0.188059	2.610395	3.051880
H	-1.633544	0.175164	3.104034	H	-0.043574	3.348759	2.273683
H	-1.267707	1.602677	2.203244	H	1.063997	2.062410	2.699199
C	-1.918189	1.993453	4.241229	C	0.532541	3.331707	4.358779
H	-2.963576	2.137011	3.950896	H	1.389889	3.997429	4.220806
H	-1.465060	2.982723	4.363114	H	0.780937	2.616463	5.149462
H	-1.903848	1.507460	5.221866	H	-0.305493	3.936587	4.717015
C	3.789825	1.803979	2.227833	C	-1.931705	-1.998139	2.612758
H	3.522841	1.650585	1.176070	H	-1.378129	-2.081438	1.671464
H	4.687429	1.202075	2.411551	H	-2.943740	-2.352954	2.396855
C	4.130905	3.276161	2.472904	C	-1.318418	-2.914637	3.673885
H	5.011883	3.566864	1.892736	H	-1.394378	-3.960123	3.358905
H	4.343548	3.460812	3.530424	H	-1.834109	-2.810792	4.633968
H	3.307166	3.926899	2.171311	H	-0.259564	-2.695590	3.833214
C	0.683957	2.067680	0.534552	C	1.087170	-0.330138	1.551216
C	0.485474	4.388626	0.614071	C	2.455767	-1.183322	3.274338
H	-0.313267	5.120288	0.474920	H	3.412010	-0.878895	3.709361
H	1.439429	4.852388	0.319706	H	2.355449	-2.271888	3.393284
C	0.547866	3.837842	2.037234	C	1.270462	-0.447603	3.881974
H	1.214818	4.403475	2.692130	H	0.791720	-0.996994	4.696797

H	-0.453753	3.813128	2.490282	H	1.570278	0.537955	4.267645
C	-0.100233	3.161329	-1.529021	C	3.095278	-1.631531	0.946066
C	0.886461	2.924654	-2.502031	C	2.533719	-2.811155	0.415867
C	0.481447	2.895854	-3.838519	C	3.309370	-3.557583	-0.475249
H	1.214707	2.715322	-4.617153	H	2.903037	-4.468178	-0.902857
C	-0.846108	3.097960	-4.189051	C	4.596614	-3.166195	-0.814234
H	-1.137099	3.067657	-5.234860	H	5.179968	-3.764485	-1.507436
C	-1.804611	3.338451	-3.211495	C	5.145867	-2.019375	-0.255180
H	-2.839328	3.485512	-3.501434	H	6.158019	-1.731566	-0.517759
C	-1.449815	3.374346	-1.864600	C	4.410378	-1.240080	0.636285
C	-2.467752	3.602553	-0.765666	C	4.991064	0.002204	1.279605
H	-2.288043	4.584265	-0.306976	H	5.140806	-0.194165	2.349708
H	-2.284682	2.861990	0.019747	H	4.239775	0.795810	1.237241
C	-3.931321	3.525406	-1.190198	C	6.305970	0.503314	0.689307
H	-4.578441	3.598734	-0.312221	H	6.588460	1.449891	1.157204
H	-4.150151	2.573682	-1.682826	H	6.213116	0.679664	-0.386194
H	-4.205176	4.334118	-1.875522	H	7.124313	-0.205469	0.850959
C	2.327690	2.676537	-2.110343	C	1.127652	-3.254911	0.766623
H	2.436020	1.613643	-1.857224	H	0.415993	-2.734362	0.111071
H	2.537336	3.208100	-1.176044	H	0.876242	-2.892744	1.766479
C	3.372804	3.069121	-3.151851	C	0.867816	-4.758348	0.712372
H	4.374280	2.928058	-2.736045	H	-0.148293	-4.959873	1.062011
H	3.268469	4.115278	-3.457300	H	1.570528	-5.314186	1.341280
H	3.312878	2.449063	-4.051850	H	0.934356	-5.157227	-0.304429
N	0.297777	-3.184327	-0.384322	N	-2.428331	1.433666	-1.856409
C	-1.033593	-3.070695	0.111628	C	-1.816972	2.640074	-1.412928
C	-2.118367	-3.224701	-0.762673	C	-0.622213	3.129539	-1.964927
C	-3.412053	-3.132228	-0.245831	C	-0.061020	4.291714	-1.428213
H	-4.257458	-3.234111	-0.920925	H	0.866253	4.670864	-1.851540
C	-3.625064	-2.861908	1.096045	C	-0.647960	4.938456	-0.350253
H	-4.635973	-2.757054	1.477702	H	-0.194523	5.835669	0.061124
C	-2.537535	-2.672841	1.940737	C	-1.811335	4.422238	0.212330
H	-2.703890	-2.432995	2.987471	H	-2.262797	4.911537	1.071715
C	-1.228725	-2.773647	1.472623	C	-2.405050	3.270844	-0.301164
C	-0.075553	-2.527958	2.417774	C	-3.629938	2.685642	0.359589
H	-0.484412	-2.117844	3.346432	H	-3.776171	3.190602	1.319626

H	0.567608	-1.744395	2.001410	H	-3.422173	1.636065	0.594182
C	0.760355	-3.764389	2.757825	C	-4.917016	2.796559	-0.460915
H	1.533176	-3.502641	3.487241	H	-5.763970	2.388713	0.100378
H	1.263010	-4.163963	1.872745	H	-4.832252	2.236171	-1.394293
H	0.141561	-4.560461	3.183983	H	-5.142279	3.840105	-0.704071
C	-1.935355	-3.465214	-2.242502	C	0.082847	2.442127	-3.111747
H	-0.929500	-3.163967	-2.549507	H	-0.201961	1.387432	-3.141966
H	-2.627558	-2.809617	-2.781710	H	1.160907	2.461812	-2.914148
C	-2.212177	-4.918056	-2.647251	C	-0.178279	3.120866	-4.461030
H	-2.050583	-5.066509	-3.719041	H	0.333973	2.594800	-5.272698
H	-3.246853	-5.191531	-2.419179	H	0.177652	4.155913	-4.448931
H	-1.565381	-5.613870	-2.104141	H	-1.247463	3.146072	-4.692009
N	2.272480	-2.595180	-1.192803	N	-2.508957	-0.799833	-2.169648
C	2.377879	-4.030898	-0.968975	C	-3.517423	-0.203685	-3.033931
H	2.926843	-4.257677	-0.043146	H	-4.521890	-0.188343	-2.583353
H	2.883814	-4.523168	-1.803589	H	-3.578626	-0.731118	-3.991795
C	0.902211	-4.426522	-0.851750	C	-2.980930	1.221779	-3.181353
H	0.500598	-4.733904	-1.826481	H	-2.232313	1.254822	-3.988582
H	0.731672	-5.234509	-0.135237	H	-3.763729	1.953262	-3.404382
C	3.396902	-1.728715	-1.335306	C	-2.689773	-2.081739	-1.581698
C	3.684279	-1.226438	-2.612880	C	-1.879075	-3.138139	-2.038102
C	4.738681	-0.323648	-2.750172	C	-2.010256	-4.391895	-1.439625
H	4.963701	0.082075	-3.732965	H	-1.388531	-5.218206	-1.769672
C	5.482956	0.073433	-1.646414	C	-2.930396	-4.600338	-0.418768
H	6.295101	0.784263	-1.766093	H	-3.022817	-5.581797	0.036772
C	5.181637	-0.431982	-0.388307	C	-3.729927	-3.552653	0.016610
H	5.754620	-0.110022	0.477455	H	-4.448886	-3.715807	0.816366
C	4.132768	-1.333918	-0.207263	C	-3.615411	-2.279496	-0.542737
C	3.822488	-1.840965	1.181908	C	-4.485833	-1.160189	-0.018371
H	2.829360	-2.291962	1.211718	H	-4.128372	-0.200051	-0.391289
H	3.767898	-0.985448	1.861025	H	-4.373911	-1.110739	1.069702
C	4.865280	-2.836403	1.700172	C	-5.966774	-1.342078	-0.365973
H	4.605650	-3.178419	2.706527	H	-6.558325	-0.505037	0.017005
H	5.860182	-2.382312	1.742605	H	-6.368923	-2.265428	0.063161
H	4.935088	-3.714498	1.049645	H	-6.114944	-1.394650	-1.449242
C	2.834122	-1.579689	-3.810076	C	-0.906176	-2.896821	-3.173482

H	2.368976	-2.557359	-3.657794
H	3.477880	-1.658073	-4.693702
C	1.741301	-0.531591	-4.059156
H	1.115070	-0.813163	-4.911584
H	2.188604	0.443642	-4.274501
H	1.107579	-0.414862	-3.174315
B	0.825176	-0.686096	-0.447711

H	-0.652331	-1.833323	-3.168540
H	-1.424674	-3.065556	-4.126934
C	0.362076	-3.750085	-3.142683
H	1.048986	-3.440018	-3.936673
H	0.153998	-4.813269	-3.295536
H	0.886277	-3.651672	-2.186039
B	-0.876948	0.088712	-0.376505

$TS_{1ad \rightarrow A1}$

E(scf) = –2458.99526390 a.u.

ν_{min} = –890.7 cm^{-1}

N	1.551073	1.100594	-0.039488
C	0.576050	1.598641	0.816352
B	-0.574645	0.663412	0.348853
N	-2.686376	0.872942	1.851332
C	0.919976	2.702624	1.674276
H	0.186284	3.080636	2.379235
N	-2.620274	2.179159	0.102650
C	2.165834	3.241047	1.610653
H	2.440353	4.066565	2.262733
C	4.352851	3.433198	0.421767
H	4.641139	4.240022	1.091535
C	3.119979	2.791760	0.634128
C	5.169577	3.080552	-0.633939
H	6.110706	3.595395	-0.796902
C	2.754471	1.714778	-0.213234
C	3.569834	1.394527	-1.313268
H	3.222719	0.627282	-1.996227
C	4.749433	2.075453	-1.522199
H	5.359453	1.830955	-2.387096
C	1.206420	-1.314449	-0.993428
H	0.140748	0.538650	1.555300
C	-2.113967	2.851227	-1.056280
C	-2.220993	2.222056	-2.305761
C	-1.727951	2.902761	-3.417256

A1

E(scf) = –2459.06040238 a.u.

N	-1.352831	0.768069	-0.248862
C	-0.478167	1.547227	-0.943761
B	0.839589	0.611765	-0.669718
N	3.083519	1.780590	-0.092177
C	-0.944847	2.714079	-1.543592
H	-0.268698	3.310942	-2.146533
N	1.510854	2.229088	1.341519
C	-2.268743	3.103194	-1.379373
H	-2.656410	3.988140	-1.875312
C	-4.411982	2.801753	-0.123768
H	-4.828646	3.689379	-0.591656
C	-3.104457	2.385874	-0.494070
C	-5.124522	2.118470	0.828999
H	-6.114374	2.463708	1.112392
C	-2.607049	1.200081	0.105347
C	-3.333476	0.523609	1.098989
H	-2.862348	-0.330488	1.575572
C	-4.571976	0.985134	1.470202
H	-5.127146	0.483745	2.256380
C	-0.948591	-1.781798	-0.252413
H	1.467620	0.249864	-1.638396
C	0.333691	2.140283	2.148540
C	0.184203	1.040462	3.010830
C	-0.947608	1.005247	3.823682
H	-1.087303	0.158443	4.489794

H	-1.792844	2.429421	-4.392566	C	-1.897466	2.019455	3.787040
C	-1.142971	4.157042	-3.293474	H	-2.782556	1.958892	4.411985
H	-0.752002	4.662143	-4.170823	C	-1.721295	3.098651	2.937775
C	-1.040521	4.752440	-2.046196	H	-2.471586	3.882618	2.890236
H	-0.560948	5.721740	-1.943612	C	-0.598651	3.185149	2.111957
C	-1.524053	4.112328	-0.904128	C	-0.430488	4.413891	1.249616
C	-1.385483	4.802201	0.433523	H	-1.364635	4.579457	0.704724
H	-0.332265	5.062686	0.578803	H	0.329005	4.239068	0.483215
H	-1.626727	4.109421	1.243669	C	-0.094428	5.665148	2.069744
C	-2.250311	6.062679	0.545039	H	0.020441	6.537473	1.419720
H	-2.132913	6.530760	1.526556	H	0.832154	5.539696	2.639365
H	-3.311870	5.836964	0.397736	H	-0.887338	5.883585	2.791001
H	-1.970655	6.799176	-0.213439	C	1.171786	-0.101935	3.065772
C	-2.795079	0.836806	-2.476222	H	1.257218	-0.542143	2.067639
H	-2.241072	0.139332	-1.839181	H	0.732895	-0.880822	3.693639
H	-2.584879	0.519995	-3.499986	C	2.565714	0.209965	3.615072
C	-4.298258	0.693938	-2.233614	H	3.109199	-0.728357	3.760674
H	-4.613846	-0.317925	-2.503701	H	2.517145	0.733936	4.575213
H	-4.872906	1.408018	-2.831772	H	3.155418	0.814429	2.921988
H	-4.558174	0.842074	-1.183102	C	1.807703	1.505540	0.243844
C	-1.992031	1.185371	0.746578	C	3.693335	2.823139	0.734426
C	-3.811012	1.786209	2.079801	H	3.940208	3.688362	0.110356
H	-3.607436	2.404427	2.961007	H	4.619039	2.466051	1.195500
H	-4.732256	1.227658	2.261901	C	2.593299	3.126275	1.755314
C	-3.850641	2.602104	0.778679	H	2.887779	2.913509	2.786302
H	-4.723584	2.376789	0.158340	H	2.264329	4.167091	1.705621
H	-3.833700	3.678080	0.961369	C	3.843035	1.235094	-1.173588
C	-2.542571	-0.323699	2.623378	C	4.731325	0.187860	-0.877860
C	-3.264371	-1.451111	2.195124	C	5.543288	-0.288588	-1.903971
C	-3.176597	-2.607842	2.965068	H	6.242051	-1.094879	-1.712026
H	-3.714461	-3.500036	2.664119	C	5.476744	0.265265	-3.178442
C	-2.400329	-2.634621	4.119055	H	6.122422	-0.112829	-3.964836
H	-2.338896	-3.546105	4.705077	C	4.593915	1.299256	-3.444762
C	-1.706025	-1.505312	4.524888	H	4.548902	1.729385	-4.441174
H	-1.107995	-1.537839	5.430469	C	3.757294	1.805090	-2.447122
C	-1.765686	-0.321754	3.785956	C	2.786686	2.914828	-2.782088

C	-1.018259	0.905682	4.255207	H	3.351997	3.784361	-3.139529
H	-1.459843	1.242638	5.201543	H	2.253509	3.231328	-1.881045
H	-1.167461	1.715151	3.535797	C	1.766211	2.495896	-3.846090
C	0.483751	0.679361	4.448433	H	1.094697	3.325184	-4.087934
H	0.969238	1.601571	4.777888	H	1.167071	1.658711	-3.480935
H	0.958901	0.369785	3.514404	H	2.259354	2.187664	-4.772322
H	0.680654	-0.090690	5.200482	C	4.764163	-0.399568	0.517321
C	-4.071509	-1.391200	0.914331	H	3.800542	-0.884453	0.714989
H	-3.377827	-1.425447	0.064049	H	4.829972	0.409587	1.252138
H	-4.562900	-0.415928	0.839515	C	5.897416	-1.381273	0.792909
C	-5.138675	-2.467914	0.744967	H	5.835830	-1.738061	1.824255
H	-5.685131	-2.298998	-0.186559	H	6.876646	-0.912760	0.651996
H	-5.855598	-2.453920	1.571681	H	5.848786	-2.257678	0.139014
H	-4.710389	-3.472581	0.689543	N	-0.167835	-2.952975	-0.140118
N	0.533903	-2.431749	-1.543618	C	0.830554	-3.091377	0.866555
C	-0.749801	-2.386859	-2.155019	C	0.481344	-3.254684	2.218369
C	-0.953649	-1.834729	-3.430182	C	1.495952	-3.513048	3.142493
C	-2.199262	-2.011866	-4.039690	H	1.238693	-3.632366	4.192271
H	-2.363461	-1.594902	-5.030711	C	2.821075	-3.613910	2.742879
C	-3.227875	-2.686250	-3.398874	H	3.596084	-3.832304	3.471550
H	-4.186635	-2.816535	-3.892024	C	3.151786	-3.422411	1.406553
C	-3.028478	-3.182013	-2.114914	H	4.188442	-3.492506	1.092740
H	-3.835825	-3.696880	-1.600766	C	2.172127	-3.138502	0.454828
C	-1.795703	-3.039290	-1.478597	C	2.560048	-2.849971	-0.978237
C	-1.599410	-3.574930	-0.078797	H	3.630684	-2.614929	-1.002312
H	-2.587117	-3.741640	0.361469	H	2.034299	-1.948410	-1.308302
H	-1.111289	-2.806949	0.529060	C	2.287176	-3.988771	-1.964458
C	-0.809996	-4.882802	0.015558	H	2.718564	-3.755239	-2.942453
H	-0.835640	-5.267576	1.039972	H	1.213755	-4.134396	-2.101234
H	0.236705	-4.730245	-0.253835	H	2.723976	-4.930454	-1.616146
H	-1.230073	-5.646664	-0.646619	C	-0.942884	-3.126628	2.718348
C	0.106225	-1.033683	-4.154131	H	-1.597891	-2.781884	1.916574
H	0.946404	-0.839397	-3.485445	H	-0.967075	-2.332460	3.475168
H	-0.316125	-0.044290	-4.372203	C	-1.492924	-4.416807	3.336217
C	0.580481	-1.676823	-5.461082	H	-2.520563	-4.271566	3.682020
H	1.338211	-1.052920	-5.943889	H	-0.893183	-4.739903	4.192565

H	-0.243926	-1.810006	-6.168691
H	1.018822	-2.663629	-5.282817
N	2.543537	-1.764944	-0.783663
C	2.710899	-3.131875	-1.279032
H	2.682026	-3.850276	-0.446029
H	3.656129	-3.267407	-1.813174
C	1.509265	-3.302299	-2.186731
H	1.749359	-2.974871	-3.210098
H	1.144748	-4.332145	-2.236815
C	3.241918	-1.462619	0.427629
C	4.610618	-1.129993	0.346496
C	5.267987	-0.706922	1.500644
H	6.307813	-0.404981	1.444609
C	4.609467	-0.653688	2.720328
H	5.126490	-0.295622	3.605251
C	3.304134	-1.113652	2.812295
H	2.823117	-1.157909	3.784484
C	2.609960	-1.559331	1.686398
C	1.253800	-2.214174	1.854837
H	1.027735	-2.787740	0.955032
H	0.465338	-1.455821	1.909266
C	1.152974	-3.146995	3.062736
H	0.188012	-3.663397	3.047296
H	1.210017	-2.607257	4.013290
H	1.948975	-3.898307	3.055010
C	5.365118	-1.297747	-0.955982
H	4.731480	-0.948324	-1.775093
H	5.504342	-2.373387	-1.130747
C	6.736227	-0.630574	-1.026943
H	7.152600	-0.732994	-2.033492
H	7.447024	-1.089457	-0.332494
H	6.669631	0.434779	-0.791576
B	0.663799	0.012642	-0.646334

H	-1.492144	-5.236219	2.611086
N	-2.177667	-2.209470	-0.825726
C	-2.055630	-3.587259	-1.297675
H	-1.744634	-3.616082	-2.353120
H	-3.002622	-4.127877	-1.206286
C	-0.970354	-4.141572	-0.399922
H	-1.402160	-4.559065	0.520832
H	-0.377049	-4.927246	-0.877211
C	-2.947917	-1.355956	-1.676589
C	-4.320013	-1.224354	-1.388649
C	-5.083523	-0.343872	-2.152143
H	-6.135555	-0.205657	-1.929017
C	-4.506840	0.383935	-3.186333
H	-5.106967	1.087461	-3.755210
C	-3.170918	0.192818	-3.501249
H	-2.724613	0.736573	-4.330161
C	-2.375925	-0.697849	-2.777819
C	-0.962225	-0.944278	-3.259195
H	-0.380210	-1.481836	-2.511751
H	-0.463354	0.022859	-3.381901
C	-0.929195	-1.708671	-4.586873
H	0.103661	-1.868209	-4.911295
H	-1.455044	-1.167249	-5.379706
H	-1.408484	-2.688520	-4.487938
C	-4.929179	-2.048864	-0.273199
H	-4.291845	-1.956291	0.610627
H	-4.876019	-3.109545	-0.552205
C	-6.372698	-1.715232	0.088384
H	-6.696749	-2.319792	0.940473
H	-7.057626	-1.920811	-0.740401
H	-6.478665	-0.660985	0.360667
B	-0.470900	-0.405220	-0.013679

TS$_{A1 \rightarrow A2}$

A2

E(scf) = –2459.03680426 a.u.

ν_{min} = –126.2 cm^{-1}

N	-1.761088	-0.445516	0.255123
C	-0.966062	-1.011429	1.162344
B	0.547342	-0.660353	0.699916
N	2.379121	-2.493642	0.517580
C	-1.537320	-1.750881	2.230063
H	-0.890022	-2.153599	3.002193
N	0.658109	-2.912516	-0.743824
C	-2.894141	-1.942679	2.274507
H	-3.355101	-2.492092	3.092033
C	-5.091585	-1.775782	1.098004
H	-5.571595	-2.353776	1.883143
C	-3.710360	-1.477789	1.212760
C	-5.801080	-1.361318	0.000643
H	-6.854112	-1.607215	-0.092291
C	-3.092419	-0.706504	0.196978
C	-3.844440	-0.273339	-0.917827
H	-3.327229	0.288049	-1.687528
C	-5.166112	-0.617403	-1.022320
H	-5.735195	-0.320268	-1.897662
C	-0.309357	1.992759	-0.294883
H	1.288207	-0.278203	1.580532
C	-0.558665	-2.804335	-1.491322
C	-0.571414	-2.008905	-2.648483
C	-1.747722	-1.970887	-3.396020
H	-1.780423	-1.351415	-4.287696
C	-2.869834	-2.695717	-3.015557
H	-3.781623	-2.635409	-3.600920
C	-2.832645	-3.474256	-1.869858
H	-3.718311	-4.016860	-1.551287
C	-1.676886	-3.547978	-1.091423
C	-1.686212	-4.431880	0.134587
H	-2.586005	-4.201232	0.713177
H	-0.844999	-4.186875	0.788661
C	-1.684775	-5.925126	-0.213500

E(scf) = –2459.04768183 a.u.

N	-2.187396	-1.292982	0.086364
C	-1.735742	-0.289996	-0.655277
B	-0.550850	0.620623	-0.056087
N	-2.039261	1.839188	1.679607
C	-2.256607	-0.058759	-1.969491
H	-1.882321	0.792148	-2.528925
N	-0.418491	0.709918	2.585738
C	-3.212780	-0.877728	-2.491353
H	-3.626921	-0.703894	-3.481714
C	-4.655198	-2.896475	-2.181591
H	-5.072839	-2.765170	-3.176564
C	-3.677653	-1.980910	-1.724656
C	-5.068663	-3.933295	-1.382149
H	-5.817807	-4.632724	-1.739181
C	-3.125709	-2.139893	-0.427118
C	-3.568352	-3.220027	0.376678
H	-3.124468	-3.328547	1.361330
C	-4.516751	-4.095735	-0.090857
H	-4.847336	-4.921697	0.531607
C	1.905420	-0.483005	-1.330884
H	-0.421163	1.627020	-0.737172
C	0.804188	0.002065	2.814065
C	2.001528	0.737000	2.822693
C	3.155617	0.086372	3.248166
H	4.094544	0.632810	3.265305
C	3.124379	-1.250772	3.631840
H	4.035396	-1.740465	3.961382
C	1.943730	-1.970404	3.540742
H	1.932188	-3.030811	3.776493
C	0.758533	-1.360217	3.122367
C	-0.499375	-2.183257	2.979095
H	-0.249871	-3.053716	2.361404
H	-1.249853	-1.637048	2.401487
C	-1.070121	-2.661942	4.317941
H	-1.974605	-3.257391	4.161460

H	-1.679731	-6.536956	0.693383	H	-1.329055	-1.822953	4.972744
H	-0.813284	-6.199918	-0.816325	H	-0.349707	-3.282906	4.859820
H	-2.573702	-6.189112	-0.793919	C	2.076622	2.157732	2.311916
C	0.626940	-1.211498	-3.107577	H	1.591010	2.203865	1.330307
H	1.042837	-0.658565	-2.260694	H	3.129198	2.387893	2.122927
H	0.265433	-0.445403	-3.799931	C	1.509932	3.238611	3.235030
C	1.709804	-2.036796	-3.808057	H	1.701960	4.229019	2.811620
H	2.504213	-1.379484	-4.175135	H	1.970237	3.195875	4.227031
H	1.304317	-2.591765	-4.659735	H	0.428476	3.145317	3.360225
H	2.176070	-2.757554	-3.129431	C	-0.967636	1.072983	1.428284
C	1.188531	-2.017947	0.110644	C	-2.229887	2.092118	3.109040
C	2.764495	-3.731495	-0.163377	H	-3.263325	1.900258	3.403906
H	3.119847	-4.468616	0.560329	H	-1.993089	3.138257	3.339021
H	3.572815	-3.531069	-0.877325	C	-1.241577	1.104863	3.736143
C	1.460509	-4.136995	-0.846975	H	-0.621305	1.544367	4.519609
H	1.585068	-4.425359	-1.892630	H	-1.743342	0.220199	4.141501
H	0.963239	-4.958155	-0.321287	C	-2.824999	2.509170	0.690035
C	3.403940	-1.748077	1.181360	C	-2.306398	3.666333	0.086113
C	4.236188	-0.935402	0.399346	C	-3.092361	4.294072	-0.879166
C	5.300550	-0.298832	1.037728	H	-2.726915	5.185476	-1.376269
H	5.961728	0.337278	0.460177	C	-4.344274	3.791902	-1.214710
C	5.517244	-0.459920	2.400382	H	-4.938082	4.292486	-1.973207
H	6.352825	0.042928	2.877387	C	-4.843829	2.661779	-0.583257
C	4.669224	-1.261525	3.153178	H	-5.823336	2.285035	-0.855779
H	4.836835	-1.381834	4.220001	C	-4.095230	1.998776	0.389552
C	3.598129	-1.923955	2.554980	C	-4.623709	0.776754	1.111859
C	2.658040	-2.750578	3.400016	H	-4.985657	1.085221	2.103150
H	3.244692	-3.415945	4.043906	H	-3.790135	0.091298	1.287846
H	2.040226	-3.385508	2.758304	C	-5.741706	0.024550	0.392729
C	1.748709	-1.869160	4.265457	H	-5.937859	-0.924588	0.897946
H	1.066429	-2.481853	4.863189	H	-5.455819	-0.206582	-0.636323
H	1.163308	-1.194590	3.635288	H	-6.676859	0.593054	0.373149
H	2.339373	-1.254388	4.951134	C	-0.934860	4.185875	0.468078
C	3.965401	-0.714051	-1.072072	H	-0.179609	3.482497	0.097613
H	3.487686	0.263625	-1.182525	H	-0.840545	4.172367	1.560377
H	3.228078	-1.432891	-1.433119	C	-0.587361	5.580035	-0.042347

C	5.199478	-0.789151	-1.969094
H	4.912153	-0.636264	-3.013145
H	5.693871	-1.762840	-1.885357
H	5.930867	-0.015992	-1.721666
N	0.771705	2.925697	-0.389270
C	2.085452	2.634096	-0.854159
C	2.387947	2.508703	-2.223222
C	3.730452	2.495986	-2.614604
H	3.969707	2.407665	-3.671739
C	4.752338	2.607328	-1.681942
H	5.788290	2.630076	-2.007502
C	4.441320	2.651019	-0.328110
H	5.234239	2.697760	0.413322
C	3.115393	2.632662	0.104398
C	2.804413	2.618428	1.582154
H	3.673759	2.204520	2.106252
H	1.977851	1.924819	1.758060
C	2.461330	3.992381	2.162892
H	2.331307	3.931207	3.247690
H	1.528002	4.365988	1.734187
H	3.252936	4.719820	1.954945
C	1.319438	2.394177	-3.289369
H	0.345684	2.233477	-2.820240
H	1.526425	1.484772	-3.866801
C	1.285638	3.584055	-4.254975
H	0.500887	3.452102	-5.005989
H	2.238879	3.695375	-4.781142
H	1.095219	4.523620	-3.728326
N	-1.471400	2.813883	-0.122265
C	-1.082909	4.220874	-0.053000
H	-0.953452	4.546549	0.991141
H	-1.824856	4.874340	-0.524334
C	0.243566	4.224055	-0.778202
H	0.083551	4.322957	-1.862920
H	0.913047	5.026088	-0.453258
C	-2.522932	2.460264	0.775371

H	0.405503	5.864619	0.315641
H	-1.305366	6.332192	0.299613
H	-0.555860	5.609453	-1.135619
N	2.453543	-1.768705	-1.529148
C	1.821759	-2.878619	-0.890978
C	0.576600	-3.377329	-1.306019
C	-0.003417	-4.420473	-0.578558
H	-0.979902	-4.790300	-0.881582
C	0.637870	-4.967980	0.522472
H	0.173851	-5.779201	1.075740
C	1.870467	-4.463283	0.925229
H	2.370817	-4.881853	1.794785
C	2.470378	-3.408694	0.239262
C	3.780520	-2.834243	0.724335
H	3.965316	-3.214346	1.734375
H	3.672791	-1.748843	0.816859
C	4.988636	-3.162022	-0.156119
H	5.910304	-2.794129	0.306164
H	4.896063	-2.689378	-1.135876
H	5.090243	-4.241938	-0.303054
C	-0.147157	-2.859241	-2.525036
H	0.168869	-1.838177	-2.749420
H	-1.213244	-2.801165	-2.290916
C	0.036610	-3.771812	-3.742973
H	-0.495460	-3.371493	-4.611353
H	-0.352837	-4.774215	-3.539321
H	1.092806	-3.879493	-4.009369
N	2.570742	0.369260	-2.252554
C	3.554045	-0.393756	-3.007970
H	4.566630	-0.346302	-2.580139
H	3.602819	-0.053039	-4.047285
C	2.991003	-1.810401	-2.881990
H	2.221772	-1.978368	-3.650760
H	3.754515	-2.587211	-2.978024
C	2.763214	1.726074	-1.865592
C	1.887733	2.689520	-2.399301

C	-3.840864	2.609378	0.298916
C	-4.904644	2.247113	1.122972
H	-5.922637	2.328533	0.757474
C	-4.677802	1.738330	2.395153
H	-5.513473	1.424676	3.013250
C	-3.379497	1.644858	2.873554
H	-3.197430	1.273434	3.879421
C	-2.287693	2.033586	2.095125
C	-0.917651	2.026946	2.737662
H	-0.142348	2.155010	1.985812
H	-0.743293	1.043680	3.190974
C	-0.776214	3.113440	3.808859
H	0.210756	3.062296	4.278818
H	-1.533228	3.011635	4.593137
H	-0.889197	4.109896	3.369348
C	-4.062635	3.142838	-1.101465
H	-3.362421	2.632519	-1.771022
H	-3.759210	4.197345	-1.133019
C	-5.486918	3.044252	-1.639736
H	-5.524273	3.395461	-2.674909
H	-6.181906	3.659517	-1.059415
H	-5.854764	2.014549	-1.616989
B	-0.154148	0.554045	-0.319577

C	2.023052	4.014826	-1.985295
H	1.359097	4.774546	-2.383819
C	2.993562	4.379311	-1.058655
H	3.084165	5.416216	-0.748348
C	3.836762	3.415146	-0.524477
H	4.582279	3.695103	0.216515
C	3.726251	2.076320	-0.903454
C	4.623954	1.049809	-0.249597
H	4.238296	0.044573	-0.426674
H	4.583586	1.195218	0.836039
C	6.082028	1.148701	-0.710730
H	6.696010	0.385264	-0.223508
H	6.507124	2.129204	-0.473945
H	6.164850	1.011062	-1.793261
C	0.818699	2.267113	-3.386945
H	0.525784	1.242838	-3.143005
H	1.261281	2.219771	-4.390960
C	-0.423357	3.155789	-3.418792
H	-1.170043	2.735049	-4.099147
H	-0.206732	4.170580	-3.768237
H	-0.880549	3.225200	-2.426129
B	0.918810	-0.039450	-0.397940

TS$_{A2 \rightarrow A3}$

E(scf) = –2459.04306483 a.u.

ν_{min} = –61.8 cm^{-1}

N	2.321011	-1.490416	0.788219
C	1.621014	-0.372988	0.651687
B	0.225818	-0.457760	-0.145240
N	1.291620	-0.391420	-2.503560
C	2.106063	0.859936	1.183740
H	1.502247	1.744981	1.030201
N	-0.359461	-1.801276	-2.315969
C	3.309972	0.923099	1.819116

A3

E(scf) = –2459.05398081 a.u.

N	-2.507137	1.399459	0.460386
C	-1.750124	0.316391	0.556646
B	-0.380026	0.282544	-0.261298
N	-1.352549	-0.212470	-2.571852
C	-2.150071	-0.797798	1.353276
H	-1.483135	-1.649862	1.393877
N	0.300655	1.211318	-2.612941
C	-3.346599	-0.790351	2.005314
H	-3.678160	-1.646890	2.587473

H	3.699906	1.864698	2.198420	C	-5.451611	0.456323	2.518860
C	5.354889	-0.303248	2.571772	H	-5.817509	-0.381009	3.107438
H	5.776387	0.616541	2.969202	C	-4.179322	0.355473	1.904639
C	4.077326	-0.262611	1.961591	C	-6.210014	1.591077	2.372527
C	6.049836	-1.483868	2.659220	H	-7.185214	1.659373	2.844120
H	7.029004	-1.506952	3.126891	C	-3.697516	1.440589	1.127824
C	3.522525	-1.456704	1.434664	C	-4.495417	2.605364	1.003077
C	4.256586	-2.664169	1.547601	H	-4.101654	3.421305	0.405297
H	3.808454	-3.563934	1.137828	C	-5.723820	2.677997	1.609391
C	5.491879	-2.676940	2.144105	H	-6.331722	3.571102	1.502851
H	6.048854	-3.605609	2.221528	C	1.777423	-0.664415	1.429769
C	-1.787509	1.189429	1.211847	H	0.541000	1.082396	0.186968
H	-0.567847	-1.236229	0.408320	C	1.207458	2.242556	-2.219795
C	-1.280933	-2.746946	-1.764274	C	2.579735	2.009102	-2.364764
C	-2.645861	-2.560987	-2.010155	C	3.453063	3.077619	-2.156419
C	-3.531617	-3.566657	-1.619273	H	4.521561	2.916215	-2.270919
H	-4.594283	-3.435547	-1.804902	C	2.974192	4.322413	-1.779601
C	-3.070697	-4.710677	-0.988124	H	3.664353	5.144789	-1.617817
H	-3.768725	-5.486655	-0.689273	C	1.612772	4.507240	-1.567914
C	-1.716687	-4.847851	-0.706228	H	1.247647	5.469690	-1.222153
H	-1.364130	-5.727556	-0.175980	C	0.698382	3.477047	-1.778967
C	-0.791316	-3.875081	-1.081246	C	-0.766611	3.707156	-1.474861
C	0.660331	-4.047819	-0.692777	H	-0.847071	4.690550	-1.000455
H	0.716601	-4.926473	-0.041705	H	-1.116050	2.984194	-0.728114
H	0.990987	-3.198581	-0.082723	C	-1.721250	3.666154	-2.673488
C	1.656682	-4.233837	-1.842652	H	-2.710679	4.020281	-2.370069
H	2.629469	-4.532890	-1.441578	H	-1.856256	2.650074	-3.053151
H	1.820897	-3.304898	-2.394408	H	-1.370249	4.300377	-3.494176
H	1.328297	-5.004428	-2.547992	C	3.136096	0.643240	-2.694455
C	-3.182117	-1.305100	-2.653751	H	2.350938	-0.111031	-2.594767
H	-2.386896	-0.560219	-2.726576	H	3.872342	0.393275	-1.922277
H	-3.925516	-0.871759	-1.975895	C	3.806346	0.558085	-4.068797
C	-3.824206	-1.536949	-4.024608	H	4.195090	-0.449227	-4.244517
H	-4.209513	-0.598498	-4.433770	H	4.642523	1.259649	-4.146105
H	-4.657315	-2.243189	-3.959171	H	3.107991	0.798759	-4.876930
H	-3.106524	-1.950259	-4.741040	C	-0.484221	0.480296	-1.815139

C	0.393498	-0.916328	-1.654151	C	-1.096939	-0.062875	-4.004332
C	1.094733	-0.834035	-3.884444	H	-1.999576	0.266974	-4.526720
H	2.017693	-1.254198	-4.293725	H	-0.784929	-1.023470	-4.427374
H	0.798538	0.017527	-4.506255	C	0.028371	0.983325	-4.035832
C	-0.025565	-1.875348	-3.742927	H	0.924939	0.615856	-4.541888
H	-0.900722	-1.632237	-4.351443	H	-0.274683	1.921042	-4.510445
H	0.298311	-2.889012	-3.995092	C	-2.201249	-1.245035	-2.061364
C	2.182465	0.681016	-2.180839	C	-1.640364	-2.486054	-1.745815
C	1.681774	1.984430	-2.119265	C	-2.477302	-3.452474	-1.181628
C	2.558392	3.001599	-1.732149	H	-2.053237	-4.413507	-0.902045
H	2.175551	4.014450	-1.639546	C	-3.820776	-3.188700	-0.966510
C	3.887062	2.723370	-1.454382	H	-4.456241	-3.946233	-0.518001
H	4.555625	3.522357	-1.148414	C	-4.362230	-1.955356	-1.319930
C	4.372457	1.422642	-1.563200	H	-5.413635	-1.764247	-1.138415
H	5.413654	1.222625	-1.337995	C	-3.563929	-0.958239	-1.872539
C	3.530529	0.375111	-1.923558	C	-4.102372	0.410374	-2.232493
C	4.008361	-1.059250	-2.004754	H	-3.991665	0.562477	-3.314900
H	3.888205	-1.417844	-3.036159	H	-3.462093	1.158820	-1.753225
H	3.337608	-1.670485	-1.391530	C	-5.553273	0.670455	-1.839600
C	5.447687	-1.302633	-1.561478	H	-5.827929	1.697915	-2.091596
H	5.674623	-2.370459	-1.614079	H	-5.700679	0.547604	-0.762406
H	5.604713	-0.985863	-0.525966	H	-6.245236	0.002066	-2.362390
H	6.166126	-0.776167	-2.198276	C	-0.190232	-2.820002	-1.994606
C	0.253481	2.325811	-2.461845	H	0.230551	-3.219202	-1.070187
H	-0.190031	2.855908	-1.615971	H	0.387064	-1.919396	-2.210430
H	-0.338697	1.417252	-2.584437	C	-0.004886	-3.842490	-3.118773
C	0.136416	3.191064	-3.718949	H	1.057186	-4.064526	-3.256184
H	-0.914760	3.405477	-3.931959	H	-0.407741	-3.473798	-4.068735
H	0.571122	2.690300	-4.590822	H	-0.514048	-4.782974	-2.887193
H	0.651151	4.147864	-3.591806	N	2.450178	0.216347	2.306383
N	-2.587394	0.534203	2.179980	C	2.102093	1.597789	2.318440
C	-2.370567	-0.853524	2.428341	C	0.894854	2.060179	2.866080
C	-1.246318	-1.321150	3.126861	C	0.616018	3.427480	2.801934
C	-1.095062	-2.699489	3.293366	H	-0.323219	3.790721	3.211588
H	-0.218831	-3.071402	3.818459	C	1.504966	4.314006	2.211831
C	-2.022414	-3.591123	2.774545	H	1.269440	5.373275	2.168468

H	-1.882676	-4.659751	2.907123	C	2.686862	3.839295	1.655589
C	-3.113554	-3.114496	2.058151	H	3.372005	4.525896	1.167002
H	-3.822520	-3.810504	1.620436	C	2.991764	2.480460	1.683332
C	-3.291060	-1.747925	1.859004	C	4.248773	1.965596	1.023139
C	-4.439147	-1.237776	1.020561	H	4.587286	2.715236	0.301168
H	-4.786804	-2.056149	0.380591	H	4.000062	1.062294	0.456103
H	-4.064141	-0.448721	0.359820	C	5.386747	1.661876	2.000777
C	-5.614465	-0.703490	1.842848	H	6.291161	1.361569	1.462220
H	-6.439485	-0.398187	1.191441	H	5.113885	0.847055	2.675097
H	-5.309997	0.167307	2.428934	H	5.628205	2.540047	2.607893
H	-5.989191	-1.464581	2.534488	C	-0.114181	1.141503	3.516246
C	-0.177113	-0.402392	3.673777	H	0.058408	0.110627	3.199128
H	-0.246799	0.578484	3.198788	H	-1.109709	1.404608	3.142738
H	0.798464	-0.804125	3.379200	C	-0.109323	1.244372	5.045141
C	-0.225822	-0.268567	5.199360	H	-0.851313	0.569439	5.482296
H	0.563911	0.399326	5.556319	H	-0.344856	2.262620	5.370138
H	-0.090654	-1.241091	5.682755	H	0.871454	0.988513	5.458630
H	-1.188058	0.131438	5.535078	N	2.113344	-1.952721	1.883315
N	-1.940720	2.563204	1.483465	C	3.099947	-1.888357	2.946357
C	-2.959281	2.781452	2.496049	H	4.134977	-1.950938	2.571013
H	-3.962253	2.941754	2.066707	H	2.944943	-2.689362	3.675908
H	-2.708607	3.644961	3.120071	C	2.812792	-0.503753	3.516816
C	-2.895196	1.462767	3.257522	H	1.990446	-0.560347	4.247465
H	-2.110131	1.509115	4.027059	H	3.677883	-0.046349	4.004520
H	-3.839067	1.198762	3.742993	C	1.924340	-3.127230	1.111345
C	-1.576163	3.579342	0.565421	C	0.896296	-4.007842	1.489760
C	-0.398055	4.306681	0.825748	C	0.729365	-5.190795	0.764419
C	-0.026137	5.316535	-0.061817	H	-0.056423	-5.887187	1.039515
H	0.882887	5.882822	0.113639	C	1.560232	-5.487169	-0.307151
C	-0.814967	5.618860	-1.166814	H	1.421766	-6.410636	-0.861319
H	-0.518295	6.414376	-1.843709	C	2.563423	-4.599319	-0.682317
C	-1.982134	4.906319	-1.402813	H	3.194679	-4.842416	-1.530458
H	-2.598517	5.147448	-2.265286	C	2.761042	-3.406728	0.012274
C	-2.375413	3.869665	-0.553291	C	3.838760	-2.416531	-0.377948
C	-3.622071	3.078842	-0.881532	H	4.650933	-2.463823	0.359187
H	-4.462419	3.774971	-0.991891	H	3.406455	-1.412966	-0.296950

H	-3.866163	2.400632	-0.061736
C	-3.461749	2.250523	-2.159264
H	-4.386449	1.712920	-2.392285
H	-2.666225	1.516827	-2.006018
H	-3.207271	2.878068	-3.019796
C	0.407460	4.003490	2.071549
H	0.288472	2.942130	2.302293
H	-0.048853	4.535176	2.917717
C	1.891387	4.359764	1.996931
H	2.405926	4.001230	2.892999
H	2.056656	5.439828	1.937099
H	2.365830	3.897258	1.124474
B	-1.013987	0.533533	0.186139

$TS_{A3\rightarrow 6}$

E(scf) = –2459.04718058 a.u.

ν_{min} = –726.9 cm^{-1}

N	2.287180	-1.548224	0.492566
C	1.651676	-0.383964	0.510771
B	0.319908	-0.234235	-0.323077
N	1.320776	-0.058993	-2.643167
C	2.136734	0.717225	1.279676
H	1.561011	1.634375	1.257152
N	-0.501287	-1.267204	-2.613070
C	3.306968	0.617040	1.967799
H	3.714359	1.462119	2.517631
C	5.245981	-0.821665	2.617231
H	5.684793	0.003490	3.172365
C	4.013770	-0.617107	1.952002
C	5.878852	-2.039354	2.560111
H	6.826218	-2.186019	3.069033
C	3.439429	-1.683795	1.212693
C	4.105250	-2.934268	1.182746
H	3.642952	-3.735205	0.614370
C	5.298224	-3.107262	1.838902

C	4.418178	-2.595264	-1.778535
H	5.124770	-1.789154	-1.997229
H	3.626946	-2.559931	-2.535880
H	4.956616	-3.541070	-1.895021
C	0.012579	-3.669850	2.671741
H	0.003511	-2.582260	2.782163
H	0.482245	-4.053874	3.587738
C	-1.420535	-4.195644	2.584975
H	-2.013317	-3.806014	3.417847
H	-1.467561	-5.287705	2.636714
H	-1.900486	-3.881823	1.651870
B	0.976297	-0.318615	0.283556

6

E(scf) = –2459.08677184 a.u.

N	-2.632391	0.025046	-0.812513
C	-1.751200	0.174227	0.170331
B	-0.293957	-0.408089	-0.038525
N	-1.020933	-2.859718	0.458696
C	-2.136943	0.783728	1.408656
H	-1.384685	0.883803	2.183781
N	0.564828	-2.652510	-1.030413
C	-3.426000	1.168755	1.623596
H	-3.740850	1.583963	2.578384
C	-5.729321	1.421052	0.686323
H	-6.079504	1.839594	1.626778
C	-4.373370	1.029196	0.575772
C	-6.586956	1.281274	-0.377191
H	-7.624586	1.586515	-0.285737
C	-3.908106	0.470582	-0.641633
C	-4.812751	0.342880	-1.725562
H	-4.428275	-0.079048	-2.648783
C	-6.119363	0.741367	-1.597950
H	-6.804257	0.638429	-2.434237

H	5.804673	-4.066799	1.802214	C	1.524136	1.717495	0.265951
C	-1.630397	0.861835	1.429795	H	2.132546	-0.453014	0.121662
H	-1.207904	-0.892152	-0.061625	C	1.350467	-2.128246	-2.103095
C	-1.504377	-2.183954	-2.174271	C	2.745063	-2.237311	-2.011625
C	-2.848619	-1.822365	-2.323726	C	3.514968	-1.840745	-3.105340
C	-3.823669	-2.780106	-2.042010	H	4.597167	-1.911865	-3.044093
H	-4.871918	-2.518492	-2.160223	C	2.918025	-1.344999	-4.254805
C	-3.468919	-4.041232	-1.588426	H	3.527896	-1.039845	-5.099506
H	-4.236278	-4.776801	-1.367548	C	1.534993	-1.229771	-4.315987
C	-2.130495	-4.350405	-1.375668	H	1.068640	-0.821414	-5.208263
H	-1.860571	-5.320292	-0.968571	C	0.720364	-1.609987	-3.249064
C	-1.119294	-3.434460	-1.659528	C	-0.769274	-1.366117	-3.343129
C	0.319146	-3.790712	-1.350512	H	-0.953632	-0.838892	-4.285754
H	0.308741	-4.740723	-0.806322	H	-1.085396	-0.681522	-2.546244
H	0.751294	-3.055098	-0.661233	C	-1.670596	-2.603732	-3.298990
C	1.249772	-3.933781	-2.559895	H	-2.699937	-2.316233	-3.530900
H	2.215517	-4.334661	-2.238357	H	-1.692004	-3.058846	-2.306132
H	1.451413	-2.971011	-3.036089	H	-1.355161	-3.361951	-4.023578
H	0.833806	-4.611835	-3.312295	C	3.435688	-2.718357	-0.755779
C	-3.264609	-0.424908	-2.721275	H	2.707982	-2.812853	0.053435
H	-2.404262	0.247195	-2.657095	H	4.127356	-1.935496	-0.424147
H	-3.970429	-0.063026	-1.964532	C	4.206559	-4.028350	-0.945652
C	-3.925929	-0.338377	-4.100198	H	4.686196	-4.334468	-0.011452
H	-4.203761	0.694590	-4.329227	H	4.987969	-3.923558	-1.704767
H	-4.833253	-0.948699	-4.142826	H	3.547244	-4.839634	-1.271981
H	-3.259074	-0.695042	-4.891725	C	-0.261570	-1.966523	-0.217091
C	0.375111	-0.598732	-1.846227	C	-0.660720	-4.248399	0.176733
C	1.041262	-0.248609	-4.064467	H	-1.534550	-4.820841	-0.148529
H	1.885288	-0.734206	-4.564245	H	-0.261024	-4.723238	1.079558
H	0.869687	0.720414	-4.544648	C	0.397048	-4.103954	-0.925844
C	-0.223042	-1.122817	-4.044907	H	1.342855	-4.587105	-0.664454
H	-1.065138	-0.643753	-4.552669	H	0.062132	-4.507015	-1.886813
H	-0.066582	-2.107467	-4.495177	C	-1.831953	-2.540207	1.592632
C	2.310507	0.866886	-2.184309	C	-1.204232	-2.234459	2.811190
C	1.939556	2.189399	-1.922325	C	-2.022694	-1.919267	3.895459
C	2.913405	3.049367	-1.406992	H	-1.578292	-1.670108	4.852720

H	2.635419	4.073210	-1.169791	C	-3.405897	-1.921639	3.766698
C	4.207341	2.605711	-1.186814	H	-4.026173	-1.668790	4.621324
H	4.950939	3.284032	-0.779567	C	-4.002444	-2.247864	2.556027
C	4.559858	1.291048	-1.481484	H	-5.083162	-2.235682	2.473329
H	5.574697	0.959589	-1.294202	C	-3.225737	-2.569196	1.443788
C	3.619912	0.395487	-1.982782	C	-3.836438	-2.906110	0.099925
C	3.947069	-1.054787	-2.268810	H	-3.594600	-3.947931	-0.150995
H	3.787874	-1.252818	-3.337390	H	-3.340013	-2.288024	-0.655510
H	3.218132	-1.670508	-1.730753	C	-5.346470	-2.713516	-0.003003
C	5.354138	-1.499538	-1.882831	H	-5.677124	-2.913317	-1.025462
H	5.475892	-2.567019	-2.084333	H	-5.633904	-1.685965	0.238673
H	5.537975	-1.345596	-0.815334	H	-5.891792	-3.391191	0.662307
H	6.123746	-0.963481	-2.448013	C	0.307412	-2.222235	2.905078
C	0.549518	2.723013	-2.166435	H	0.684408	-1.474087	2.194957
H	0.222834	3.238166	-1.261144	H	0.696297	-3.187115	2.553323
H	-0.164048	1.909541	-2.307359	C	0.887319	-1.923636	4.282506
C	0.489051	3.692297	-3.349790	H	1.979566	-1.907396	4.224128
H	-0.524025	4.088993	-3.464337	H	0.595785	-2.668847	5.029974
H	0.774811	3.201798	-4.286917	H	0.565195	-0.940891	4.641939
H	1.165608	4.539810	-3.202968	N	0.861521	2.846848	-0.108295
N	-2.356518	0.136325	2.372286	C	-0.314780	2.912829	-0.916177
C	-2.282961	-1.287498	2.386291	C	-1.472730	3.488798	-0.372019
C	-1.167568	-1.957453	2.908709	C	-2.617390	3.573097	-1.165782
C	-1.163442	-3.354472	2.881536	H	-3.526455	3.986132	-0.736989
H	-0.300570	-3.883875	3.277742	C	-2.617346	3.098549	-2.466653
C	-2.225155	-4.063795	2.340247	H	-3.524090	3.139014	-3.061296
H	-2.202032	-5.149459	2.323583	C	-1.457184	2.547614	-2.994769
C	-3.303616	-3.381586	1.788698	H	-1.455644	2.171696	-4.014069
H	-4.116623	-3.932782	1.325321	C	-0.283980	2.455905	-2.247565
C	-3.340105	-1.989751	1.785725	C	0.937683	1.838621	-2.886365
C	-4.472109	-1.254182	1.108094	H	0.645747	1.473110	-3.875890
H	-4.967750	-1.949750	0.422878	H	1.223590	0.955133	-2.305255
H	-4.044871	-0.453970	0.493652	C	2.150364	2.757752	-3.050165
C	-5.508456	-0.671119	2.072205	H	2.925627	2.244410	-3.627641
H	-6.339803	-0.218487	1.522642	H	2.593328	3.022692	-2.087077
H	-5.063614	0.104466	2.700232	H	1.889021	3.683107	-3.573676

H	-5.916615	-1.446454	2.728117
C	0.031649	-1.235389	3.479838
H	0.025836	-0.188159	3.169936
H	0.933782	-1.667914	3.034062
C	0.124175	-1.341237	5.005528
H	1.007817	-0.814447	5.377648
H	0.196120	-2.386082	5.323037
H	-0.759680	-0.913324	5.489837
N	-1.661673	2.182420	1.862489
C	-2.521260	2.335421	3.029105
H	-3.533592	2.661756	2.743371
H	-2.110255	3.065401	3.732724
C	-2.531310	0.916015	3.587645
H	-1.703236	0.775171	4.298148
H	-3.465209	0.659753	4.095292
C	-1.372710	3.310716	1.046565
C	-0.282657	4.122185	1.404782
C	-0.022871	5.265405	0.644056
H	0.815892	5.904652	0.900701
C	-0.829620	5.598761	-0.433753
H	-0.618509	6.491835	-1.013984
C	-1.910592	4.791218	-0.773169
H	-2.532981	5.068601	-1.617201
C	-2.205436	3.640145	-0.044807
C	-3.370312	2.743359	-0.400964
H	-4.038629	2.664886	0.466193
H	-2.965599	1.732422	-0.546248
C	-4.176341	3.143298	-1.631474
H	-4.971104	2.412140	-1.805877
H	-3.546926	3.163776	-2.528132
H	-4.647131	4.126144	-1.524880
C	0.568772	3.776808	2.608396
H	0.454321	2.708575	2.811697
H	0.161877	4.292066	3.489427
C	2.053111	4.121126	2.472243
H	2.607675	3.733547	3.331780

C	-1.532792	4.032743	1.036536
H	-0.718542	3.614612	1.635767
H	-2.458979	3.680394	1.499950
C	-1.513242	5.565832	1.079001
H	-1.539938	5.931111	2.110217
H	-2.380920	5.975163	0.552924
H	-0.621400	5.974902	0.594268
N	2.734422	2.108414	0.753694
C	2.970455	3.542091	0.674221
H	3.788967	3.769216	-0.021222
H	3.244520	3.947575	1.653927
C	1.615911	4.068798	0.181359
H	1.114892	4.667155	0.948498
H	1.701658	4.679661	-0.722204
C	3.671814	1.238844	1.381001
C	3.522170	0.959088	2.745378
C	4.473558	0.138542	3.352167
H	4.392992	-0.095465	4.408350
C	5.529842	-0.382732	2.615573
H	6.265709	-1.015294	3.102672
C	5.646869	-0.109548	1.257352
H	6.468940	-0.541073	0.696350
C	4.713043	0.701143	0.612922
C	4.730774	0.946954	-0.880078
H	4.684323	2.024855	-1.074157
H	3.797692	0.535653	-1.288167
C	5.918824	0.361556	-1.634523
H	5.821102	0.570990	-2.703016
H	5.974528	-0.725709	-1.517487
H	6.868401	0.784697	-1.291130
C	2.319491	1.499897	3.488684
H	1.435685	0.963455	3.115634
H	2.160454	2.544905	3.198665
C	2.384357	1.412164	5.010104
H	1.493581	1.867935	5.450986
H	3.261460	1.935847	5.403583

H	2.226618	5.200519	2.434988
H	2.479222	3.681301	1.564404
B	-1.051991	0.322293	0.199813

H	2.426078	0.376253	5.358206
B	1.142141	0.237757	0.100005

$TS_{6\rightarrow A4}$

E(scf) = –2459.04128728 a.u.

ν_{min} = –244.9 cm^{-1}

N	-0.954257	1.596677	-0.506856
C	-1.562805	0.548828	0.105786
B	-0.562541	-0.614311	-0.014714
N	-0.072273	-3.002614	-0.844062
C	-2.861610	0.754406	0.678334
H	-3.308254	-0.046795	1.255361
N	-2.205703	-2.333389	-1.046174
C	-3.517471	1.926337	0.479576
H	-4.514985	2.082819	0.882794
C	-3.549642	4.203755	-0.549956
H	-4.569635	4.347436	-0.201538
C	-2.907305	2.981531	-0.270544
C	-2.901950	5.206733	-1.238659
H	-3.405862	6.145499	-1.443214
C	-1.575836	2.782208	-0.723505
C	-0.928490	3.818312	-1.436131
H	0.078634	3.637939	-1.796974
C	-1.579530	5.006052	-1.681203
H	-1.073329	5.793424	-2.232193
C	1.643614	1.361811	0.467436
H	1.236727	0.397716	-1.585783
C	-3.242453	-1.446479	-1.439388
C	-3.045556	-0.493988	-2.460353
C	-4.096569	0.362897	-2.779642
H	-3.940492	1.117548	-3.546269
C	-5.331994	0.264066	-2.148493
H	-6.136887	0.940607	-2.419257
C	-5.523673	-0.697679	-1.168707

A4

E(scf) = –2459.06427238 a.u.

N	-0.720971	-1.580751	0.431586
C	-1.531511	-0.630477	-0.126234
B	-0.609546	0.572548	0.011285
N	-0.351416	3.067896	0.702444
C	-2.810098	-1.059543	-0.616603
H	-3.419158	-0.363081	-1.179713
N	-2.376957	2.153627	0.999446
C	-3.244413	-2.315404	-0.347288
H	-4.222387	-2.647940	-0.685976
C	-2.875682	-4.516081	0.786792
H	-3.877067	-4.829193	0.501527
C	-2.438701	-3.235189	0.412262
C	-2.057621	-5.369484	1.503776
H	-2.409964	-6.355902	1.785993
C	-1.132686	-2.825464	0.783332
C	-0.309221	-3.694491	1.526192
H	0.668783	-3.341726	1.833662
C	-0.770634	-4.947334	1.875883
H	-0.134714	-5.608796	2.456884
C	1.696062	-1.335532	-0.412551
H	1.039550	-0.659769	1.673580
C	-3.299777	1.176689	1.461079
C	-2.972013	0.305535	2.521079
C	-3.914952	-0.638960	2.918531
H	-3.655306	-1.330957	3.715410
C	-5.173621	-0.703160	2.329683
H	-5.894659	-1.443316	2.662594
C	-5.496070	0.180893	1.312970
H	-6.473168	0.134029	0.837854

H	-6.483080	-0.778812	-0.662995	C	-4.563771	1.114087	0.853942
C	-4.483628	-1.548236	-0.790608	C	-4.929413	2.018154	-0.299980
C	-4.703531	-2.543879	0.323515	H	-5.446044	1.424852	-1.064259
H	-5.253410	-2.052181	1.135405	H	-4.014259	2.401686	-0.760432
H	-3.733129	-2.841360	0.731224	C	-5.836520	3.182159	0.116151
C	-5.487684	-3.781759	-0.126453	H	-6.082122	3.817400	-0.740396
H	-5.610720	-4.493019	0.696042	H	-5.354866	3.804980	0.875415
H	-4.974983	-4.294384	-0.945840	H	-6.772912	2.810220	0.543714
H	-6.482864	-3.503253	-0.487404	C	-1.662305	0.392129	3.266329
C	-1.756608	-0.402341	-3.241156	H	-0.857501	0.665669	2.581363
H	-0.911864	-0.645288	-2.593343	H	-1.414580	-0.602545	3.652327
H	-1.614951	0.636153	-3.558801	C	-1.727239	1.383911	4.432994
C	-1.759266	-1.314325	-4.472689	H	-0.766278	1.432657	4.954142
H	-0.812272	-1.234222	-5.015357	H	-2.496306	1.095819	5.156715
H	-2.571028	-1.053362	-5.159333	H	-1.968239	2.392515	4.081844
H	-1.894363	-2.362960	-4.188356	C	-1.088201	1.885639	0.488997
C	-0.943064	-1.929492	-0.552391	C	-1.057225	3.956918	1.623466
C	-0.686173	-3.924410	-1.795894	H	-0.885079	5.003662	1.362998
H	-0.385986	-4.954732	-1.590210	H	-0.723475	3.792509	2.657268
H	-0.398143	-3.676716	-2.827835	C	-2.515132	3.530472	1.455972
C	-2.182808	-3.685017	-1.587324	H	-3.077452	3.567150	2.393406
H	-2.752227	-3.734745	-2.520230	H	-3.039920	4.162511	0.723553
H	-2.615043	-4.420975	-0.891606	C	0.460615	3.634193	-0.315350
C	0.839999	-3.510315	0.117170	C	1.750269	4.069854	0.046093
C	2.180679	-3.683183	-0.278656	C	2.586128	4.589346	-0.940598
C	3.098564	-4.174801	0.648666	H	3.587169	4.920425	-0.683247
H	4.135152	-4.319588	0.360956	C	2.159803	4.678997	-2.261946
C	2.708098	-4.481902	1.947700	H	2.828592	5.067812	-3.023878
H	3.437035	-4.850268	2.663306	C	0.858456	4.324884	-2.588200
C	1.373382	-4.362426	2.308410	H	0.496590	4.468548	-3.603737
H	1.052859	-4.659429	3.304499	C	-0.024479	3.836983	-1.622328
C	0.414517	-3.911908	1.399256	C	-1.485322	3.682402	-1.996660
C	-1.043083	-3.969103	1.807045	H	-1.783347	4.609727	-2.501094
H	-1.214249	-4.949190	2.268666	H	-2.090844	3.616552	-1.093522
H	-1.677052	-3.928790	0.922094	C	-1.829454	2.496730	-2.897144
C	-1.493243	-2.875031	2.775196	H	-2.887708	2.526198	-3.176396

H	-2.541999	-3.018686	3.054644
H	-1.390327	-1.895162	2.302056
H	-0.895352	-2.893461	3.692669
C	2.585009	-3.305691	-1.690137
H	2.546987	-2.212209	-1.783620
H	1.825969	-3.667642	-2.388477
C	3.938491	-3.833416	-2.159593
H	4.115503	-3.538599	-3.197520
H	3.977578	-4.926181	-2.105335
H	4.774404	-3.448628	-1.566737
N	2.901200	1.689134	0.121149
C	3.627416	1.264205	-1.034160
C	3.707392	2.107722	-2.146181
C	4.492988	1.697387	-3.224347
H	4.555806	2.332100	-4.104097
C	5.173684	0.489567	-3.189428
H	5.778575	0.181997	-4.036465
C	5.065119	-0.338600	-2.077597
H	5.579404	-1.294525	-2.063776
C	4.287164	0.028024	-0.980608
C	4.129610	-0.917834	0.188136
H	4.482552	-1.899444	-0.131812
H	3.066442	-1.055548	0.415791
C	4.884785	-0.541876	1.464774
H	4.796962	-1.352124	2.194304
H	4.481837	0.358703	1.934154
H	5.947028	-0.373234	1.263470
C	2.963179	3.420001	-2.218750
H	2.231415	3.479508	-1.408967
H	2.385826	3.438232	-3.149891
C	3.890626	4.639480	-2.176159
H	3.316457	5.568163	-2.235752
H	4.598937	4.625178	-3.009571
H	4.477369	4.659999	-1.252115
N	1.382840	1.930692	1.657877
C	2.580217	2.545454	2.244056
H	-1.635996	1.557251	-2.373861
H	-1.238975	2.518519	-3.819401
C	2.187818	3.940569	1.491643
H	2.184694	2.876110	1.758937
H	1.427412	4.392959	2.136069
C	3.528451	4.579470	1.844546
H	3.741603	4.434186	2.907193
H	3.520873	5.656112	1.646848
H	4.364126	4.150088	1.281892
N	3.000363	-1.409525	-0.084404
C	3.628916	-0.847196	1.067416
C	3.897700	-1.653212	2.178378
C	4.569815	-1.079650	3.258890
H	4.773649	-1.686655	4.137019
C	4.961854	0.250388	3.228667
H	5.477347	0.684511	4.079453
C	4.678378	1.032530	2.114786
H	4.971308	2.077766	2.099874
C	4.007704	0.501399	1.014379
C	3.667765	1.382927	-0.164179
H	3.836855	2.415324	0.138383
H	2.595536	1.311200	-0.380719
C	4.477070	1.151223	-1.442172
H	4.219001	1.921466	-2.175083
H	4.269168	0.183271	-1.903743
H	5.552456	1.209128	-1.246766
C	3.483008	-3.104192	2.246650
H	2.780404	-3.332652	1.440969
H	2.937017	-3.265970	3.182822
C	4.674223	-4.067427	2.185490
H	4.339203	-5.106649	2.247348
H	5.370076	-3.885818	3.009713
H	5.234869	-3.943654	1.253619
N	1.539228	-1.970482	-1.594356
C	2.822811	-2.357850	-2.188539
H	3.082053	-1.652500	-2.989290

H	2.981147	1.878299	3.018743
H	2.346096	3.506037	2.702908
C	3.499190	2.663285	1.037715
H	3.459715	3.662126	0.587454
H	4.541097	2.415883	1.246536
C	0.284117	1.664348	2.535531
C	-0.614079	2.711221	2.797972
C	-1.657814	2.457919	3.683766
H	-2.389552	3.230506	3.890236
C	-1.773362	1.221631	4.307715
H	-2.594729	1.038563	4.993550
C	-0.832601	0.228337	4.081625
H	-0.922676	-0.718811	4.600804
C	0.226659	0.428913	3.193747
C	1.262452	-0.648793	2.961550
H	2.140920	-0.215396	2.475540
H	0.860915	-1.362056	2.232652
C	1.725930	-1.380132	4.220262
H	2.503820	-2.101845	3.957715
H	0.915949	-1.941888	4.693575
H	2.128202	-0.683623	4.963353
C	-0.409706	4.073024	2.166926
H	-0.190198	3.941385	1.104942
H	0.489303	4.527968	2.605816
C	-1.562991	5.058533	2.323961
H	-1.350502	5.970818	1.761298
H	-1.720582	5.337601	3.370642
H	-2.491415	4.639771	1.929703
B	0.723090	0.426995	-0.494975

$TS_{A4 \rightarrow A5}$

E(scf) = –2459.05148000 a.u.

ν_{min} = –115.2 cm^{-1}

N	-1.563659	-0.928161	-0.835159
C	-1.632494	0.139327	-0.003678

H	2.768771	-3.360407	-2.615903
C	3.766984	-2.259820	-0.997815
H	3.943817	-3.237436	-0.534245
H	4.731906	-1.808610	-1.235440
C	0.409071	-1.878851	-2.467940
C	-0.354212	-3.036819	-2.687561
C	-1.438481	-2.941803	-3.555602
H	-2.067185	-3.807134	-3.730799
C	-1.724835	-1.746057	-4.203948
H	-2.576428	-1.687193	-4.874747
C	-0.916140	-0.635753	-4.018861
H	-1.135681	0.280928	-4.554510
C	0.178691	-0.677771	-3.151758
C	1.070157	0.532282	-2.978014
H	1.968909	0.256540	-2.423409
H	0.557716	1.252492	-2.331865
C	1.498823	1.202588	-4.282807
H	2.139712	2.060192	-4.059373
H	0.645579	1.578060	-4.855112
H	2.052268	0.511249	-4.927013
C	0.034998	-4.343881	-2.028335
H	0.250335	-4.159989	-0.973568
H	0.980830	-4.686381	-2.471370
C	-0.985993	-5.471809	-2.139748
H	-0.646748	-6.337213	-1.565116
H	-1.130631	-5.793044	-3.176204
H	-1.951600	-5.164987	-1.730371
B	0.573871	-0.694337	0.553094

A5

E(scf) = –2459.05928915 a.u.

N	-2.171147	0.081947	0.652867
C	-1.576145	0.229034	-0.539723
B	-0.040405	-0.019336	-0.122107

B	-0.127727	0.525093	0.053626	N	1.853705	-1.692632	-0.877725
N	1.436143	2.670753	-0.186248	C	-2.356827	0.509651	-1.671825
C	-2.917395	0.476217	0.524944	H	-1.874509	0.688131	-2.624412
H	-2.994564	1.265627	1.259362	N	-0.233959	-2.451700	-0.938263
N	-0.769060	2.951822	-0.228268	C	-3.722858	0.556202	-1.545438
C	-4.017948	-0.200875	0.109006	H	-4.349346	0.781033	-2.403886
H	-5.000037	0.057222	0.494730	C	-5.748159	0.194263	-0.133290
C	-5.032335	-1.985241	-1.322831	H	-6.389021	0.366954	-0.993109
H	-6.022342	-1.711393	-0.968390	C	-4.343192	0.276731	-0.302150
C	-3.918676	-1.271478	-0.839670	C	-6.287749	-0.104174	1.091778
C	-4.874483	-3.010912	-2.231023	H	-7.364047	-0.171101	1.210725
H	-5.740026	-3.550851	-2.599934	C	-3.522266	0.045989	0.827120
C	-2.633615	-1.636025	-1.298119	C	-4.085029	-0.247973	2.086041
C	-2.476084	-2.681855	-2.227656	H	-3.420973	-0.421970	2.925665
H	-1.477333	-2.931106	-2.571541	C	-5.448495	-0.322541	2.210524
C	-3.587241	-3.355686	-2.684747	H	-5.892166	-0.549865	3.174203
H	-3.469266	-4.158720	-3.405396	C	0.193661	1.053815	1.061611
C	0.592349	-1.431070	0.409885	H	-0.928646	-0.687958	2.523844
H	0.533165	-1.172185	-1.949455	C	-1.531813	-2.779098	-0.453835
C	-1.977484	2.845920	-0.971924	C	-2.621223	-2.739411	-1.335483
C	-3.157607	3.349440	-0.391772	C	-3.891899	-3.051070	-0.847311
C	-4.360456	3.255827	-1.092874	H	-4.742387	-2.999989	-1.522025
H	-5.266920	3.638013	-0.629227	C	-4.076645	-3.410470	0.479110
C	-4.421180	2.641400	-2.332191	H	-5.073258	-3.619087	0.854319
H	-5.368256	2.539558	-2.852251	C	-2.978043	-3.504689	1.325106
C	-3.249469	2.161022	-2.904047	H	-3.113713	-3.817604	2.357083
H	-3.279995	1.698130	-3.886878	C	-1.691759	-3.213654	0.873263
C	-2.016138	2.284748	-2.268019	C	-0.513117	-3.459269	1.782537
C	-0.778477	1.895328	-3.040621	H	-0.770805	-3.137588	2.796694
H	-1.046688	1.115933	-3.761374	H	0.333174	-2.844620	1.475268
H	-0.027148	1.458115	-2.382795	C	-0.099786	-4.934956	1.808731
C	-0.192911	3.095875	-3.791774	H	0.738307	-5.087356	2.495220
H	0.706053	2.809664	-4.346822	H	0.210253	-5.279077	0.816615
H	0.079232	3.897228	-3.096823	H	-0.927304	-5.573324	2.132949
H	-0.916643	3.509171	-4.501384	C	-2.440811	-2.445108	-2.804999
C	-3.195003	3.940622	1.000910	H	-1.509225	-1.893527	-2.958439

H	-2.301925	3.644141	1.555616	H	-3.251898	-1.791392	-3.142270
H	-4.048343	3.505172	1.535339	C	-2.438362	-3.724388	-3.650188
C	-3.341492	5.466384	1.016850	H	-2.288669	-3.494911	-4.709293
H	-3.408134	5.842617	2.042127	H	-3.387775	-4.258474	-3.547460
H	-4.245620	5.775707	0.483732	H	-1.643489	-4.406500	-3.334054
H	-2.491498	5.954657	0.532252	C	0.535962	-1.335050	-0.603696
C	0.238845	1.957920	-0.094634	C	1.944783	-3.142808	-1.125619
C	1.235586	4.107030	-0.366910	H	2.170396	-3.677652	-0.195491
H	1.444240	4.417715	-1.400365	H	2.718284	-3.356890	-1.862876
H	1.904895	4.668110	0.291786	C	0.549862	-3.473893	-1.616227
C	-0.224114	4.284231	0.001068	H	0.466317	-3.384750	-2.709906
H	-0.332157	4.588601	1.052023	H	0.214732	-4.471498	-1.325109
H	-0.733117	5.017883	-0.626561	C	2.793219	-0.857576	-1.560540
C	2.798650	2.333912	0.059185	C	2.409652	-0.067981	-2.666342
C	3.272257	2.281110	1.380701	C	3.385772	0.719137	-3.282363
C	4.650044	2.278277	1.598086	H	3.108542	1.347154	-4.121382
H	5.035631	2.235729	2.611479	C	4.709311	0.683270	-2.873568
C	5.537565	2.372509	0.532325	H	5.449762	1.302816	-3.369411
H	6.606685	2.406446	0.718182	C	5.089278	-0.180656	-1.855614
C	5.056106	2.402801	-0.769752	H	6.133874	-0.240078	-1.573166
H	5.751002	2.446113	-1.604324	C	4.151368	-0.967390	-1.191005
C	3.686294	2.357741	-1.029282	C	4.577550	-1.936306	-0.108470
C	3.180302	2.289429	-2.449113	H	4.235647	-2.941547	-0.370645
H	3.944026	2.688438	-3.125585	H	4.049815	-1.679663	0.814976
H	2.292004	2.913582	-2.567884	C	6.078173	-2.037504	0.145410
C	2.826095	0.852561	-2.848158	H	6.264850	-2.740167	0.962035
H	2.391868	0.823496	-3.852516	H	6.512473	-1.078900	0.436077
H	2.096400	0.428923	-2.153364	H	6.609222	-2.402228	-0.740318
H	3.708155	0.205508	-2.838018	C	1.010270	-0.099799	-3.249528
C	2.273312	2.293576	2.511531	H	0.385215	0.642679	-2.742233
H	1.422137	1.684574	2.201800	H	0.552324	-1.063855	-3.019969
H	1.873435	3.312187	2.619552	C	0.927557	0.110517	-4.762068
C	2.791915	1.804109	3.859534	H	-0.098360	-0.062665	-5.101450
H	1.971803	1.744514	4.580766	H	1.584469	-0.580440	-5.298944
H	3.550464	2.471416	4.279860	H	1.196295	1.127688	-5.059397
H	3.234961	0.805977	3.772613	N	1.299122	1.114284	2.025577

N	1.948665	-1.781681	0.454586	C	2.605078	0.539843	2.047128
C	2.723333	-2.338180	-0.613931	C	3.682252	1.165207	1.383897
C	4.024932	-1.850761	-0.819381	C	4.987656	0.817754	1.731625
C	4.810383	-2.385141	-1.843494	H	5.819740	1.314096	1.240440
H	5.807454	-1.979140	-1.995599	C	5.240340	-0.145572	2.698815
C	4.334404	-3.388225	-2.668824	H	6.262851	-0.391332	2.969461
H	4.945262	-3.778236	-3.476725	C	4.179142	-0.819756	3.286296
C	3.066622	-3.900397	-2.431267	H	4.369518	-1.599545	4.019467
H	2.690381	-4.717956	-3.040845	C	2.858338	-0.492633	2.971756
C	2.263318	-3.418845	-1.399122	C	1.737046	-1.236390	3.656191
C	0.991564	-4.170750	-1.105753	H	2.129331	-2.199551	4.004773
H	0.626655	-4.630637	-2.030806	H	0.959065	-1.450822	2.924305
H	0.216935	-3.488053	-0.770750	C	1.098119	-0.496790	4.833899
C	1.213283	-5.261916	-0.053921	H	0.393394	-1.149707	5.358875
H	0.289334	-5.816167	0.136702	H	0.545932	0.374330	4.476284
H	1.549437	-4.825282	0.892006	H	1.853707	-0.163547	5.552616
H	1.978326	-5.971662	-0.382681	C	3.419834	2.205127	0.326145
C	4.635300	-0.768117	0.026497	H	2.379711	2.511661	0.408918
H	3.855509	-0.178805	0.509436	H	3.512253	1.730806	-0.656947
H	5.160832	-0.081993	-0.638626	C	4.320989	3.438059	0.370864
C	5.644577	-1.279063	1.059922	H	3.993700	4.167608	-0.376603
H	6.079386	-0.435938	1.604989	H	5.366204	3.197793	0.153124
H	6.458488	-1.826287	0.573563	H	4.286816	3.920432	1.353260
H	5.188350	-1.952971	1.790032	N	-0.182570	2.484768	0.944425
N	0.080401	-1.802794	1.669342	C	-0.049906	3.013289	2.288333
C	1.102991	-2.357487	2.544024	H	-0.841617	2.682345	2.975085
H	0.988498	-3.447969	2.634292	H	-0.024424	4.106366	2.278571
H	1.040097	-1.929999	3.549779	C	1.265323	2.394080	2.730269
C	2.388411	-1.968579	1.832293	H	2.111149	3.035511	2.443660
H	2.805360	-1.040321	2.248260	H	1.314347	2.259314	3.818479
H	3.150768	-2.746731	1.884321	C	-1.115055	3.028880	0.020806
C	-1.237260	-1.697548	2.208820	C	-0.634071	3.313668	-1.280617
C	-1.527323	-0.688082	3.143299	C	-1.510866	3.791595	-2.250162
C	-2.799918	-0.651286	3.717251	H	-1.146168	3.992191	-3.252122
H	-3.029819	0.118967	4.447872	C	-2.839235	4.052282	-1.943704
C	-3.768711	-1.577208	3.371077	H	-3.515646	4.429444	-2.704785

H	-4.756785	-1.525798	3.818164
C	-3.463118	-2.591178	2.471505
H	-4.217400	-3.329553	2.224445
C	-2.195966	-2.683825	1.902844
C	-1.813662	-3.845008	1.018128
H	-0.989375	-4.382664	1.502295
H	-1.398464	-3.447743	0.093989
C	-2.926493	-4.822211	0.662022
H	-2.541442	-5.589379	-0.015736
H	-3.748938	-4.314723	0.148274
H	-3.328773	-5.328383	1.545347
C	-0.506132	0.336894	3.577872
H	0.411401	0.190287	3.006336
H	-0.263313	0.155569	4.633784
C	-0.966603	1.786521	3.404795
H	-0.242906	2.477875	3.846820
H	-1.933418	1.970958	3.882305
H	-1.050184	2.020052	2.340421
B	-0.035699	-1.010193	-0.911840

A6

E(scf) = –2459.12954022 a.u.

N	-2.086611	0.580125	-0.106605
C	-0.990324	1.203870	0.394904
B	0.346765	0.517327	0.033697
N	2.919178	1.017885	0.514778
C	-1.213670	2.319749	1.264674
H	-0.354828	2.761945	1.749589
N	1.656466	2.679926	-0.211568
C	-2.460149	2.787972	1.516429
H	-2.617516	3.616014	2.203384
C	-4.899953	2.691390	1.027431
H	-5.064153	3.533745	1.694087
C	-3.588153	2.201616	0.868904
C	-5.948984	2.121581	0.344754

C	-3.289226	3.853784	-0.648341
H	-4.322933	4.080907	-0.413294
C	-2.453564	3.346500	0.350695
C	-3.003940	3.156659	1.753726
H	-2.550791	3.904506	2.415134
H	-2.672509	2.188569	2.136835
C	-4.520021	3.252506	1.906371
H	-4.801399	3.001076	2.932614
H	-5.042913	2.561127	1.239969
H	-4.887490	4.263418	1.704271
C	0.838729	3.156864	-1.569077
H	1.145557	2.127210	-1.363915
H	1.372641	3.760871	-0.827600
C	1.303448	3.560867	-2.962278
H	2.385160	3.424100	-3.040819
H	1.079823	4.610404	-3.179430
H	0.835327	2.949754	-3.741894
B	-0.917727	-0.054622	1.515134

$TS_{A6 \rightarrow A7}$

E(scf) = –2459.09103496 a.u.

ν_{min} = –902.9 cm^{-1}

N	-1.870309	0.928373	0.652145
C	-0.617795	1.527066	0.467938
B	0.455862	0.508405	0.167935
N	2.490754	2.059312	0.655620
C	-0.559017	2.939632	0.708598
H	0.362106	3.438101	0.473039
N	2.517972	0.112402	1.706351
C	-1.631436	3.646231	1.142220
H	-1.569817	4.730365	1.269221
C	-3.944215	3.641749	2.068482
H	-3.873955	4.718907	2.214948
C	-2.854387	2.979224	1.486354

H	-6.957218	2.501984	0.469551	C	-5.072545	2.952119	2.475397
C	-3.354682	1.090979	0.024999	H	-5.904341	3.474496	2.923375
C	-4.433161	0.547025	-0.702384	C	-2.930255	1.585837	1.270664
H	-4.240048	-0.255357	-1.401946	C	-4.051175	0.879339	1.740679
C	-5.701221	1.053298	-0.536678	H	-4.073629	-0.196392	1.657673
H	-6.524122	0.622192	-1.098070	C	-5.105666	1.560350	2.317541
C	-0.067356	-0.752228	-0.679254	H	-5.971988	0.998285	2.661951
H	-1.826752	-0.365894	-2.133458	C	-0.345927	-0.823471	-0.133787
C	0.696908	3.366850	-1.021189	H	-0.849652	-1.279131	1.311636
C	0.065778	4.511086	-0.511254	C	2.107144	-1.059247	2.409773
C	-0.878545	5.163301	-1.306633	C	2.902976	-2.210284	2.291706
H	-1.384881	6.037453	-0.905841	C	2.613369	-3.309855	3.095423
C	-1.190197	4.701853	-2.574194	H	3.229577	-4.203111	3.012287
H	-1.944709	5.204831	-3.170094	C	1.530997	-3.297617	3.961042
C	-0.516746	3.598262	-3.083799	H	1.302531	-4.173203	4.569827
H	-0.730278	3.250002	-4.090215	C	0.743993	-2.162056	4.059668
C	0.449565	2.930092	-2.336678	H	-0.094930	-2.134416	4.742663
C	1.257370	1.839611	-2.995612	C	1.030730	-1.013729	3.311007
H	0.672280	1.422906	-3.821258	C	0.231965	0.240933	3.578657
H	1.431368	1.015891	-2.302789	H	-0.764745	-0.052576	3.936472
C	2.592232	2.365006	-3.534907	H	0.081741	0.798853	2.651886
H	3.161195	1.562579	-4.014142	C	0.904752	1.147734	4.630725
H	3.210015	2.776044	-2.729968	H	0.292739	2.029768	4.813229
H	2.436792	3.160213	-4.270144	H	1.899273	1.496971	4.296075
C	0.377770	5.105303	0.844607	H	1.050157	0.609139	5.595402
H	0.881599	4.375054	1.481918	C	4.069020	-2.305404	1.331897
H	-0.566701	5.345293	1.344979	H	3.996845	-1.509963	0.578561
C	1.232498	6.374086	0.747724	H	3.975482	-3.240111	0.768169
H	1.423055	6.794570	1.739391	C	5.431015	-2.284467	2.023089
H	0.729720	7.137218	0.146748	H	6.243369	-2.346461	1.291926
H	2.196652	6.168102	0.273003	H	5.535527	-3.130010	2.714274
C	1.638496	1.350589	0.139771	H	5.576623	-1.371593	2.613759
C	3.851041	2.110990	0.178668	C	1.829068	0.849570	0.776605
H	4.321977	1.923435	-0.791829	C	3.468085	2.231532	1.733580
H	4.622827	2.202059	0.943274	H	3.044955	2.821641	2.560060
C	2.934507	3.315324	0.107807	H	4.361425	2.740462	1.364379

H	2.884963	3.853648	1.062759	C	3.736099	0.788475	2.155208
H	3.214258	4.021417	-0.676019	H	4.622025	0.378334	1.659611
C	3.296492	0.104772	1.552843	H	3.852406	0.666374	3.233252
C	2.627781	0.105818	2.796692	C	2.707194	2.743805	-0.585804
C	3.088496	-0.758907	3.790632	C	3.296444	2.040463	-1.661010
H	2.581828	-0.786119	4.748190	C	3.446329	2.702031	-2.877848
C	4.202432	-1.559692	3.593759	H	3.876421	2.175694	-3.718895
H	4.546596	-2.218594	4.384507	C	3.093735	4.036599	-3.012290
C	4.889187	-1.498036	2.390054	H	3.216938	4.539943	-3.966866
H	5.775445	-2.106586	2.254914	C	2.654742	4.750345	-1.907108
C	4.457516	-0.671514	1.354206	H	2.462546	5.811709	-1.998455
C	5.226709	-0.605507	0.049913	C	2.476739	4.125459	-0.669313
H	5.545090	0.424323	-0.130553	C	2.153445	4.961329	0.556598
H	4.541488	-0.855663	-0.767622	H	3.083583	5.419142	0.910359
C	6.484901	-1.465034	-0.029335	H	1.814232	4.317384	1.366630
H	6.929473	-1.373997	-1.024022	C	1.111193	6.068278	0.328104
H	6.276009	-2.523034	0.140111	H	0.854639	6.542870	1.279140
H	7.233354	-1.143044	0.701965	H	0.197048	5.664168	-0.121182
C	1.468756	1.032119	3.087979	H	1.487585	6.855226	-0.336374
H	0.573952	0.637305	2.596838	C	3.861448	0.649802	-1.443846
H	1.661424	1.994948	2.599049	H	3.055147	-0.094425	-1.391340
C	1.165405	1.291231	4.561374	H	4.323635	0.633699	-0.454359
H	0.376211	2.043926	4.641443	C	4.913434	0.199184	-2.449052
H	2.044133	1.655992	5.102452	H	5.305104	-0.783085	-2.156100
H	0.801084	0.393721	5.069853	H	5.745557	0.908115	-2.497446
N	0.487148	-1.865575	-1.188801	H	4.504059	0.086541	-3.454844
C	1.862700	-2.259816	-1.218195	N	-2.924839	-1.268780	-0.290088
C	2.417424	-2.918143	-0.110983	C	-4.099053	-0.741544	-0.887236
C	3.661122	-3.532406	-0.265782	C	-4.039026	0.271256	-1.864929
H	4.096531	-4.051869	0.583882	C	-5.232798	0.795893	-2.359849
C	4.338887	-3.491753	-1.476882	H	-5.186142	1.600119	-3.096554
H	5.298608	-3.987954	-1.582061	C	-6.467402	0.319257	-1.941397
C	3.797502	-2.793475	-2.547773	H	-7.380623	0.744742	-2.345453
H	4.333738	-2.743925	-3.491983	C	-6.515864	-0.701152	-1.002530
C	2.550101	-2.176979	-2.440519	H	-7.478422	-1.073958	-0.660783
C	1.941014	-1.516838	-3.657222	C	-5.352412	-1.234085	-0.460916

H	2.651747	-0.780186	-4.051282
H	1.044153	-0.965443	-3.368816
C	1.591497	-2.516797	-4.765100
H	1.156777	-2.003147	-5.627294
H	0.866554	-3.254900	-4.410761
H	2.478180	-3.059758	-5.105978
C	1.684629	-3.044417	1.202703
H	0.843681	-2.348702	1.230110
H	2.365129	-2.745405	2.005565
C	1.194719	-4.471902	1.466600
H	0.646088	-4.521895	2.411614
H	2.031562	-5.175365	1.522945
H	0.527388	-4.816267	0.671264
N	-2.347607	-1.993678	-0.560051
C	-1.581428	-3.181148	-0.849421
H	-2.196827	-3.954108	-1.340194
H	-1.177682	-3.644318	0.069465
C	-0.422604	-2.858622	-1.781848
H	0.149125	-3.766126	-1.986115
H	-0.815636	-2.474590	-2.732831
C	-3.448376	-2.225466	0.297958
C	-3.340695	-2.063726	1.693282
C	-4.458375	-2.312981	2.493537
H	-4.381834	-2.187676	3.570154
C	-5.656882	-2.739292	1.942447
H	-6.513588	-2.932325	2.581349
C	-5.751155	-2.935649	0.570253
H	-6.687295	-3.285866	0.144950
C	-4.662495	-2.688281	-0.261996
C	-4.763685	-2.916785	-1.756293
H	-4.613094	-3.985153	-1.964042
H	-3.924382	-2.397660	-2.229640
C	-6.077090	-2.469489	-2.399906
H	-6.038151	-2.614960	-3.483514
H	-6.266352	-1.408797	-2.209660
H	-6.937977	-3.030918	-2.025468

C	-5.468026	-2.300465	0.605885
H	-6.180876	-1.958029	1.366131
H	-4.506103	-2.428063	1.102729
C	-5.956032	-3.654669	0.061986
H	-6.043056	-4.387403	0.862814
H	-5.259109	-4.050618	-0.689488
H	-6.931589	-3.554337	-0.423383
C	-2.740092	0.815903	-2.427023
H	-1.875413	0.391702	-1.923742
H	-2.690944	1.893758	-2.238665
C	-2.594240	0.552210	-3.929181
H	-1.652348	0.967638	-4.307171
H	-3.408585	0.995634	-4.509588
H	-2.589314	-0.522608	-4.140773
N	-0.130272	-1.909529	-0.959413
C	-1.280985	-2.517964	-1.624530
H	-0.998052	-3.523990	-1.959060
H	-1.568561	-1.957188	-2.530181
C	-2.495124	-2.603419	-0.706232
H	-3.308730	-3.094179	-1.239952
H	-2.242401	-3.207781	0.172175
C	1.126713	-2.363404	-1.450453
C	1.670934	-1.756761	-2.597382
C	2.810391	-2.320095	-3.169212
H	3.243367	-1.883269	-4.058864
C	3.406117	-3.449865	-2.607478
H	4.292507	-3.880159	-3.063710
C	2.850681	-4.047432	-1.486515
H	3.300657	-4.939708	-1.063916
C	1.694107	-3.520746	-0.896964
C	1.069647	-4.227649	0.283209
H	0.457666	-3.527216	0.857769
H	1.866555	-4.566393	0.951131
C	0.234985	-5.447601	-0.117018
H	-0.129095	-5.970080	0.776493
H	0.825751	-6.157886	-0.704069

C	-2.028978	-1.684306	2.347835
H	-1.301036	-1.421847	1.578269
H	-1.628437	-2.569285	2.861517
C	-2.136811	-0.532635	3.348680
H	-1.150590	-0.278023	3.748398
H	-2.777942	-0.784354	4.199199
H	-2.550264	0.358943	2.871709
B	-1.670784	-0.669151	-0.941805

A7

E(scf) = –2459.13749895 a.u.

N	1.747800	0.964362	-0.564108
C	0.441014	1.454864	-0.303931
B	-0.549032	0.331896	-0.252511
N	-2.638445	1.771178	-0.957562
C	0.346134	2.879536	-0.130452
H	-0.579551	3.274088	0.261754
N	-2.425867	-0.195097	-1.931431
C	1.398699	3.702307	-0.325508
H	1.317435	4.766269	-0.120171
C	3.706513	4.024171	-1.229882
H	3.598835	5.097219	-1.095816
C	2.650035	3.192195	-0.844637
C	4.864754	3.499221	-1.782144
H	5.679049	4.154870	-2.071893
C	2.780057	1.796627	-0.994406
C	3.922808	1.280541	-1.613710
H	3.988636	0.216904	-1.805541
C	4.959397	2.122349	-1.986131
H	5.846586	1.698331	-2.445526
C	0.294581	-1.056967	-0.109196
H	0.065525	-1.805436	-0.883726
C	-1.877972	-1.386902	-2.485205

H	-0.642852	-5.173926	-0.714883
C	0.990158	-0.538845	-3.184253
H	1.030912	0.274174	-2.440497
H	-0.078060	-0.754430	-3.303120
C	1.530582	-0.034927	-4.515118
H	0.957109	0.833056	-4.845237
H	1.463904	-0.803265	-5.297667
H	2.579001	0.282225	-4.439820
B	-1.809679	-0.470775	0.183769

$TS_{A7 \rightarrow A8}$

E(scf) = –2459.12223355 a.u.

ν_{min} = –302.7 cm^{-1}

N	-1.247076	-0.983834	-0.587582
C	0.118828	-1.141521	-0.775923
B	0.939531	0.066316	-0.265294
N	3.546474	-0.334747	-0.567328
C	0.545871	-2.368176	-1.377029
H	1.599689	-2.542176	-1.486310
N	2.925296	1.651413	0.128990
C	-0.322483	-3.304514	-1.818603
H	0.041875	-4.222313	-2.272390
C	-2.657402	-4.016461	-2.217902
H	-2.278088	-4.920227	-2.687019
C	-1.726499	-3.088434	-1.712627
C	-4.010791	-3.782617	-2.133193
H	-4.723445	-4.497730	-2.529917
C	-2.167729	-1.898834	-1.086315
C	-3.558304	-1.688741	-1.003180
H	-3.939265	-0.806424	-0.523065
C	-4.451305	-2.600193	-1.522408
H	-5.513337	-2.390290	-1.442699
C	-0.108314	1.000269	0.313442
H	0.023413	1.990224	0.738149

C	-2.528688	-2.601724	-2.230065	C	2.284484	2.925825	0.053629
C	-2.031837	-3.760954	-2.829810	C	2.152600	3.696962	1.220056
H	-2.528802	-4.708582	-2.637807	C	1.585525	4.969379	1.127852
C	-0.905017	-3.720988	-3.636499	H	1.461001	5.553243	2.036211
H	-0.518379	-4.632945	-4.080253	C	1.192568	5.492286	-0.093412
C	-0.270448	-2.506613	-3.874090	H	0.752412	6.482338	-0.152263
H	0.610888	-2.471438	-4.508816	C	1.377167	4.739561	-1.245349
C	-0.750553	-1.319910	-3.324089	H	1.093714	5.152400	-2.209466
C	-0.071545	-0.013558	-3.665234	C	1.916682	3.455675	-1.197803
H	0.966205	-0.222683	-3.944822	C	2.148084	2.715634	-2.492613
H	-0.030030	0.633501	-2.786253	H	1.413282	3.057730	-3.228783
C	-0.761461	0.727092	-4.815747	H	1.961134	1.649326	-2.354926
H	-0.231246	1.654804	-5.048843	C	3.553011	2.954118	-3.055549
H	-1.792028	0.989176	-4.557172	H	3.687655	2.429944	-4.006665
H	-0.792585	0.112455	-5.720695	H	4.324379	2.600593	-2.364089
C	-3.744605	-2.686409	-1.336434	H	3.728930	4.020453	-3.226228
H	-3.808781	-1.790692	-0.714205	C	2.644976	3.227092	2.566528
H	-3.616574	-3.517460	-0.635969	H	2.842210	2.156414	2.536080
C	-5.046143	-2.900683	-2.117776	H	1.850728	3.374413	3.307117
H	-5.904946	-2.926500	-1.440795	C	3.904300	3.972144	3.023573
H	-5.019408	-3.847149	-2.666402	H	4.247132	3.597920	3.992966
H	-5.212570	-2.105841	-2.851126	H	3.716975	5.045433	3.121297
C	-1.875559	0.611174	-0.961709	H	4.718165	3.846360	2.302563
C	-3.505521	1.831914	-2.138295	C	2.434029	0.411182	-0.258117
H	-3.023508	2.401109	-2.944008	C	4.799093	0.403429	-0.458704
H	-4.455197	2.308202	-1.889433	H	5.198230	0.650250	-1.452294
C	-3.648056	0.363945	-2.503681	H	5.549408	-0.196228	0.064285
H	-4.542355	-0.085349	-2.051399	C	4.373380	1.631799	0.327670
H	-3.680268	0.185952	-3.580921	H	4.622449	1.529526	1.392137
C	-2.988858	2.549351	0.181309	H	4.821383	2.556826	-0.042603
C	-3.613027	1.935855	1.283868	C	3.630788	-1.654567	-1.092067
C	-3.937683	2.735712	2.378725	C	3.731563	-2.729153	-0.192557
H	-4.412409	2.290831	3.246356	C	3.849344	-4.013724	-0.717936
C	-3.703087	4.105014	2.361577	H	3.906072	-4.867049	-0.051613
H	-3.962887	4.709063	3.225187	C	3.891667	-4.219390	-2.094035
C	-3.177400	4.706606	1.227095	H	3.986791	-5.227158	-2.486248

H	-3.037450	5.782065	1.210256	C	3.819802	-3.142236	-2.964438
C	-2.824995	3.945096	0.112343	H	3.854100	-3.305084	-4.038232
C	-2.300049	4.588650	-1.155586	C	3.688190	-1.839063	-2.479476
H	-3.131113	4.699282	-1.865604	C	3.522944	-0.700630	-3.457657
H	-1.595560	3.896921	-1.627253	H	4.231043	-0.840242	-4.282268
C	-1.623049	5.944541	-0.968711	H	3.776041	0.248403	-2.982213
H	-1.162993	6.268908	-1.905522	C	2.097864	-0.606615	-4.016630
H	-0.838475	5.889621	-0.207838	H	2.026207	0.207128	-4.744757
H	-2.331012	6.720829	-0.664377	H	1.377595	-0.415526	-3.216564
C	-3.998030	0.473744	1.225970	H	1.807305	-1.537199	-4.513032
H	-3.109904	-0.153544	1.353880	C	3.651137	-2.457863	1.291721
H	-4.357341	0.265491	0.211540	H	2.659028	-2.041195	1.501406
C	-5.077194	0.038945	2.211396	H	4.363713	-1.662064	1.542481
H	-5.312255	-1.016184	2.051794	C	3.900011	-3.660069	2.194950
H	-5.993424	0.625512	2.088321	H	3.911371	-3.347619	3.241911
H	-4.747234	0.134840	3.249140	H	4.862603	-4.136294	1.981908
N	2.874761	-1.196790	0.207590	H	3.110904	-4.410447	2.088403
C	4.111861	-0.667433	0.684358	N	-2.821839	1.047000	0.353331
C	4.129234	0.308485	1.698011	C	-3.811331	1.371790	-0.599361
C	5.361647	0.849379	2.071550	C	-5.174487	1.259051	-0.227683
H	5.386519	1.626665	2.829790	C	-6.170422	1.513863	-1.168860
C	6.548145	0.422076	1.495115	H	-7.211469	1.409693	-0.870780
H	7.492191	0.862857	1.799453	C	-5.858427	1.844432	-2.479333
C	6.517934	-0.566537	0.521453	H	-6.643636	2.021707	-3.207430
H	7.442064	-0.902306	0.057221	C	-4.523873	1.923210	-2.848731
C	5.310217	-1.116359	0.099099	H	-4.263271	2.172423	-3.874462
C	5.325994	-2.146808	-1.008571	C	-3.494290	1.700734	-1.933336
H	5.959392	-1.772181	-1.821801	C	-2.075987	1.913400	-2.402311
H	4.320506	-2.262362	-1.422557	H	-2.051800	1.808701	-3.492639
C	5.862523	-3.510548	-0.557851	H	-1.388857	1.162792	-2.005919
H	5.890081	-4.216066	-1.393450	C	-1.574582	3.305774	-2.017119
H	5.237511	-3.943381	0.228358	H	-0.612457	3.512470	-2.487097
H	6.876383	-3.417998	-0.156955	H	-1.439018	3.387755	-0.935050
C	2.879431	0.809477	2.393771	H	-2.285160	4.075995	-2.333444
H	1.995743	0.280451	2.034372	C	-5.606497	0.772932	1.141290
H	2.725416	1.862982	2.128821	H	-4.743779	0.374780	1.680105

C	2.936462	0.662604	3.917301
H	2.022952	1.056880	4.372300
H	3.783454	1.198171	4.355644
H	3.029538	-0.390990	4.200435
N	0.275936	-1.672817	1.249517
C	1.416828	-2.462639	1.704032
H	1.117102	-3.485345	1.965009
H	1.845310	-2.024275	2.621818
C	2.514451	-2.543871	0.655076
H	3.391155	-3.034865	1.083029
H	2.178978	-3.142026	-0.200855
C	-0.951449	-2.075961	1.847601
C	-1.461885	-1.281957	2.897081
C	-2.606785	-1.702431	3.569221
H	-2.998880	-1.112795	4.390995
C	-3.267866	-2.868295	3.195285
H	-4.170810	-3.171729	3.716781
C	-2.762200	-3.644793	2.165509
H	-3.266529	-4.566933	1.884149
C	-1.587287	-3.280769	1.499373
C	-1.007786	-4.238765	0.480745
H	-0.190386	-3.762348	-0.063616
H	-1.767074	-4.473538	-0.270188
C	-0.530458	-5.558446	1.099744
H	-0.118724	-6.218834	0.330482
H	-1.356632	-6.084190	1.588069
H	0.242317	-5.396559	1.856894
C	-0.740435	-0.003207	3.260781
H	-0.681258	0.620350	2.359992
H	0.299068	-0.254233	3.497358
C	-1.344299	0.806137	4.402260
H	-0.757556	1.714099	4.567655
H	-1.362410	0.243837	5.341996
H	-2.367741	1.120798	4.173242
B	1.763673	-0.453278	-0.245555

H	-6.294610	-0.070476	1.002495
C	-6.317906	1.839856	1.981570
H	-6.599256	1.442074	2.961310
H	-7.229004	2.186752	1.484494
H	-5.683668	2.717398	2.139858
N	-1.120469	0.135860	1.795273
C	-1.899988	1.109340	2.548432
H	-1.270887	1.748217	3.183635
H	-2.608064	0.582153	3.202170
C	-2.661900	1.933428	1.497183
H	-3.619637	2.278738	1.892187
H	-2.084812	2.835144	1.233358
C	-0.681144	-1.048696	2.449354
C	-1.532886	-2.174414	2.544617
C	-1.033693	-3.355641	3.090490
H	-1.673157	-4.230347	3.141952
C	0.258284	-3.428994	3.596609
H	0.630850	-4.357631	4.018354
C	1.045232	-2.290636	3.604024
H	2.030526	-2.311694	4.062262
C	0.592643	-1.095740	3.040027
C	1.442947	0.135804	3.203439
H	1.109641	0.898709	2.502528
H	2.484710	-0.099839	2.949088
C	1.375210	0.663943	4.641717
H	2.014113	1.542096	4.775171
H	1.696132	-0.098171	5.358524
H	0.349209	0.945194	4.899424
C	-3.002526	-2.066098	2.202267
H	-3.141656	-1.304495	1.437407
H	-3.514058	-1.671887	3.092714
C	-3.689890	-3.361829	1.779429
H	-4.712912	-3.152078	1.456601
H	-3.746610	-4.085231	2.598531
H	-3.168635	-3.832027	0.940309
B	-1.513865	0.299524	0.262214

A8

E(scf) = –2459.15708351 a.u.

N	-1.310833	-0.851667	-0.675135
C	-0.121926	-0.691910	-1.280098
B	0.812022	0.260433	-0.465040
N	3.261601	-0.379359	-1.092065
C	0.110998	-1.297286	-2.543200
H	1.054803	-1.106047	-3.036299
N	2.933882	1.708355	-0.533330
C	-0.837273	-2.079511	-3.128134
H	-0.668899	-2.538097	-4.099559
C	-3.057927	-3.173663	-2.976504
H	-2.882686	-3.652808	-3.935656
C	-2.062588	-2.313368	-2.457467
C	-4.213581	-3.407403	-2.276542
H	-4.971464	-4.075038	-2.672523
C	-2.269915	-1.668462	-1.211042
C	-3.466595	-1.927861	-0.509463
H	-3.604140	-1.450244	0.446351
C	-4.408610	-2.779073	-1.028332
H	-5.318131	-2.969522	-0.467203
C	0.046847	0.796016	0.632637
H	0.387660	1.523754	1.367321
C	2.338890	3.001893	-0.401895
C	2.454693	3.662881	0.826226
C	2.073546	5.004129	0.890099
H	2.152185	5.531290	1.836787
C	1.577681	5.656624	-0.228526
H	1.284252	6.699286	-0.164325
C	1.410338	4.959540	-1.419643
H	0.970726	5.456801	-2.279204
C	1.780686	3.620875	-1.532397
C	1.517330	2.875116	-2.822220

$TS_{A8\rightarrow7}$

E(scf) = –2459.17366826 a.u.

ν_{min} = –93.9 cm^{-1}

N	-0.607027	1.683849	-0.905095
C	-0.939702	0.474698	-1.353346
B	-0.294502	-0.676016	-0.504737
N	-1.839017	-2.776472	-0.681796
C	-1.774904	0.327986	-2.498723
H	-1.978333	-0.669841	-2.871719
N	0.312480	-3.161423	-0.644613
C	-2.295614	1.422828	-3.123574
H	-2.931729	1.325513	-4.000514
C	-2.520385	3.900845	-3.190444
H	-3.188330	3.821240	-4.044221
C	-1.998541	2.713373	-2.621335
C	-2.192620	5.126958	-2.672569
H	-2.598208	6.033190	-3.110328
C	-1.126414	2.791569	-1.502425
C	-0.800039	4.076731	-0.994156
H	-0.137711	4.131939	-0.139869
C	-1.320198	5.209923	-1.562620
H	-1.064278	6.182261	-1.153559
C	0.572984	-0.229409	0.546877
H	1.053986	-0.911536	1.248994
C	1.726199	-2.990513	-0.797782
C	2.544947	-3.337560	0.282353
C	3.927640	-3.324283	0.092908
H	4.578518	-3.588745	0.921929
C	4.472389	-2.948939	-1.123673
H	5.548984	-2.932140	-1.257955
C	3.640193	-2.546191	-2.161348
H	4.075739	-2.189681	-3.089328
C	2.254781	-2.557051	-2.026646

H	0.860603	3.499028	-3.436042	C	1.400007	-2.038368	-3.162815
H	0.943793	1.968840	-2.596194	H	2.077813	-1.655941	-3.931585
C	2.755668	2.506530	-3.646767	H	0.835735	-1.169979	-2.805283
H	2.454437	2.112211	-4.621402	C	0.443074	-3.043181	-3.813342
H	3.356262	1.733558	-3.159693	H	0.004379	-2.610317	-4.716866
H	3.394340	3.378497	-3.817264	H	-0.385604	-3.312559	-3.152890
C	2.939116	2.962680	2.075833	H	0.964164	-3.962586	-4.098323
H	3.016691	1.887039	1.894263	C	1.986528	-3.668187	1.648403
H	2.165581	3.077763	2.844021	H	0.910859	-3.472737	1.671046
C	4.263972	3.511702	2.615820	H	2.428815	-2.968303	2.367861
H	4.568484	2.972875	3.517133	C	2.271358	-5.103826	2.101511
H	4.176082	4.571811	2.870658	H	1.831423	-5.295032	3.084181
H	5.067221	3.422867	1.877399	H	3.346654	-5.290504	2.173213
C	2.325392	0.528790	-0.731849	H	1.862906	-5.835379	1.396488
C	4.620912	0.171409	-1.066971	C	-0.620112	-2.202539	-0.647049
H	5.098712	0.060730	-2.045451	C	-1.763152	-4.234287	-0.541168
H	5.219441	-0.371308	-0.329146	H	-2.375846	-4.731349	-1.297380
C	4.391703	1.634311	-0.673973	H	-2.134304	-4.520907	0.449483
H	4.878153	1.894834	0.270145	C	-0.264039	-4.508460	-0.706002
H	4.725911	2.341932	-1.437431	H	0.140177	-5.140471	0.087705
C	3.098753	-1.797503	-1.001968	H	-0.020107	-4.968415	-1.669147
C	2.925986	-2.380225	0.260714	C	-3.051728	-2.092214	-0.348329
C	2.761640	-3.765184	0.314760	C	-3.226750	-1.600889	0.953694
H	2.578994	-4.234886	1.276152	C	-4.405316	-0.904047	1.225990
C	2.809353	-4.535511	-0.836598	H	-4.538976	-0.466203	2.210396
H	2.668179	-5.609805	-0.773429	C	-5.382331	-0.744293	0.256287
C	3.040692	-3.939618	-2.071215	H	-6.287869	-0.191793	0.486353
H	3.091235	-4.558605	-2.960411	C	-5.209870	-1.290542	-1.010286
C	3.195840	-2.559682	-2.177362	H	-5.990061	-1.169040	-1.753809
C	3.488532	-1.895002	-3.507625	C	-4.042337	-1.975539	-1.337042
H	4.577392	-1.825219	-3.635898	C	-3.852937	-2.604779	-2.702393
H	3.132453	-0.859799	-3.470746	H	-4.238833	-3.633193	-2.672213
C	2.891019	-2.593874	-4.728877	H	-2.780985	-2.698841	-2.903003
H	3.037343	-1.985351	-5.624896	C	-4.520427	-1.860030	-3.858641
H	1.817740	-2.760820	-4.598274	H	-4.227787	-2.303079	-4.814132
H	3.358766	-3.564546	-4.914121	H	-4.228604	-0.805802	-3.862494

C	2.938100	-1.580552	1.542457	H	-5.611216	-1.904771	-3.798904
H	1.942499	-1.619309	1.989667	C	-2.219938	-1.807176	2.062241
H	3.122516	-0.524675	1.336561	H	-1.795386	-0.837182	2.334255
C	3.984026	-2.073436	2.544890	H	-1.374055	-2.400729	1.709813
H	3.947786	-1.463493	3.451563	C	-2.819289	-2.486089	3.295825
H	4.994580	-2.016275	2.125539	H	-2.048505	-2.617375	4.060345
H	3.802022	-3.108557	2.844470	H	-3.236263	-3.468788	3.049728
N	-2.727374	0.919971	0.707309	H	-3.615260	-1.883828	3.740816
C	-3.435787	1.501776	-0.366337	N	2.054790	1.966552	0.397468
C	-4.781630	1.147395	-0.648982	C	3.101398	1.538295	-0.462805
C	-5.401853	1.642087	-1.798721	C	3.219559	2.044342	-1.771421
H	-6.422938	1.330435	-2.008984	C	4.291448	1.647220	-2.574141
C	-4.751386	2.493708	-2.674881	H	4.365707	2.044815	-3.584009
H	-5.244821	2.849400	-3.574080	C	5.254265	0.770216	-2.100791
C	-3.467865	2.910439	-2.355519	H	6.088813	0.474921	-2.730306
H	-2.959670	3.629350	-2.993965	C	5.134119	0.268648	-0.811507
C	-2.815720	2.459875	-1.208305	H	5.878066	-0.426367	-0.430863
C	-1.502059	3.108635	-0.850143	C	4.068869	0.627337	0.012932
H	-1.225274	3.795441	-1.658779	C	4.011783	0.043455	1.404117
H	-0.703682	2.368805	-0.774341	H	4.620056	-0.868016	1.416519
C	-1.560217	3.890403	0.464740	H	2.990100	-0.264722	1.629127
H	-0.609759	4.396357	0.649759	C	4.516207	0.993892	2.493384
H	-1.751602	3.215978	1.301105	H	4.559713	0.486048	3.462120
H	-2.356053	4.642084	0.441792	H	3.853442	1.856240	2.600701
C	-5.657751	0.282657	0.237627	H	5.518911	1.363633	2.257379
H	-5.070263	-0.263638	0.973434	C	2.223752	3.029096	-2.328374
H	-6.143498	-0.475697	-0.387258	H	1.351742	3.043909	-1.682384
C	-6.740523	1.093453	0.960753	H	1.877795	2.679477	-3.308763
H	-7.358265	0.447747	1.593089	C	2.786102	4.446740	-2.468094
H	-7.397223	1.597388	0.245407	H	2.011227	5.135338	-2.819331
H	-6.298183	1.868797	1.594294	H	3.619676	4.478983	-3.177253
N	-1.636999	-0.702754	2.016840	H	3.158544	4.814927	-1.506801
C	-2.847789	-0.295267	2.714525	N	0.229364	2.123415	1.826042
H	-2.679321	-0.193669	3.793323	C	1.131717	3.211092	2.180252
H	-3.667660	-1.030058	2.595564	H	1.660976	3.000081	3.125025
C	-3.210491	1.040092	2.072069	H	0.602405	4.164634	2.320188

H	-4.277658	1.263524	2.127410	C	2.129617	3.285105	1.008451
H	-2.697959	1.857774	2.613032	H	1.852401	4.088619	0.307264
C	-0.665408	-1.398091	2.777339	H	3.146052	3.511755	1.352345
C	-0.517686	-2.793710	2.621729	C	-0.665716	1.643345	2.817751
C	0.419256	-3.485502	3.393803	C	-2.013500	2.053500	2.810362
H	0.514229	-4.561703	3.265312	C	-2.892460	1.551567	3.773314
C	1.196560	-2.829193	4.337152	H	-3.930242	1.877621	3.757108
H	1.914743	-3.378544	4.938634	C	-2.459447	0.664634	4.748572
C	1.040196	-1.459674	4.507287	H	-3.153371	0.281633	5.491101
H	1.646304	-0.936893	5.244225	C	-1.125881	0.277768	4.769072
C	0.121252	-0.733152	3.748293	H	-0.778440	-0.418011	5.529435
C	-0.009794	0.750120	4.015983	C	-0.218449	0.758842	3.824345
H	-0.672687	1.198462	3.276698	C	1.224196	0.316160	3.919557
H	0.972584	1.219858	3.876327	H	1.778122	0.691065	3.059941
C	-0.523206	1.069190	5.423976	H	1.267014	-0.779496	3.861406
H	-0.623625	2.149658	5.565386	C	1.908396	0.784923	5.207244
H	0.153193	0.689466	6.196072	H	2.949350	0.449876	5.239780
H	-1.503567	0.614155	5.594685	H	1.402862	0.398911	6.097848
C	-1.389145	-3.572499	1.667233	H	1.902354	1.877341	5.273021
H	-2.222676	-2.937945	1.370941	C	-2.533972	3.033561	1.788729
H	-1.810830	-4.435149	2.197608	H	-1.686615	3.465193	1.259879
C	-0.655251	-4.068570	0.418984	H	-3.043661	3.853150	2.311007
H	-1.358740	-4.517394	-0.289729	C	-3.496807	2.407569	0.776857
H	0.098663	-4.820073	0.673847	H	-3.774417	3.135503	0.008130
H	-0.137675	-3.248552	-0.085759	H	-4.413529	2.057129	1.262405
B	-1.418332	0.107330	0.742338	H	-3.035882	1.547755	0.284072
				B	0.795078	1.311326	0.739071

7

E(scf) = –2459.19919075 a.u.

N	-2.614857	0.009279	-0.697835
C	-1.697085	0.067020	0.256383
B	-0.245390	-0.465897	-0.084516
N	-0.829430	-2.993465	0.065355
C	-2.021504	0.513459	1.573433

H	-1.234994	0.532558	2.319909
N	0.539066	-2.472621	-1.550527
C	-3.302795	0.858969	1.885573
H	-3.576775	1.162848	2.892870
C	-5.643699	1.201011	1.085023
H	-5.950367	1.514517	2.079708
C	-4.292470	0.833328	0.870193
C	-6.548067	1.169229	0.053234
H	-7.580887	1.455800	0.224267
C	-3.884572	0.418245	-0.422949
C	-4.837578	0.397873	-1.471610
H	-4.494631	0.087239	-2.453267
C	-6.137580	0.766979	-1.239762
H	-6.861061	0.750898	-2.049083
C	1.026620	0.189601	0.104551
H	1.927030	-0.402572	-0.084677
C	1.246959	-1.722521	-2.543345
C	2.646670	-1.725224	-2.497856
C	3.338448	-1.079217	-3.523200
H	4.423802	-1.060559	-3.499911
C	2.656776	-0.450878	-4.554298
H	3.206617	0.047496	-5.346290
C	1.267621	-0.439911	-4.559808
H	0.737336	0.081099	-5.351624
C	0.528644	-1.059604	-3.552253
C	-0.977071	-0.910309	-3.543063
H	-1.241478	-0.254196	-4.378830
H	-1.295364	-0.379227	-2.637027
C	-1.798462	-2.198216	-3.660649
H	-2.850238	-1.948878	-3.825965
H	-1.761533	-2.793729	-2.744915
H	-1.461626	-2.821563	-4.495648
C	3.420095	-2.356999	-1.361979
H	2.737187	-2.658710	-0.563255
H	4.064032	-1.591491	-0.914419
C	4.281469	-3.546634	-1.796725

H	4.821422	-3.964235	-0.942466
H	5.019767	-3.247551	-2.546697
H	3.673807	-4.342482	-2.240346
C	-0.194329	-1.987159	-0.547497
C	-0.491710	-4.302779	-0.500136
H	-1.393562	-4.820856	-0.837172
H	-0.003990	-4.921262	0.259616
C	0.449777	-3.933656	-1.658426
H	1.441351	-4.382894	-1.555239
H	0.047568	-4.207534	-2.637505
C	-1.549561	-2.863753	1.296755
C	-0.820655	-2.704415	2.485668
C	-1.548434	-2.559802	3.666661
H	-1.027599	-2.430814	4.608621
C	-2.937189	-2.578636	3.652591
H	-3.485146	-2.458484	4.582062
C	-3.632008	-2.749523	2.462314
H	-4.715770	-2.748985	2.471997
C	-2.950413	-2.899630	1.256000
C	-3.670566	-3.056441	-0.067091
H	-3.463172	-4.057027	-0.470617
H	-3.232890	-2.342898	-0.773575
C	-5.181694	-2.849284	-0.021160
H	-5.594496	-2.908096	-1.031268
H	-5.435219	-1.863112	0.378678
H	-5.680929	-3.609794	0.588018
C	0.694112	-2.652791	2.456309
H	0.992370	-1.749490	1.908638
H	1.069455	-3.497478	1.863544
C	1.386652	-2.650841	3.814273
H	2.469282	-2.590234	3.673208
H	1.162325	-3.551352	4.395092
H	1.091272	-1.781073	4.408578
N	0.611479	2.845049	0.333936
C	-0.517342	3.055147	-0.498227
C	-1.729679	3.519911	0.044423

C	-2.843527	3.659009	-0.785082
H	-3.784461	3.987083	-0.349719
C	-2.775808	3.346228	-2.133231
H	-3.657681	3.430973	-2.760343
C	-1.570516	2.910652	-2.667440
H	-1.505478	2.672741	-3.726332
C	-0.428845	2.772694	-1.878295
C	0.858616	2.333443	-2.538856
H	0.627763	2.046564	-3.569751
H	1.232428	1.427663	-2.052624
C	1.957317	3.398748	-2.565613
H	2.833417	3.024723	-3.106257
H	2.278450	3.661577	-1.555220
H	1.612300	4.311765	-3.061210
C	-1.874311	3.882045	1.503411
H	-1.121019	3.350830	2.091871
H	-2.848061	3.524123	1.854454
C	-1.790266	5.393754	1.746712
H	-1.885648	5.628558	2.811681
H	-2.591556	5.912767	1.211436
H	-0.841318	5.804431	1.390183
N	2.570582	1.992061	1.192898
C	2.720684	3.436189	1.284743
H	3.469326	3.813400	0.569640
H	3.048077	3.753032	2.283027
C	1.309728	3.970916	0.957031
H	0.810358	4.300673	1.877880
H	1.347744	4.833386	0.280904
C	3.617303	1.112989	1.559026
C	3.627984	0.569640	2.854374
C	4.652280	-0.311130	3.204236
H	4.686459	-0.736313	4.202183
C	5.638966	-0.648780	2.286075
H	6.434696	-1.330046	2.572436
C	5.609192	-0.120680	1.000707
H	6.383293	-0.401858	0.293909

C	4.597076	0.759679	0.614560
C	4.484331	1.293508	-0.799188
H	4.344305	2.378628	-0.766018
H	3.557146	0.903169	-1.239345
C	5.652874	0.976274	-1.725887
H	5.468399	1.398931	-2.717459
H	5.794908	-0.102780	-1.850700
H	6.592595	1.392744	-1.349150
C	2.503947	0.924915	3.804916
H	1.585381	0.453589	3.429109
H	2.312557	2.000579	3.737118
C	2.723880	0.545421	5.266236
H	1.879564	0.881262	5.874899
H	3.631581	1.009586	5.665073
H	2.815252	-0.536563	5.401977
B	1.346735	1.623576	0.541290

References

[1] *Manipulation of Air Sensitive Compounds*; 2nd ed.; D. F. Shriver and M. A. Drezdzon, Eds.; Wiley: New York, 1986.

[2] Purification of Laboratory Chemicals; 6th ed.; W. L. F. Armarego and C. L. L. Chai; Elsevier: Oxford, 2009.

[3] M. Arrowsmith, J. Böhnke, H. Braunschweig, M. A. Celik, T. Dellermann, K. Hammond, *Chem. Eur. J.* **2016**, *22*, 17169–17172.

[4] J. Böhnke, H. Braunschweig, W. C. Ewing, C. Hörl, T. Kramer, I. Krummenacher, J. Mies, A. Vargas, *Angew. Chem. Int. Ed.* **2014**, *53*, 9082–9085.

[5] G. Sheldrick, *Acta Cryst.* **2015**, *A71*, 3–8.

[6] G. Sheldrick, *Acta Cryst.* **2008**, *A64*, 112–122.

[7] M. J. Frisch, G. W. Trucks, H. B. Schlegel, G. E. Scuseria, M. A. Robb, J. R. Cheeseman, G. Scalmani, V. Barone, B. Mennucci, G. A. Petersson, H. Nakatsuji, M. Caricato, X. Li, H. P. Hratchian, A. F. Izmaylov, J. Bloino, G. Zheng, J. L. Sonnenberg, M. Hada, M. Ehara, K. Toyota, R. Fukuda, J. Hasegawa, M. Ishida, T. Nakajima, Y. Honda, O. Kitao, H. Nakai, T. Vreven, J. A. Montgomery, Jr., J. E. Peralta, F. Ogliaro, M. Bearpark, J. J. Heyd, E. Brothers, K. N. Kudin, V. N. Staroverov, T. Keith, R. Kobayashi, J. Normand, K. Raghavachari, A. Rendell, J. C. Burant, S. S. Iyengar, J. Tomasi, M. Cossi, N. Rega, J. M. Millam, M. Klene, J. E. Knox, J. B. Cross, V. Bakken, C. Adamo, J. Jaramillo, R. Gomperts, R. E. Stratmann, O. Yazyev, A. J. Austin, R. Cammi, C. Pomelli, J. W. Ochterski, R. L. Martin, K. Morokuma, V. G. Zakrzewski, G. A. Voth, P. Salvador, J. J. Dannenberg, S. Dapprich, A. D. Daniels, O. Farkas, J. B. Foresman, J. V. Ortiz, J. Cioslowski, D. J. Fox, Gaussian, Inc., Gaussian G09, Revision E.01, Wallingford CT, 2013.

[8] J.-D. Chai, M. Head-Gordon, *Phys. Chem. Chem. Phys.* **2008**, *10*, 6615–6620.

[9] Z. Chen, C. S. Wannere, C. Corminboeuf, R. Puchta, P. von R. Schleyer, *Chem. Rev.* **2005**, *105*, 3842–3888.

[10] K. Wolinski, J. F. Hinton, P. Pulay, *J. Am. Chem. Soc.* **1990**, *112*, 8251–8260.

[11] M. Cossi, V. Barone, B. Mennucci, J. Tomasi, *Chem. Phys. Lett.* **1998**, *286*, 253–260.

[12] A. V. Marenich, C. J. Cramer, D. G. Truhlar, *J. Phys. Chem. B* **2009**, *113*, 6378–6396.
